# Comparative Analysis of the Transcriptome, Proteome, and miRNA Profile of Kupffer Cells and Monocytes

**DOI:** 10.3390/biomedicines8120627

**Published:** 2020-12-18

**Authors:** Andrey Elchaninov, Anastasia Lokhonina, Maria Nikitina, Polina Vishnyakova, Andrey Makarov, Irina Arutyunyan, Anastasiya Poltavets, Evgenia Kananykhina, Sergey Kovalchuk, Evgeny Karpulevich, Galina Bolshakova, Gennady Sukhikh, Timur Fatkhudinov

**Affiliations:** 1 Laboratory of Regenerative Medicine, National Medical Research Center for Obstetrics, Gynecology and Perinatology Named after Academician V.I. Kulakov of Ministry of Healthcare of Russian Federation, 117997 Moscow, Russia; nastya.serbsky@gmail.com (A.L.); vpa2002@mail.ru (P.V.); anvitmak@yandex.ru (A.M.); labrosta@yandex.ru (I.A.); a.s.poltavets@gmail.com (A.P.); g_sukhikh@oparina4.ru (G.S.); 2 Histology Department, Medical Institute, Peoples’ Friendship University of Russia, 117198 Moscow, Russia; tfat@yandex.ru; 3 Laboratory of Growth and Development, Scientific Research Institute of Human Morphology, 117418 Moscow, Russia; mary.krutikova@gmail.com (M.N.); e.kananykhina@gmail.com (E.K.); gbolshakova@gmail.com (G.B.); 4 Laboratory of Bioinformatic Methods for Combinatorial Chemistry and Biology, Shemyakin-Ovchinnikov Institute of Bioorganic Chemistry of the Russian Academy of Sciences, 117997 Moscow, Russia; xerx222@gmail.com; 5 Information Systems Department, Ivannikov Institute for System Programming of the Russian Academy of Sciences, 109004 Moscow, Russia; karpulevich@ispras.ru; 6 Genome Engineering Laboratory, Moscow Institute of Physics and Technology, 141701 Dolgoprudny, Russia

**Keywords:** monocytes, macrophages, proteome, microRNAs, Kupffer cells, nanostring gene expression assay

## Abstract

Macrophage populations in most mammalian organs consist of cells of different origin. Resident macrophages originate from erythromyeloid precursors of the yolk sac wall; maintenance of the numbers of such macrophages in postnatal ontogenesis is practically independent of bone marrow haematopoiesis. The largest populations of the resident macrophages of embryonic origin are found in the central nervous system (microglia) and liver (Kupffer cells). In contrast, skin dermis and mucous membranes become predominantly colonized by bone marrow-derived monocytes that show pronounced functional and phenotypic plasticity. In the present study, we compared Kupffer cells and monocytes using the immunophenotype, gene expression profile, proteome, and pool of microRNA. The observed differences did not consider the resident liver macrophages as purely M2 macrophages or state that monocytes have purely M1 features. Monocytes show signs of high plasticity and sensitivity to pathogen-associated molecular patterns (e.g., high levels of transcription for *Tlr 2*, *4*, *7*, and *8*). In contrast, the resident liver macrophages were clearly involved in the regulation of specific organ functions (nitrogen metabolism, complement system protein synthesis).

## 1. Introduction

The role of macrophages in the maintenance of tissue and cellular homeostasis is difficult to overestimate. Being an important part of innate immunity, macrophages also regulate their microenvironment and the life events of surrounding cells, such as proliferation, apoptosis, metabolic rate, and changes in extracellular architecture [1]. Such a variety of functions is explained by the flexibility of macrophage phenotypes and the presence of different subpopulations of macrophages within one tissue [2,3], which originate from yolk sac hemopoietic cells or from monocytes of bone marrow origin [4,5]. It is thought that these subpopulations have different functionalities [6].

As a rule, mammalian organs contain a mixed population of macrophages from different sources of origin, with the exception of the central nervous system and liver, from where the macrophages of monocytic origin are almost completely absent. The proportion of bone marrow macrophages in the liver of mice may constitute about 7% [7], whereas other authors (by using a different, more specific set of markers) reduce this estimation to 2–5% [5,8]. The highest numbers of macrophages derived from monocytes are found in mucous membranes of the digestive tract and airways [1].

The reasons for such distribution and the very coexistence of the two separate macrophage lineages of different origin in mammals remains poorly understood. Presumably, the self-sustaining resident macrophages and monocyte-derived transient macrophages differ by their functional properties and their roles in repair processes [9,10].

Some researchers suggest that differences in the plasticity of macrophages of different origin contribute to their functional divergence and are the reason for the preservation of both populations of macrophages for the needs of the body. Moreover, in recent years, it has been increasingly argued that macrophages of bone marrow (monocytic) origin in mammals have higher plasticity. This point of view is supported by experimental studies on the replacement of resident macrophages with bone marrow macrophages after artificial depletion [11,12,13,14].

A distinctive feature of macrophage differentiation is the presence of a well-defined intermediate stage, called monocytes. The only exception is microglia, for which the monocyte-like stage has not been described [8]. Comparison of the properties of monocytes and mature macrophages reveals specific developmental features of certain phenotypes in macrophages in the course of differentiation. The aim of this study was to compare immunophenotypes, as well as gene expression, proteome, and microRNA (miRNA) profiles of monocytes and Kupffer cells.

## 2. Results

### 2.1. Comparative Immunophenotyping of KUPFFER Cells and Monocytes

**Figure 1:**
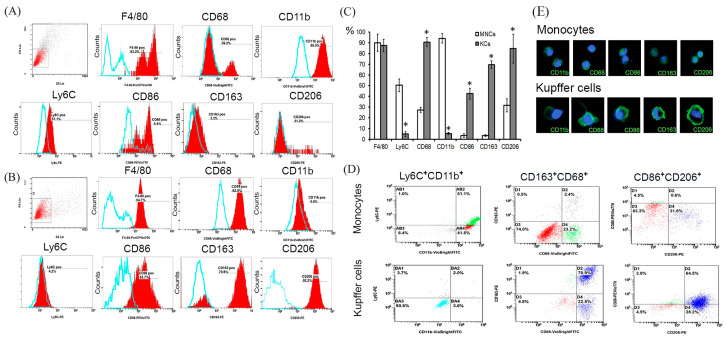
Immunophenotyping of Kupffer cells and monocytes by flow cytometry (**A**–**D**) and immunocytochemistry (**E**). Monocyte immunophenotypes (**A**,**C**,**D**,**E**), Kupffer cell immunophenotypes (**B**–**E**), the percentages of positive cells are indicated. The blue curve corresponds to the isotype control. Dot-plots of double positive cells are shown (**D**). Fluorescent microscopy images display immunostaining (FITC, green), cell nuclei are counterstained with 4′,6-diamidino-2-phenylindole (DAPI) (blue), ×400 (**E**). Comparative analysis of markers expression in monocytes and Kupffer cells according to the flow cytometry assessment (**C**). The data are listed as mean ± standard deviation. * *p*  <  0.05 monocytes vs Kupffer cells. KCs: Kupffer cells; MNCs: monocytes.

The results indicated that Kupffer cells differ from monocytes by the expression of several markers (Figure 1A–E, Table 1). Over 90% of the monocytes were CD11b^+^, whereas only 5% of the Kupffer cells of the intact liver expressed CD11b in detectable quantities (*p* = 0.004). Similar data were obtained regarding the expression of Ly6C, where only 5% of the liver macrophages were Ly6C^+^ (*p* = 0.005). Expression of CD68 (*p* = 0.024), CD86 (*p* = 0.016), CD163 (*p* = 0.036), and CD206 (*p* = 0.036) by Kupffer cells was more pronounced, and no differences were revealed for F4/80 (*p =* 0.527). Magnetic enrichment whose results were described above revealed a more pronounced difference in the increase of CD68^+^ and CD206^+^ Kupffer cells than when comparing samples from unsorted blood and liver (Figures S1 and S2).

### 2.2. Comparative Gene Expression Profiling by Nanostring

**Figure 2:**
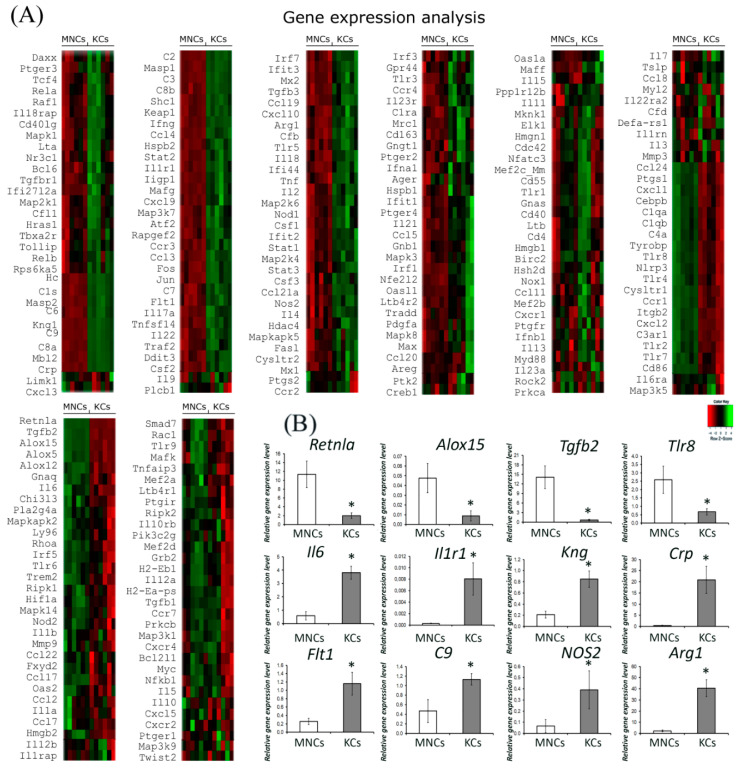
Gene expression analysis in monocytes and Kupffer. Heatmaps depict relative expression levels of inflammation-related genes (**A**). Gene expression profiles (PCR-RT) for MNCs and KCs (**B**). The data are presented as means with the bars for standard deviations. The data are listed as mean ± standard deviation. * *p*  <  0.05 monocytes vs Kupffer cells. KCs: Kupffer cells; MNCs: monocytes.

We compared the expression profile of genes associated with inflammation and found pronounced differences between Kupffer cells and monocytes. In Kupffer cells, the expression of 84 genes was more than 2-fold different; the expression of 51 genes was up-regulated and 33 genes were down-regulated compared to monocytes (Figure 2A,B). Notably, in monocytes, the expression of toll-like receptor genes 2, 4, 7, and 8, as well as *Retnla* (Resistin Like Alpha), *Alox15*, and *Alox12* was more than 2-times higher than in Kupffer cells. In Kupffer cells, we found more than a 15-fold higher expression of genes associated with the complement system (Table S4); in addition, the expression of *Arg1* and *NOS3* was significantly increased compared with monocytes (Figure 2A). Another set of 53 genes showed similar expression levels in monocytes and Kupffer cells (Figure 2A). In all cases, the results of RT-PCR for the mRNA targets listed in Table 2 confirmed the differences between Kupffer cells and monocytes revealed by the nanostring approach (Figure 2B).

### 2.3. Comparative miRNA Expression Profiling by Nanostring

**Figure 3:**
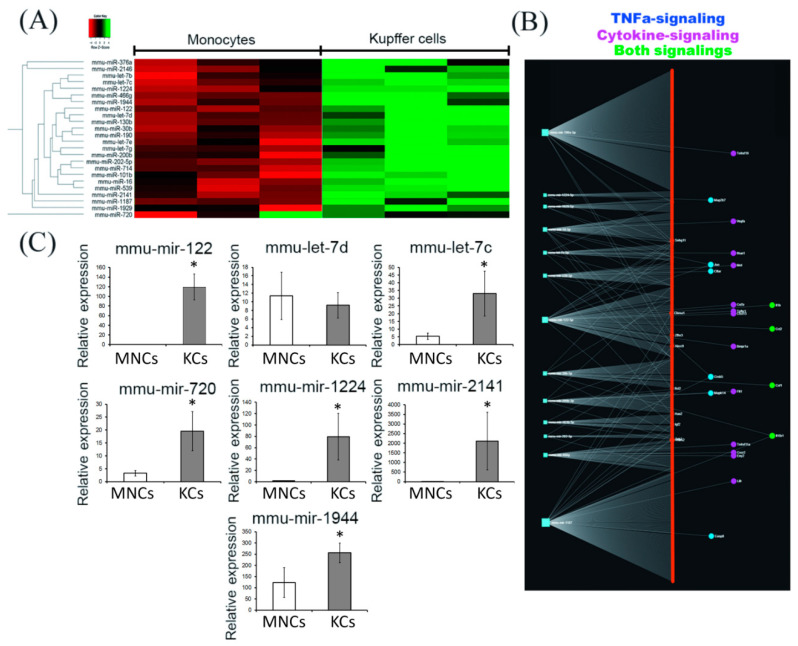
The analysis of miRNA expression in monocytes and Kupffer cells. Heatmaps depict relative expression levels of miRNAs (**A**). Full-length heatmaps are listed in Figure S3. The miRNAs with higher expression levels in Kupffer cells, are shown with their highly correlated targets that are predicted by TargetScan and miRNet. The colour labels are blue (genes of TNFa-signalling), pink (cytokine signalling), and green (both types of signalling) (**B**). miRNA expression profiles (PCR-RT) for MNCs and KCs (**C**). The data is presented as means with the bars for standard deviations. Data is listed as mean ± standard deviation. * *p*  <  0.05 monocytes vs Kupffer cells. KCs: Kupffer cells; MNCs: monocytes.

Of the 577 miRNAs presented in the panel, 24 miRNAs showed a statistically significant increase in expression in Kupffer cells compared with blood monocytes (Figure 3A and Figure S3, Table S5). Enrichment analysis of the genes identified as the targets of miRNAs revealed that a number of genes regulated by miRNAs elevated in Kupffer cells are involved in signalling pathways associated with the TNFa-induced cascade (*Map2k7*, *Jun*, *Cflar*, *Creb5*, *Mapk14*, *Casp8*), cytokine signalling (*Vegf*, *Ifnar1*, *Met*, *Csf3r*, *TGFbr1*, *Cxcl13*, *Bmpr1a*, *Flt1*, *Tnfrsf11a*, *Cxcr2*, *Eny2*, *Lifr2*), and both types of signalling (*Il1b*, *Ccl2*, *Csf1*, *Il18r1*) (Figure 3B). Full-length overexpressed microRNA targets in Kupffer cells are provided in the Supplementary Materials (Table S3). According to the nanostring data (Figure 2A), certain genes, presumably controlled by the studied microRNA, were indeed upregulated in Kupffer cells (e.g., *Map2k7*, *Flt1*, *Csf1*, and *Jun*); however, *CCL2* was expressed at a higher level in monocytes.

Of the 7 miRNAs whose expression was verified by RT-PCR, increased expression in Kupffer cells was confirmed for mmu-mir-122 (*p* = 0.002), mmu-let-7c (*p* = 0.01), mmu-mir-720 *p* = 0.01, mmu-mir-1224 (*p* = 0.004), mmu-mir-2141(*p* = 0.01), and mmu-mir-1944 (*p* = 0.041) (Figure 3C).

In our opinion, it is necessary to make one remark of a methodological nature. To study the same phenomenon (assessing the level of miRNA expression), we used two approaches, nanostring and real-time PCR. Some of the results obtained by the nanostring method were confirmed by real-time PCR, however, in some cases, the data did not coincide (mmu-let-7d, *p* = 0.732). This could be explained by technical differences in sample preparation for the analysis (additional stages of miRNA extension and the stage of reverse transcription in real-time PCR), as well as by the method of calculating and normalizing the relative expression levels (different miRNAs served as references for nanostring and real-time PCR).

### 2.4. Comparative Proteome Analysis

**Figure 4:**
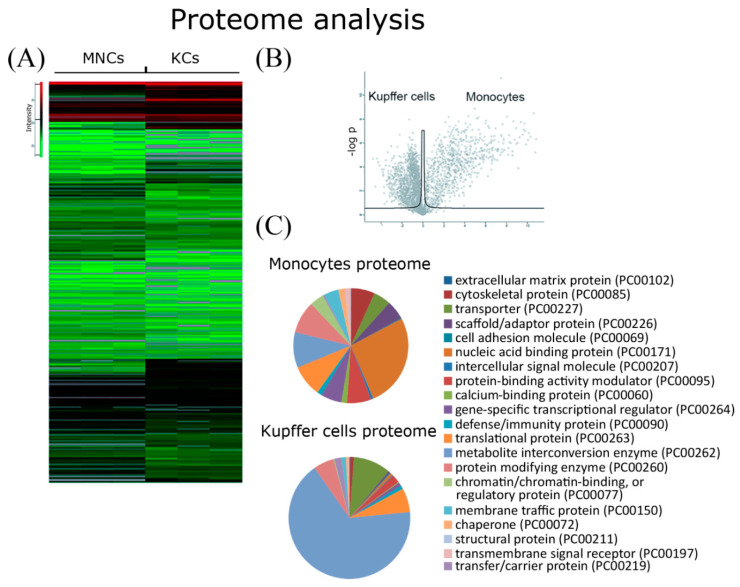
Proteome analysis for monocytes and Kupffer cells. Heatmaps presenting differentially expressed proteins (**A**). A scatter plot showing the difference in the expression of proteins associated with monocytes and Kupffer cells (**B**). Pie charts of the functional categories of the proteome for monocytes and Kupffer cells (**C**). Pie charts of the protein class categories of the proteome for monocytes and Kupffer cells are listed in Figure S4.

Proteome analysis of Kupffer cells and blood monocyte samples identified 2797 proteins. For 1464 proteins, a statistically significant 2-fold difference between Kupffer cells and monocytes was found (Figure 4A–C and Figure S4). Gene Ontology cell component analysis of the proteins expressed differentially in Kupffer cells and monocytes unambiguously showed the enrichment of Kupffer cells by the proteins involved in metabolism (Panther protein class: PC00262). In energy metabolism, located in mitochondria and lipid metabolism, out of 700 proteins significantly increased in Kupffer cells > 2-fold, more than 70% were related to mitochondria (Panther protein class of molecular function: GO:0003824, GO:0044237, GO:0008152, GO:0006091, GO:0046034) (Figure 4C and Figure S4). At the same time, Kupffer cells showed a distinct decrease in the expression of many proteins related to other cell components, such as nuclear and cytosolic proteins. Both Kupffer cells and monocytes also expressed several proteins related to extracellular exosomes, with more than 170 exosome proteins increased in monocytes and 234 exosome-related proteins increased in Kupffer cells (Figure 4C and Figure S4).

Gene Ontology biological processes analysis showed that the proteins most increased in monocytes are involved in all stages of protein synthesis processes, such as transcription, translation, and their regulation (Panther class: GO:0045182, GO:0140110) (Figure S4). Some of the proteins with expression specifically increased in monocytes was related to the cell cycle (Panther class: GO:0007049), cell division (Panther class: GO:0051301), cell adhesion (Panther class: PC00069), and cellular response to stimuli (Panther class: GO:0051716) (Figure S4).

Proteins up-regulated in Kupffer cells vs monocytes and belonging to the PC00262 group (metabolite interconversion enzyme) are available in Table S1. Proteins up-regulated in monocytes vs. Kupffer cells and belonging to the PC00171 group (nucleic acid binding protein) are available in Table S2.

Comparative analysis of the gene expression profile and proteomic analysis data revealed that two genes *Iigp1* and *Arg1* were overexpressed in Kupffer cells, and the corresponding proteins (codenamed Q9QZ85 and Q61176) were significantly overrepresented in Kupffer cells. In total, five matched genes, *Alox15*, *Alox5*, *Chil3*, *Itgb2*, and *Hmgb2,* were overexpressed in monocytes, and their protein products (codenamed P39654, P48999, O35744, P11835, and P30681, respectively) were significantly overrepresented in monocytes.

After comparison of mmu-let-7 miRNA targets received from TargetScan, it was found that a target gene *Agxt2* correlated with its protein, Q3UEG6, from our proteome data that was significantly increased in Kupffer cells.

### 2.5. Western-Blot Analysis of Protein Representation

**Figure 5:**
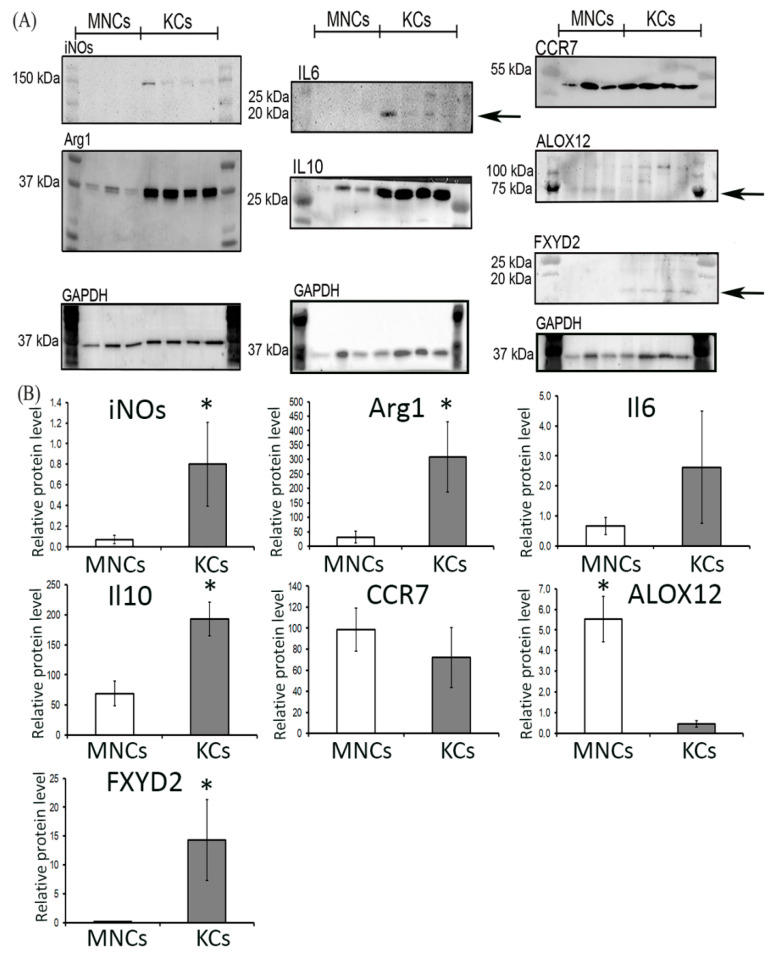
Relative protein levels of iNOs, IL6, IL10, CCR7, and Arg1 revealed by Western-Blot analysis. Representative Western-Blot membranes are shown in the panel (**A**), full-size membranes are shown in Figures S5 and S6. Visual assessment of the western blot membranes was followed by quantitative densitometry (**B**). The data is listed as mean ± standard deviation. * *p*  <  0.05 monocytes vs Kupffer cells. KCs: Kupffer cells; MNCs: monocytes.

As revealed by Western-Blot analysis, iNOs, Arg1, and IL10 protein expression was significantly higher in Kupffer cells (*p* = 0.013, *p* = 0.03 and *p* = 0.022 respectively), while ALOX12 protein expression was significantly higher in monocytes (*p* =0.007) (Figure 5A,B and Figure S5). We did not reveal any significant difference in the levels of CCR7 (*p* = 0.517), IL6 (*p* = 0.421), and FXYD2 (*p* = 0.057) between Kupffer cells and monocytes. Full-size membrane images after the blotting of polyacrylamide gel are provided in the Supplementary Materials (Figures S5 and S6).

## 3. Discussion

As a rule, mammalian organs contain mixed populations of macrophages from different sources of origin, with the exception of the central nervous system and liver, from which the macrophages of bone marrow origin are almost completely absent [4,15]. Study of mouse liver macrophage phenotypes confirmed this statement: the proportion of monocytic macrophages (Ly6C^+^CD11b^+^) was less than 10%.

The reasons for the coexistence of several subpopulations of macrophages of different origin in the body of mammals remain obscure. Several assumptions have been made to explain this phenomenon. First of all, it is possible that the resident macrophages of embryonic origin and macrophages derived from monocytes may differ in their functional capabilities and their roles in repair processes [10,16]. That is, the macrophages of bone marrow (monocytic) origin synthesize predominantly pro-inflammatory cytokines, whereas the resident macrophages produce factors that regulate homeostasis of organs under normal conditions and at the final stages of repair processes. This assumption has been partially confirmed in some models of inflammatory processes [12,17,18].

In accordance with one of the much-discussed concepts, macrophages from different sources of origin differ not so much by the ability to produce pro- or anti-inflammatory cytokines but, above all, by sensitivity to various factors of the microenvironment. In particular, resident macrophages are more sensitive to the components of a normal microenvironment, which allows them to regulate homeostasis in organs under normal conditions, whereas the monocyte-derived macrophages are more sensitive to mediators of inflammation [14,16,19]. This concept is consistent with studies performed on the model of toxic liver damage, when a large number of monocyte-derived macrophages migrate into the liver. However, the effect is highly transient, as immigrant cells become replaced with the proliferating resident Kupffer cells at the final stages of repair [17]. Additionally, as has been previously shown by us in vitro, the monocytic macrophages are more sensitive to interleukins (IL4, IL10) and notably to lipopolysaccharide than Kupffer cells [20]. The increased sensitivity of monocytic macrophages to lipopolysaccharides is consistent with the data on gene expression obtained by nanostring and RT-PCR assays in this study. According to the results, monocytes show upregulated expression of *Tlr 4*. At the same time, some experimental studies indicate the lack of advantages in the regulation of the normal functions of organs by resident macrophages as compared with macrophages of bone marrow origin; this conclusion was made by a team of authors who completely replaced the resident macrophages in the liver and lung with macrophages of bone marrow (monocytic) origin for a long period of time [11,14]. Therefore, it is probable that the determining factors for the functional characteristics of macrophages are not the source of their origin but the factors of the microenvironment [14,16,21].

Based on the ability of bone marrow-derived macrophages to make up for the loss of resident macrophages and replace them in the corresponding macrophage niche, the key step in this process is migration to the organ of destination, which occurs at the intermediate stage of monocyte-macrophage differentiation [8].

Blood monocytes of many mammals consist of several cell subpopulations. In the blood of mice, the monocyte population consists of two subpopulations (Ly6C^hi^ and Ly6C^low^ or Ly6C^+^ and Ly6C^−^), and the ratio of such subpopulations is approximately 1:1 [22]. We found that approximately 50% of the isolated monocytes were Ly6C^+^. Probably, the proportion of Ly6C^+^ (approximately 50%) corresponds to a Ly6C^hi^ subpopulation of monocytes, which, as suggested, migrate to the liver under inflammation conditions [17] and have pro-inflammatory properties [22,23,24].

Monocyte subpopulations represent a single line of differentiation; Ly6C^hi^ monocytes differentiate into Ly6C^low^ monocytes through an intermediate stage [25]. In this regard, the Ly6C^hi^ and Ly6C^low^ subpopulations have a number of similarities, both at the molecular [26] and functional level; they are highly sensitive to TLR stimuli [23,27], can migrate into tissues, and also secrete pro-inflammatory cytokines TNFa and Il1b [24,25].

Our data are also consistent with the results of the mentioned studies. The migrating cells must be highly sensitive to the organ-specific characteristics of the microenvironment. The most pronounced migration of monocytes/macrophages of bone marrow origin to the liver can be observed under conditions of hepatotoxic damage [17]. Thus, monocytes must be highly sensitive to pathogen-associated molecular patterns (PAMPs). This assumption is consistent with the increased expression of *Tlr2*, *Tlr4*, *Tlr7*, and *Tlr8* genes in monocytes, as well as the high levels of cell locomotion and cell adhesion proteins synthesis (*Ccl24*, *Ccr1*, *Cxcl2*, *Cxcl1*, *Itgb2*, *Ccl17*, *Ccl2*), observed by us in this study. The protein products (codenamed P30681) of the overexpressed *Itgb2* gene (Integrin beta-2) were significantly overrepresented in monocytes. Expression of many markers associated with particular functions of macrophages (endocytosis, antigen presentation, pathogen recognition, lymphocyte activation), including CD68, CD86, CD163, and CD206 [28,29], in monocytes is lower than in the resident macrophages of the liver and indicates the position of monocytes as precursors, which retain the high plasticity.

However, along with the signs of a low-differentiated state, monocytes, according to the results of nanostring assay and proteome analysis presented in this study, harbour increased expression of specific targets characteristic of differentiated macrophages, including several chemokines and chemokine receptor genes, the complement system protein-encoding genes, lipoxygenase genes, and pro-inflammatory cytokine genes (*Il1a*, *Il6*, *Il12a*). Thus, the obtained results are consistent with the consideration of monocytes as highly specialized blood macrophage cells [24,30].

The upregulated levels of transcription observed in Kupffer cells for a particular set of genes reflect the involvement of these genes in maintaining normal liver homeostasis. The relevant metabolic pathways include specific patterns of microcirculatory blood flow regulation, characteristic of hepatic microenvironments (*iNOs, Flt1*, *Kng1*), as well as liver-specific biochemical cascades (*Arg1 C2*, *C6*, *C9*, *Crp*, *Retnla*). The potential role of resident macrophages as an accessory cell type for performing basic organ functions, especially in the liver, is illustrated by high expression of specific surface markers, e.g., CD163 and CD206 (reportedly involved in haemoglobin utilization and hormone metabolism, respectively) in Kupffer cells [28,29].

Notably, by studying the expression profile using nanostring in Kupffer cells obtained from different animals, some heterogeneity in the expression of several genes was revealed. At the same time, Kupffer cells had a similar immunophenotype, and most of the genes expressed in the group were homogenous. Presumably, different expression levels of the same genes reflect the functional and phenotypic heterogeneity of macrophages in general and Kupffer cells in particular [31].

The data obtained by the proteomic analysis were consistent with these observations. Kupffer cells were found to be more oriented to metabolic processes, such as transport, lipid metabolism, and energy metabolism compared to monocytes. In addition, the Kupffer cell proteome is enriched with proteins associated with mitochondria and energy metabolism. It is well known that energy metabolism is closely related to the activity of macrophages. Increased level of oxidative phosphorylation is characteristic of the energy metabolism of M2 macrophages, while M1 macrophages show increased levels of glycolysis and reduced oxygen consumption rates [32,33].

miRNAs play a significant role in the regulation of intracellular processes and control gene expression at the post-transcriptional level [34]. However, the number of studies about the role of miRNAs in the regulation of macrophage activity is limited. We firstly identify high levels of expression of liver-specific miR-122 in resident macrophages of the liver. High levels of expression of miR-122 in hepatocytes [35], as well as its key role in maintaining liver homeostasis and its anti-inflammatory activity [36,37], were demonstrated previously by other authors. Data on the role of other miRNAs in the regulation of macrophage activity are contradictory [38,39]. However, a number of studies show that let-7 family miRNAs, such as let-7b/c/d/e, contributed to the formation of an anti-inflammatory (alternative) macrophage phenotype [40,41]. According to the results of the current study, let-7b/c/d/e miRNAs were expressed at a higher level in Kupffer cells. We also found increased expression of miR-1224 miRNA, which is involved in the regulation of HGF synthesis, in resident macrophages of the liver [42]. As for the remaining miRNAs, the increased expression of which we observed in Kupffer cells, their roles in the regulation of macrophage activity remain unknown and needs further research. Bioinformatics analysis shows that some of these genes may be involved in cytokine signalling and TNFa cascade.

Thus, the profile of microRNAs expressed by resident macrophages of the liver partially reflects their organ specificity and participation in both the maintenance of liver homeostasis (high expression of miR-122) and anti-inflammatory functional activity (high expression of let-7b/c/d /e).

The phenomenon of polarization of macrophages towards the regulation of homeostasis of organs under varying conditions linked the life-long coexistence of the two macrophage lineages in mammals with the M1/M2 paradigm of macrophage function [43]. The initially introduced model of the alternatively polarized macrophage functional types has undergone substantive changes. Polar M1 and M2 phenotypes are currently viewed as extreme points of the continuous spectrum of functional types of macrophages. Accordingly, the search for specific markers of the M1 or M2 phenotype should be fine-tuned and updated [3].

Analysis of data obtained in this study shows that Kupffer cells of the liver and blood monocytes substantially differ in gene expression profiles; however, the observed differences do not strictly correspond to the M1/M2 paradigm. For instance, it is accepted that the M1 phenotype is specifically marked by high expression levels of *iNOs* and *STAT1*, whereas the M2-phenotype is typically accompanied by upregulated expression of certain chemokines (*Ccl17*, *Ccl24*, *Retnla*)*,* as well as *Arg1*, *STAT3*, and *STAT6* [2]. By applying the recently developed nanostring approach we demonstrated that, in comparison to blood monocytes, Kupffer cells have significantly higher levels of expression for both *Arg1* and *iNOs*; this finding was confirmed by RT-PCR and western blot analysis for the corresponding proteins. At the same time, *Ccl17* and *Ccl24* transcription was more intense in monocytes, and no significant differences in expression between monocytes and Kupffer cells were observed for *STAT1* and *STAT3*. Therefore, it would be incorrect to decisively assign monocytes and Kupffer cells to M1 and M2 phenotypes, respectively.

## 4. Materials and Methods

The animals were anesthetized with ether, the livers were perfused with phosphate buffered saline (PBS) via a vena porta, removed, and minced with scissors. The cells were isolated by enzymatic digestion with collagenases type I and IV (PanEco, Moscow, Russia) at 37 °C for 30 min, filtered through a 100 µm nylon filter (SPL Life Science, Geumgang-ro, Korea), and washed twice. The resulting cell pellets were resuspended in 30 mL PBS and centrifuged at 50× *g* for 3 min; parenchymal cells of the liver were collected in the pellet, whereas non-parenchymal cells remained in the supernatant, which was subjected to gradient centrifugation on Lympholyte-M (Cedarlane, Burlington, ON, Canada) at 1200× *g* for 20 min at room temperature yielding a fraction predominantly composed of Kupffer cells. The cells subsequently underwent immunomagnetic sorting on a manual MidiMACS™ Separator by using LS Columns (Miltenyi Biotec, Bergisch Gladbach, Germany) and magnetic beads (Anti-F4/80 MicroBeadsUltraPure; Miltenyi Biotec, Bergisch Gladbach, Germany) in accordance with the manufacturer’s protocols. For each sample, Kupffer cells from 5 mice were pooled.

### 4.1. Isolation of Kupffer Cells

The animals were anesthetized with ether, the livers were perfused with phosphate buffered saline (PBS) via vena porta, removed and minced with scissors. The cells were isolated by enzymatic digestion with collagenases type I and IV (PanEco, Moscow, Russia) at 37 °C for 30 min, filtered through a 100 µm nylon filter (SPL Life Science, Geumgang-ro, Korea) and washed twice. The resulting cell pellets were resuspended 30 mL of PBS and centrifuged at 50× *g* for 3 min: parenchymal cells of the liver were collected in the pellet whereas non-parenchymal cells remained in the supernatant, which was subjected to gradient centrifugation on Lympholyte-M (Cedarlane, Burlington, ON, Canada) at 1200× *g* for 20 min (room temperature) yielding a fraction predominantly composed of Kupffer cells. The cells subsequently underwent immunomagnetic sorting on a manual MidiMACS™ Separator by using LS Columns (Miltenyi Biotec, Bergisch Gladbach, Germany) and magnetic beads Anti-F4/80 MicroBeadsUltraPure (Miltenyi Biotec, Bergisch Gladbach, Germany) in accordance with the manufacturer’s protocols. For each sample, Kupffer cells from 5 mice were pooled.

### 4.2. Isolation of Monocytes

The monocytes isolation procedure was based on magnetic sorting with antibodies to F4/80 [44]. Blood was collected from mice, mixed in a 1:1 proportion with Hank’s Balanced Salt Solution containing 1000 ME/mL of heparin, and separated by gradient centrifugation using Ficoll (PanEco, Moscow, Russia) at 400× *g* for 30 min at 4 °C. The fraction of mononuclear cells was collected and washed twice. The cells underwent immunomagnetic sorting on a manual MidiMACS™ Separator by using LS Columns (Miltenyi Biotec, Bergisch Gladbach, Germany) and magnetic beads (Anti-F4/80 MicroBeadsUltraPure; Miltenyi Biotec, Bergisch Gladbach, Germany) in accordance with the manufacturer’s protocols. For each sample, monocytes from 5 mice were pooled.

### 4.3. Flow Cytometry Assay

For flow cytometry sampling, sorted cells from 5 animals were pooled. For intracellular marker staining, the cells were processed using InsideStainKit (Miltenyi Biotec, Bergisch Gladbach, Germany) in accordance with the manufacturer’s recommendations. For surface immunophenotype marker staining, the cells were resuspended in PBS (1 × 10^5^ cells in 100 µL). Antibodies with the corresponding serotype controls are listed in Table 1. Analysis was performed on a Cytomics FC 500 flow cytometer (BeckmanCoulter, Indianapolis, IN, USA) with the CXP program (Beckman Coulter, Indianapolis, IN, USA). The sorted cells were gated based on their forward and side scatter properties. The main cell population was placed in the region of interest, excluding debris. 

Immunophenotyping was accomplished by gating the main cellular pool after magnetic sorting (see the FS vs. SS dot plots; Figure 1A,B with similar region of interests for monocytes and Kupffer cells) and subsequent staining for the relevant markers (Ly6/C, CD68, CD163, CD86, CD206, CD86, CD11b, and F4/80). The flow cytometry assay was originally focused on the proportions of positive monocytes as compared with Kupffer cells for each of the studied markers. The proportion of negative cells was determined in relation to the isotype controls, performed for each sample. Quadrant axes on dot-plots were also set according to isotype controls.

### 4.4. Immunocytochemistry Assay

The expression of various markers was estimated by overnight staining of monocytes and macrophages with antibodies to CD68, CD86, CD206 (1:100, Abcam, Bristol, UK), CD11b, and CD163 (1:100, Santa Cruz, Dallas, TX, USA). Thereafter, cells were washed thrice and incubated with secondary antibodies conjugated with FITC (1:200, Abcam) for 1 h, the nuclei were counterstained with 4′,6-diamidino-2-phenylindole (DAPI) (Sigma-Aldrich, Darmstadt, Germany) as described previously [45]. Immunostaining analysis was performed with a Leica DM 4000 B fluorescent microscope and LAS AF software (Leica Microsystems, Wetzlar, Germany).

### 4.5. Nanostring Gene Expression Assay and Target Analysis

For the nanostring gene expression assay sampling, sorted cells from 4 to 5 animals were pooled. The RNA was isolated from cells by using RNeasy Plus Mini Kit (Qiagen, Hilden, Germany) with additional purification and concentration with RNA Clean& Concentrator (Zymo Research Irvine, CA, USA).

Nanostring technology allows one to receive a signal from a sample, not relative to another—for example, with array CGH (also put on chips, but external control is required), or with sequencing (mapped to a ref genome), but simply measures the fluorescent signal and unambiguously links its magnitude with a specific target gene. The manufacturer provides eight NEG and six POS controls on the chip. The manufacturer does not disclose in detail and does not describe what it uses as these controls. The panel we use nCounter_Mouse_Inflammation_V2_Panel also contains 6 genes (Cltc, Gapdh, Gusb, Hprt, Pgk1, Tubb5), the data from which are used for normalization of results. We started by evaluating the general assay performance using Quality Control (QC) metrics recorded in the nCounter data files (RCC files). Afterward, we performed background correction and data normalization followed by another round of data QC. Finally, we assessed the resulting ratios, fold-changes and differential expression. First, all QC metrics were checked: Imaging QC, Binding Density QC, Positive Control Linearity, Limit of Detection. The last two were calculated based on the POS control data. NSolver Analysis Software 4.0, when processing our data, confirmed that all QCs were within the manufacturer’s recommended values. Next, we set the background threshold to the max of NEG Control.

Standard normalization uses a combination of Positive Control Normalization, which uses synthetic positive control targets, and CodeSet Content Normalization, which uses housekeeping genes, to apply a sample-specific correction factor to all the target probes within that sample lane. In both cases, geometric mean normalization was chosen. Next, we grouped our data into piles, six samples of monocytes were assigned to one group, six samples of Kupffer cells, respectively, to the second. Further, we grouped our data as follows: six samples of monocytes (MNCs) were assigned to one group, six samples of Kupffer cells (KCs), respectively, to the second. nSolver Analysis Software 4.0 gave us fold-change and p-value. Next, we selected those targets whose FC is higher than 2 or less than −2. There were 84 of them (Table S4). In Table S4, we show the FC values of KCs vs MNCs, thus positive values-expression is higher in KCs, negative expression is higher in MNCs.

To confirm the nanostring data, 12 genes were selected, the expression of which was studied using Real-Time Polymerase Chain Reaction (RT-PCR), these data are presented in Figure 2B. Indeed, it is not clear from the heatmaps that the expression of the complement protein genes differed by more than 15 times, therefore, we provide the table of the nanostring data with differential genes expression (please see Table S4), on the basis of which the heatmaps were depicted. The inflammation-related gene expression and miRNA expression were measured in an nCounter FLEX Analysis System using an nCounter Mouse Inflammation V2 panel and Mouse miRNA V 1.5 panel.

The data were processed in nSolver Analysis Software 4.0. MiRNA target prediction was based on Targetscan [46,47], miRNet [48] using KEGG for clustering analysis.

### 4.6. Real-Time Polymerase Chain Reaction (RT PCR) Gene Expression Assay

To confirm the nanostring data 12 mRNAs and 7 miRNAs were selected. Total RNA was isolated from cells using an RNeasy Plus Mini Kit (Qiagen, Hilden, Germany); the corresponding cDNA was synthesized by using an MMLV RT kit (Evrogen, Moscow, Russia). The polymerase chain reactions were set in duplicates with qPCRmix-HS SYBR master mixes containing Sybr Green I dye (Evrogen, Moscow, Russia). The target-specific primers (Table 2) were designed by using the Primer-BLAST tool (NCBI, USA) in accordance with general rules and custom ordered from Evrogen. For real-time PCR, we used primers that anneal close to or in the same place as recognized by nanostring probes.

To analyse miRNA expression, reverse transcription was performed using a miScript II RT Kit (Qiagen, Hilden, Germany). For RT-PCR, a miScript SYBR^®^ Green PCR Kit (Qiagen, Hilden, Germany) was used with the miScript Universal Primer as the reverse primer. Direct primers used in the work are shown in Table 3.

The cycler DT Prime M1 (DNA technology, Moscow, Russia) was used in the work. The expression was quantified by the threshold cycle (Ct) approach; the relative expression levels were evaluated via the algorithm proposed by M. Pfaffl [49] with *Gapdh* (in case of mRNA) and RNU6 (in case of miRNA) as a housekeeping reference target according to the formula: Gene expression ratio= (E_target_)^^C^_t_^,target^/(E_ref_)^^C^_t_^,ref^, E target and E ref are the amplification efficiencies of the target and reference gene, respectively.

### 4.7. Proteome Analysis

Samples preparation was carried out according to the previously described method [50]. MS data were collected in DDA mode. MS1 parameters were as follows: 140K resolution, 350–2000 scan range, max injection time of 35 ms, and AGC target 3 × 106. Ions were isolated with a 1.4 m/z window with 0.2 m/z offset targeting of the 10 highest intensity peaks of +1 to +6 charge and of 8x103 minimum AGC. Dynamic exclusion was set to 40 s. MS2 fragmentation was carried out in CID mode at 17.5 resolution with 28% NCE. Ions were accumulated for a max of 50 ms with target AGC 1 × 10^5^.

Raw spectra were processed using MaxQuant 1.6.6.0 (MQ) [51] and Perseus [52]. The data were searched against the Mus Musculus Uniprot database (contains 84,951 entries, including both canonical and isoforms, downloaded 23.03.18).

The MaxQuant search was performed with the default parameter set, including Trypsin/p protease specificity, max two missed cleavages, Met oxidation, and Protein N-term acetylation as variable modifications and Carbamidomethyl Cys as a fixed modification, max five modifications per peptide, 1% PSM, and protein FDR. The following options were turned on: second peptide, maxLFQ, and match between runs. All runs were analysed as independent experiments and processed in Perseus.

In Perseus, the protein group results were filtered for contaminants, reverse, and ‘identified only by site’ proteins. Only the proteins with maxLFQ values of at least 3 out of 7 LC-MS runs were used. For them, missing values were imputed from a normal distribution with 0.3 intensity distribution sigma width and 1.8 intensity distribution center downshift. A two-sample *t*-test with a permutation-based FDR of 5% was applied to search for significantly changing proteins.

Enrichment analysis was performed in DAVID [53,54] and PANTHER [55]. To correlate gene expression data from nanostring gene expression panel with proteome analysis data, we used a db2db instrument from the bioDBnet site [56]. We transformed gene symbols and protein UniProtKB IDs to the ensemble ID format and compared matched gene expression and proteins abundance in Kupffer cells and monocytes.

Correlation of miRNA targets and proteome analysis was performed using the db2db instrument. In this case miRNA targets were identified by TargetScan software with a threshold value of -1.0 with a cumulative weighted context++ score (the context++ score for a specific site is the sum of the contribution of a series of features: site type, supplementary pairing, and minimum distance, among others). For each predicted target of each miRNA, the sum of the context++ scores for the sites to that miRNA was calculated as the total context+ score.

### 4.8. Western-Blot Analysis of Protein Representation

For Western-Blot sampling, sorted cells from 5 animals were pooled. The cell pellet was lysed with 50 μL of ice-cold RIPA buffer. Thereafter, Laemmli Sample Buffer (Bio-Rad Laboratories, Inc., Hercules, CA, USA) was added with heating at 95 °C. The proteins were separated by 10%–12.5% sodium dodecyl sulphate polyacrylamide gel electrophoresis (SDS-PAGE) and transferred to a membrane as described previously [57]. After blocking membranes were stained overnight with antibodies to Arg1, iNOs, CCR7, IL6, IL10, FXYD2, ALOX12, or GAPDH (anti-Arginase I: sc-18351, 1:200; anti-NOS2:sc-651, anti-GAPDH:sc-25778, Santa Cruz, USA, anti-CCR7:ab32527, anti-IL6:ab7737, anti-IL10:ab9969, Abcam, UK), anti-FXYD2:PA5-75640, anti-ALOX12:PA5-78760, ThermoScientific, Waltham, MA, USA), washed thrice, and stained with HRP-conjugated antibodies (Bio-Rad Laboratories, Inc., Hercules, CA, USA). Bands were imaged with a Novex ECL Kit (Invitrogen, Carlsbad, CA, USA) in a ChemiDoc™ system (Bio-Rad Laboratories, Inc., Hercules, CA, USA). Densitometry analysis was carried out using ImageLab Software (Bio-Rad Laboratories, Inc., Hercules, CA, USA). GAPDH was used as a reference protein. Uncropped membranes after blotting are available in Supplementary Materials.

### 4.9. Statistical Analysis

The data were analysed by using SigmaStat 3.5 software (Systat Software Inc., San Jose, CA, USA); the relative expression levels, immunophenotype indicators, and Western-Blot densitometry for monocytes and Kupffer cells were compared by a Mann–Whitney test at the *p* ˂ 0.05 level of significance.

## 5. Conclusions

Comparison of murine blood monocytes with Kupffer cells of the liver revealed considerable differences in gene expression profiles. Conversely, these differences reflect the specific position of monocytes as an intermediate migratory cell stage in the macrophage lineage. At the proteome level, monocytes expressed a variety of proteins associated with cell adhesion, high sensitivity to the microenvironment factors, and pathogen-associated molecular patterns (PAMPs) (increased expression of *Tlr4*, *Tlr8*, and *Tlr9*), as well as the capability of subsequent differentiation (cell cycle and cell division regulatory proteins). In contrast, Kupffer cells revealed a signature more typical of mature resident macrophages. Profiles of gene expression, including microRNA genes, and protein expression in Kupffer cells reflect the involvement of these cells in the regulation of specific functions of the liver, including nitrogen metabolism, lipid metabolism, and complement system protein synthesis.

## Figures and Tables

**Table 1:** Antibodies used in the flow cytometry assay.

Antibody	Isotypic Control	Manufacturer
CD11b-VioBright FITC, mouse (clone: REA592)	REA Control-VioBright FITC	Miltenyi Biotec
CD86-PE-Vio770, mouse	Rat IgG2b-PE-Vio770	Miltenyi Biotec
Ly-6C-PE, mouse	Rat IgG2a-PE	Miltenyi Biotec
CD206 (MMR) Monoclonal Antibody (MR6F3), PE	Rat IgG2b kappa Isotype Control (eB149/10H5), PE	eBioscience™
CD163-PE (Thermo Fisher) Monoclonal Antibody	Mouse IgG1 kappa Isotype Control, PE, eBioscience™	eBioscience™
Anti-F4/80-PerCP-Vio700, mouse	REA Control-PerCP-Vio700	Miltenyi Biotec
CD68-FITC, mouse (clone: FA-11)	Rat IgG2a-FITC	Miltenyi Biotec

**Table 2:** Primers for polymerase chain reaction (PCR).

*FIZZ1 (Retnla)*	for	CCTTTCCTGAGATTCTGCCCC
	rev	CGAGTAAGCACAGGCAGTTG
*TGFb2*	for	TCCCCTCCGAAAATGCCATC
	rev	CCTCCGCTCTGGTTTTCACA
*Alox15*	for	AAGATGTAACCCACCACGTTCA
	rev	TGCCCCGATGACACAGAAAA
*Tlr8*	for	TGGTCCAGCTATAGAGCACATC
	rev	ACTGAGGGGGCATGTTTTCC
*Flt1*	for	ACAAGTCAAACCTGGAGCTGA
	rev	TGCAGAGGCTTGAACGACTT
*IL1r1*	for	GACCCCCATATCAGCGGACC
	rev	ACCGGATATTGCTTCCCCCG
*Crp*	for	GACTCGTATGGCGGTGACTT
	rev	GCTGAGTGTCCCACCAACAT
*Kng1*	for	GTGAAGCAGTAGTCCCAGCAA
	rev	CCCTCTAATCATCCCTGTGGC
*C9*	for	CCCTTGCCATCTTTGCCTTG
	rev	GTGGGTCATCGTCACAGTCA
*Il6*	for	CCACTTCACAAGTCGGAGGC
	rev	GGAGAGCATTGGAAATTGGGGT
*Arg1*	for	ACGGCAGTGGCTTTAACCTT
	rev	AGGTAGTCAGTCCCTGGCTT
*NOS2*	for	GCTGCCAGGGTCACAACTT
	rev	CCTCACATACTGTGGACGGG
*GAPDH*	for	AGGCCGGTGCTGAGTATGTC
	rev	TGCCTGCTTCACCACCTTCT

**Table 3:** Primers for microRNA PCR.

mmu-miR122-for	TGGAGTGTGACAATGGTGTTTG
mmu-let-7c-for	TGAGGTAGTAGGTTGTATGGTT
mmu-let-7d-for	AGAGGTAGTAGGTTGCATAGTT
mmu-miR-720-for	ATCTCGCTGGGGCCTCCA
mmu-miR-1224-for	GTGAGGACTGGGGAGGTGGAG
mmu-miR-1944-for	CTCTGTGCTGAATGTCAAGTTCTGATT
mmu-miR-2141-for	AGGAGGTGTCAGAAAAGTT
RNU6-for	CTCGCTTCGGCAGCACA

## Data Availability

The original contributions presented in the study are included in the article/supplementary files; further inquiries can be directed to the corresponding author/s. The data reported in this paper have been deposited in the Gene Expression Omnibus (GEO) database, https://www.ncbi.nlm.nih.gov/geo (accession no. GSE150878, GSE150808). The mass spectrometry proteomics data have been deposited to the ProteomeXchange Consortium via the PRIDE [58] partner repository with the dataset identifier PXD018586.

## Supplementary Materials

The following are available online at https://www.mdpi.com/2227-9059/8/12/627/s1. Figure S1. Flow cytometry analysis of unsorted blood samples. Representative forward and side scattering, histogram, and dot-plot are shown. The proportions of negative cells were determined in relation to the isotype controls. The percentages of positive cells are indicated. The blue curve corresponds to the isotype control. Figure S2. Flow cytometry analysis of unsorted liver stromal cells. Representative forward and side scattering, histogram, and dot-plot are shown. The proportions of negative cells were determined in relation to the isotype controls. The percentages of positive cells are indicated. The blue curve corresponds to the isotype control. Figure S3. MiRNAs expression analysis in monocytes and Kupffer cells. Full-length heatmaps are presented. Figure S4: Pie charts of the proteome from monocytes and Kupffer cells are listed in molecular function and biological processes categories according to PANTHER (http://pantherdb.org/). Sectors of the category indicate proteins that are significantly up-regulated in Kupffer cells vs monocytes and vice-versa. Figure S5. Full-size membrane after blotting of polyacrylamide gel (A, B, C). Schemes of membrane Ponceau S staining and cutting of KC (Kupffer cells) and MNCs (monocytes samples) samples are shown. After visualization of the proteins with Ponceau S membranes were cut as indicated with a dotted line and stained with mentioned antibodies. On A and B—gels were run in parallel due to the similar molecular weight of Arg1, CCR7, and GAPDH. The green arrow indicates the detected band of interest. Figure S6. Uncropped membranes after blotting of polyacrylamide gel. Table S1. List of proteins up-regulated in Kupffer cells vs monocytes and belonged to PC00262 group of proteins (metabolite interconversion enzyme) according to PANTHER (http://pantherdb.org/). Table S2. List of proteins down-regulated in Kupffer cells vs monocytes and belonged to PC00171 group of proteins (nucleic acid binding protein) according to PANTHER (http://pantherdb.org/). Table S3. Highly correlated targets of the miRNAs with higher expression levels in Kupffer cells that are predicted by miRNet. Table S4. The list of mRNAs differs more than two times in Kupffer cells. Table S5. The list of miRNAs upregulated in Kupffer cells.
