# Supplementary material for: Comparative Analysis of the Transcriptome, Proteome, and miRNA Profile of Kupffer Cells and Monocytes
Source: Biomedicines. 2020 Dec 18;8(12):627. doi: 10.3390/biomedicines8120627 (PMC7766432; doi:10.3390/biomedicines8120627)
Supplement: Supplementary file 1 [file biomedicines-08-00627-s001.pdf]

Supplementary Materials

COMPARATIVE ANALYSIS OF THE TRANSCRIPTOME, PROTEOME AND miRNA PROFILE OF KUPFFER CELLS AND MONOCYTES

Andrey Elchaninov[1,3*,] Anastasiya Lokhonina[1,3], Maria Nikitina[2], Polina Vishnyakova[1,3], Andrey Makarov[1], Irina Arutyunyan[1], Anastasiya Poltavets[1], Evgeniya Kananykhina[2], Sergey Kovalchuk[4], Evgeny Karpulevich[5,6], Galina Bolshakova[2], Gennady Sukhikh[1], Timur Fatkhudinov[2,3]

[1] Laboratory of Regenerative Medicine, National Medical Research Center for Obstetrics, Gynecology and Perinatology Named after Academician V.I. Kulakov of Ministry of Healthcare of Russian Federation, Moscow, Russia
[2] Laboratory of Growth and Development, Scientific Research Institute of Human Morphology, Moscow, Russia
[3] Histology Department, Medical Institute, Peoples' Friendship University of Russia, Moscow, Russia
[4] Laboratory of Bioinformatic methods for Combinatorial Chemistry and Biology, Shemyakin-Ovchinnikov Institute of Bioorganic Chemistry of the Russian Academy of Sciences, Moscow, Russia
[5] Information Systems Department, Ivannikov Institute for System Programming of the Russian Academy of Sciences, Moscow, Russia
[6] Genome Engineering Laboratory, Moscow Institute of Physics and Technology, Dolgoprudny, Moscow Region, Russia

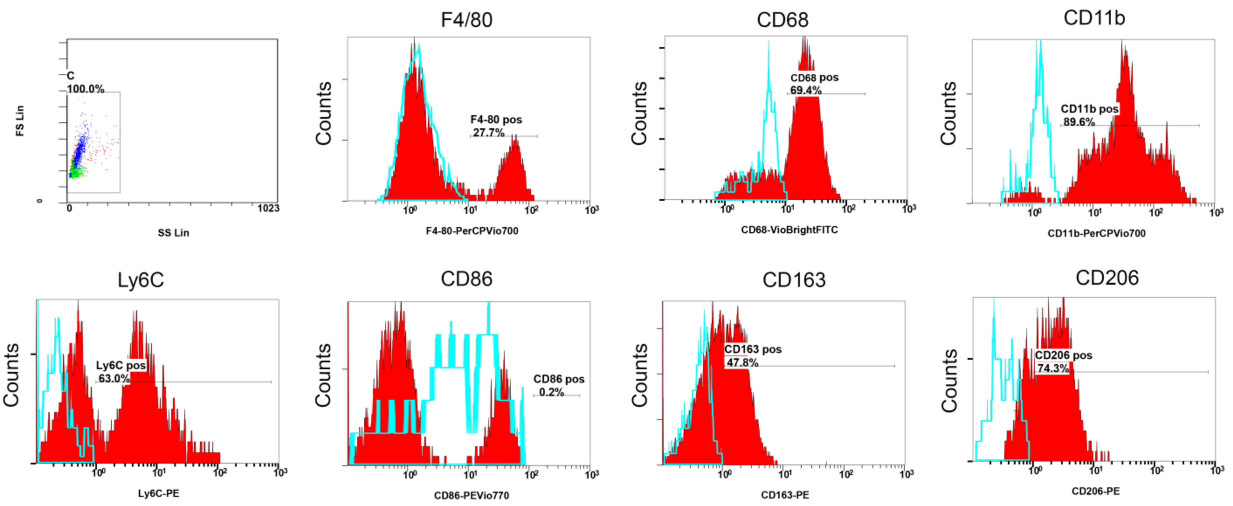

Figure S1. Flow cytometry analysis of unsorted blood sample. Representative forward, side scattering and histogram are shown. The proportions of negative cells were determined in relation to the isotype controls. The percentages of positive cells are indicated. The blue curve corresponds to the isotype control.

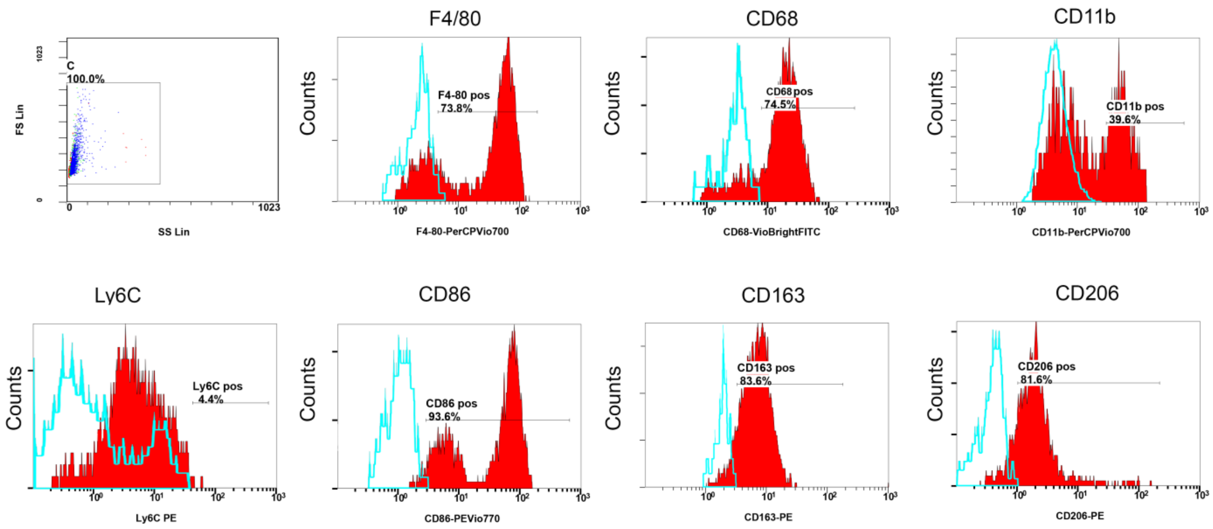

Figure S2. Flow cytometry analysis of unsorted liver stromal cells. Representative forward, side scattering and histogram are shown. The proportions of negative cells were determined in relation to the isotype controls. The percentages of positive cells are indicated. The blue curve corresponds to the isotype control.

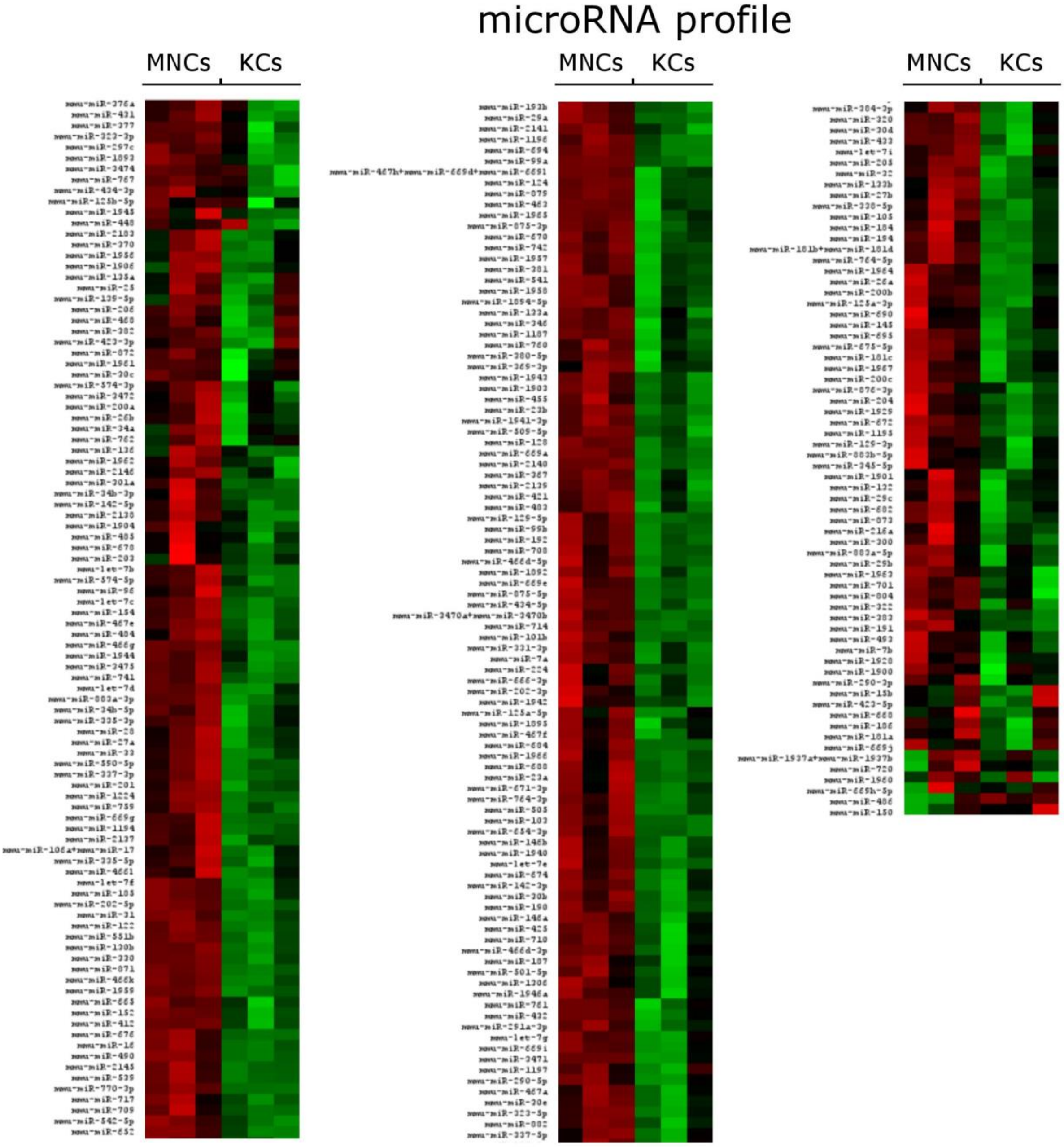

Figure S3. MiRNAs expression analysis in monocytes and Kupffer cells. Full-length of heatmaps are presented.

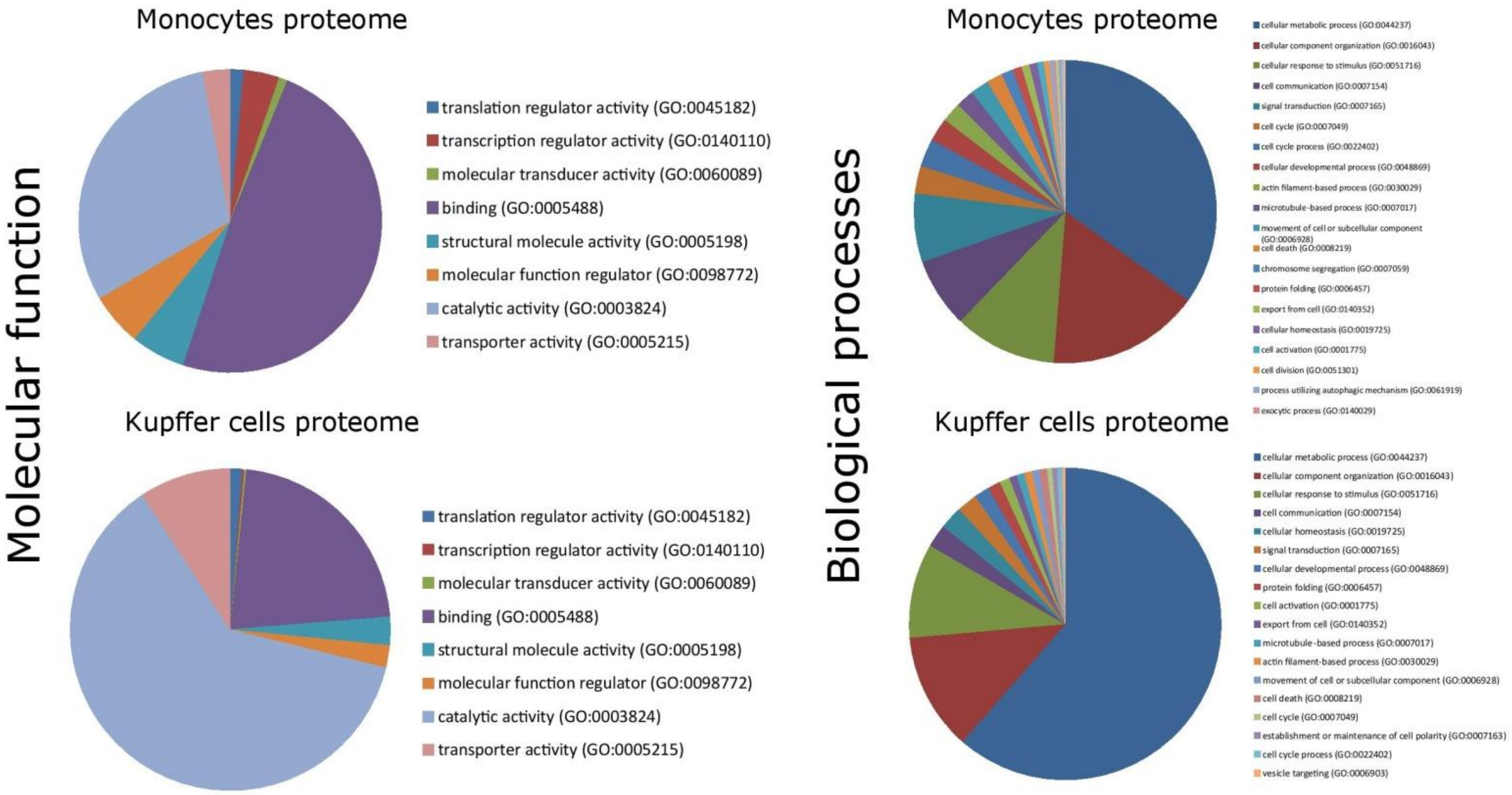

Figure S4: Pie charts of the proteome from monocytes and Kupffer cells are listed according to molecular function and biological processes categories according to PANTHER (http://pantherdb.org/). Sectors of the category indicate proteins that are significantly up-regulated in Kupffer cells vs monocytes and vice versa.

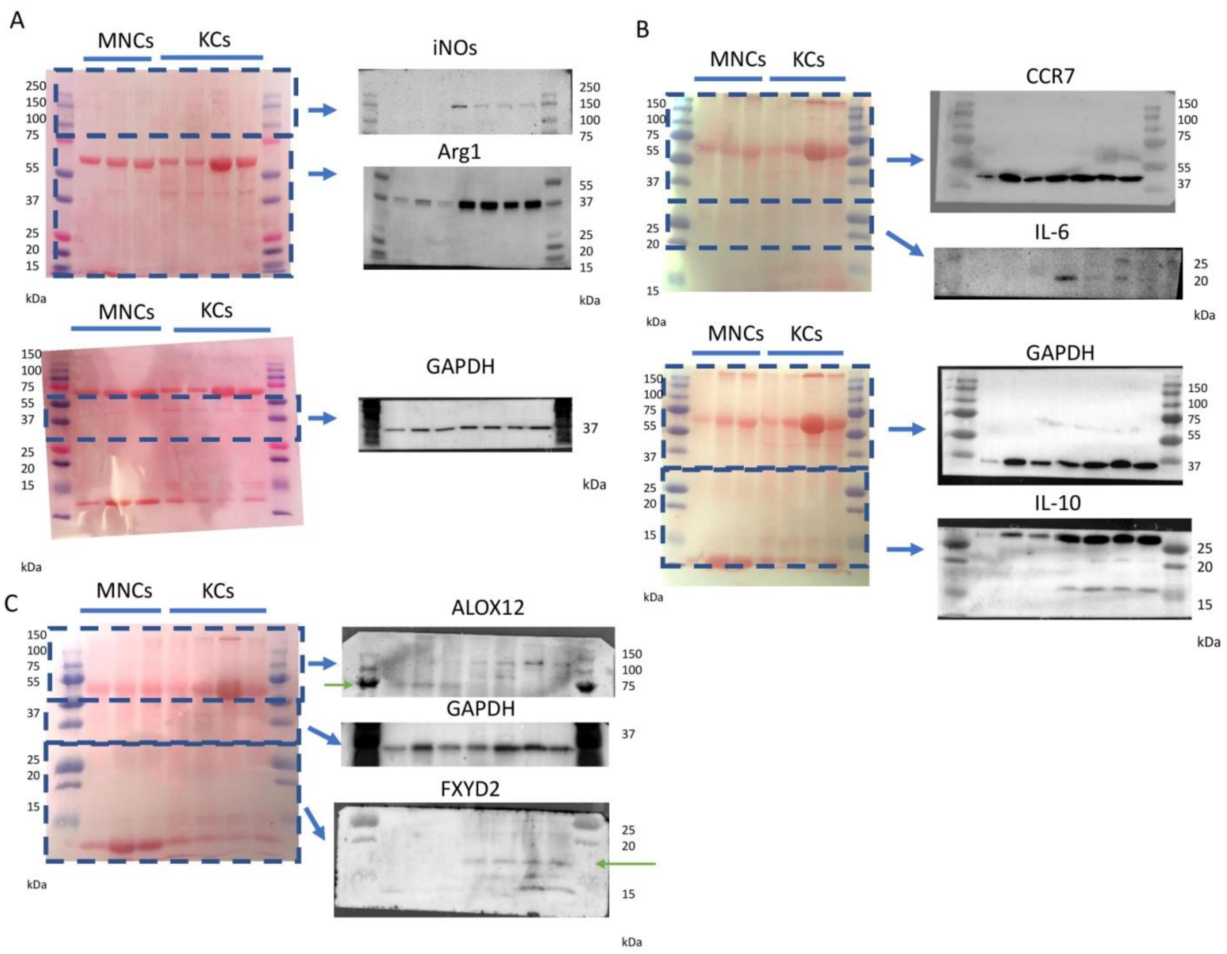

Figure S5. Full-size membrane after blotting of polyacrylamide gel (A, B, C). Schemes of membrane Ponceau S staining and cutting of KC (Kupffer cells) and MNCs (monocytes samples) samples are shown. After visualization of the proteins with Ponceau S membranes were cut as indicated with a dotted line and stained with mentioned antibodies. On A and B – gels were run in parallel due to the similar molecular weight of Arg1, CCR7 and GAPDH. Green arrow indicates the detected band of interest.

ALOX2

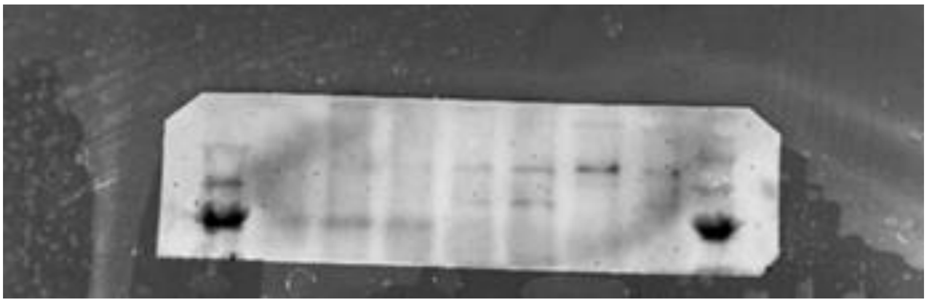

Arginase1

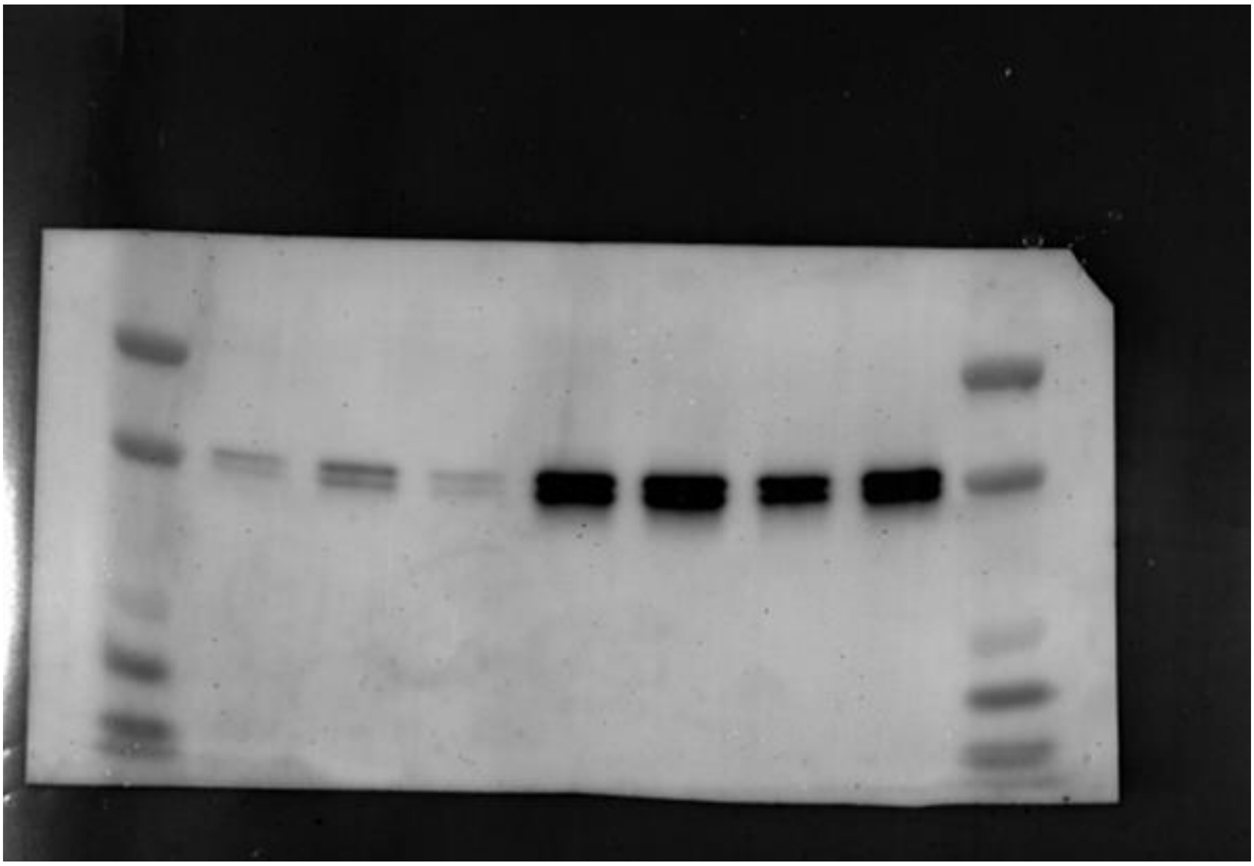

CCR7

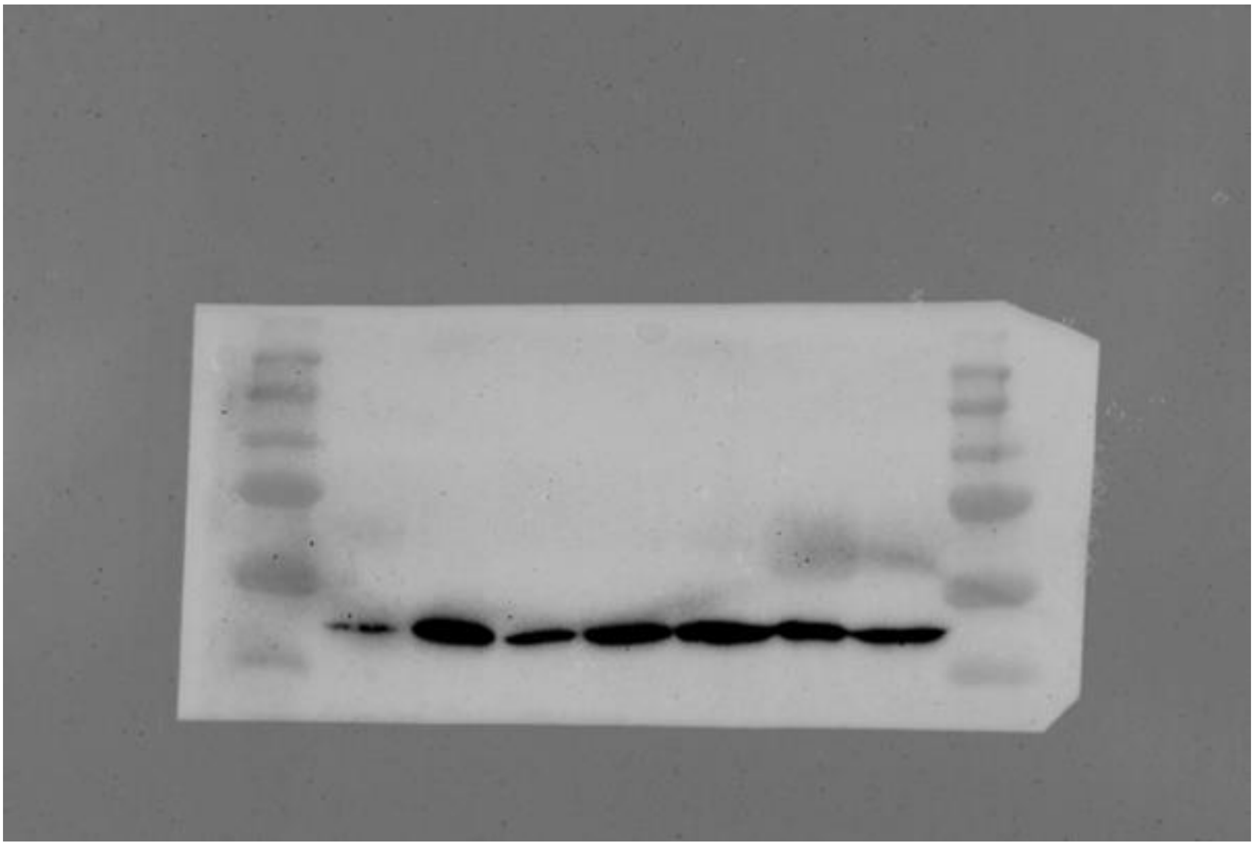

FXYD2

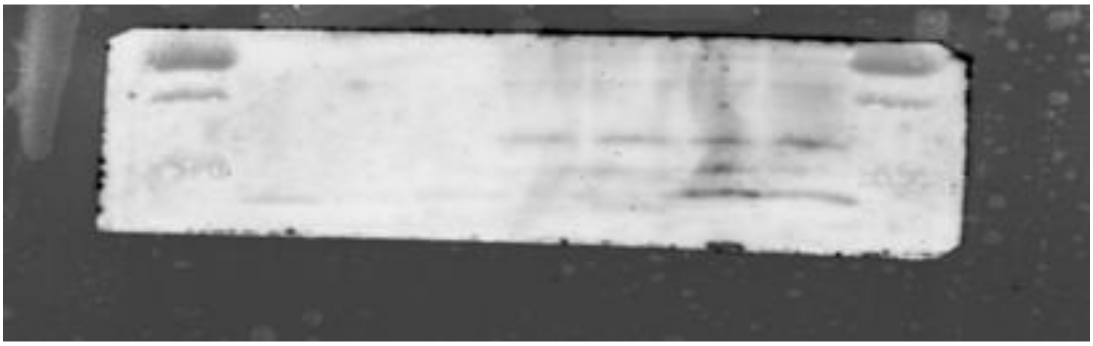

IL6

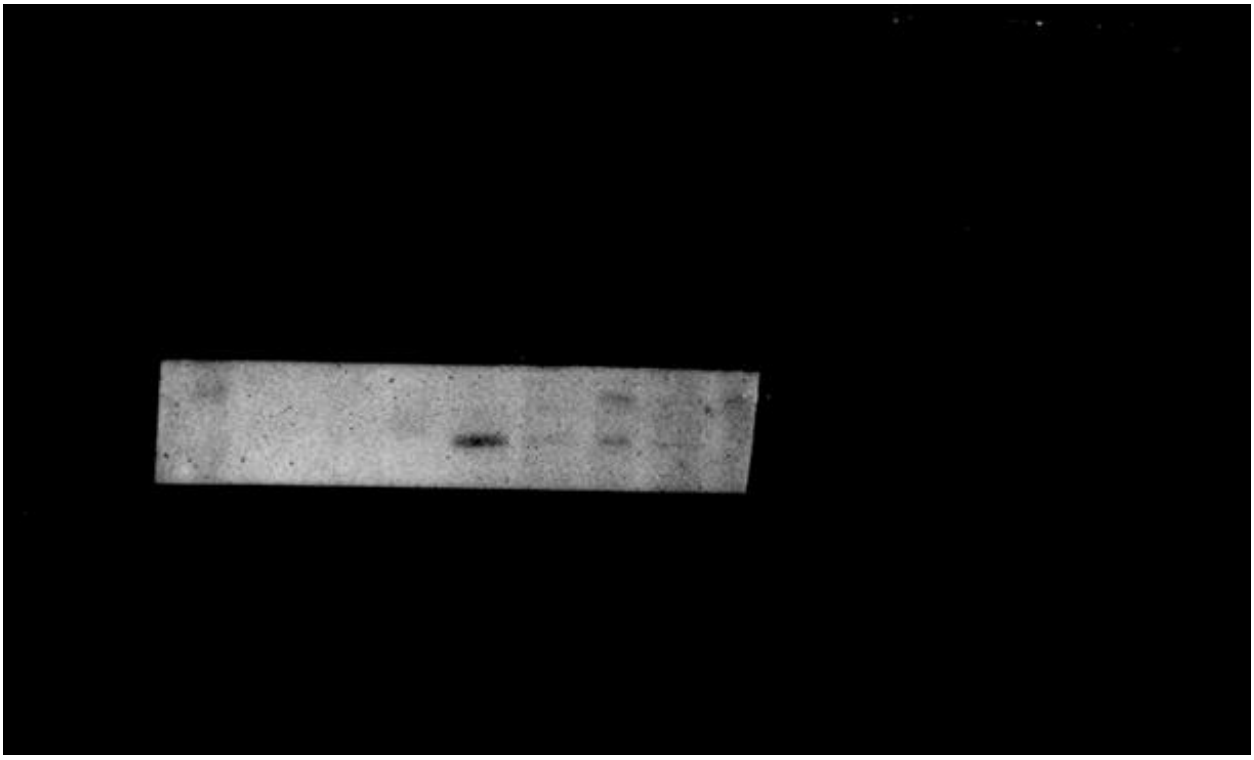

IL10

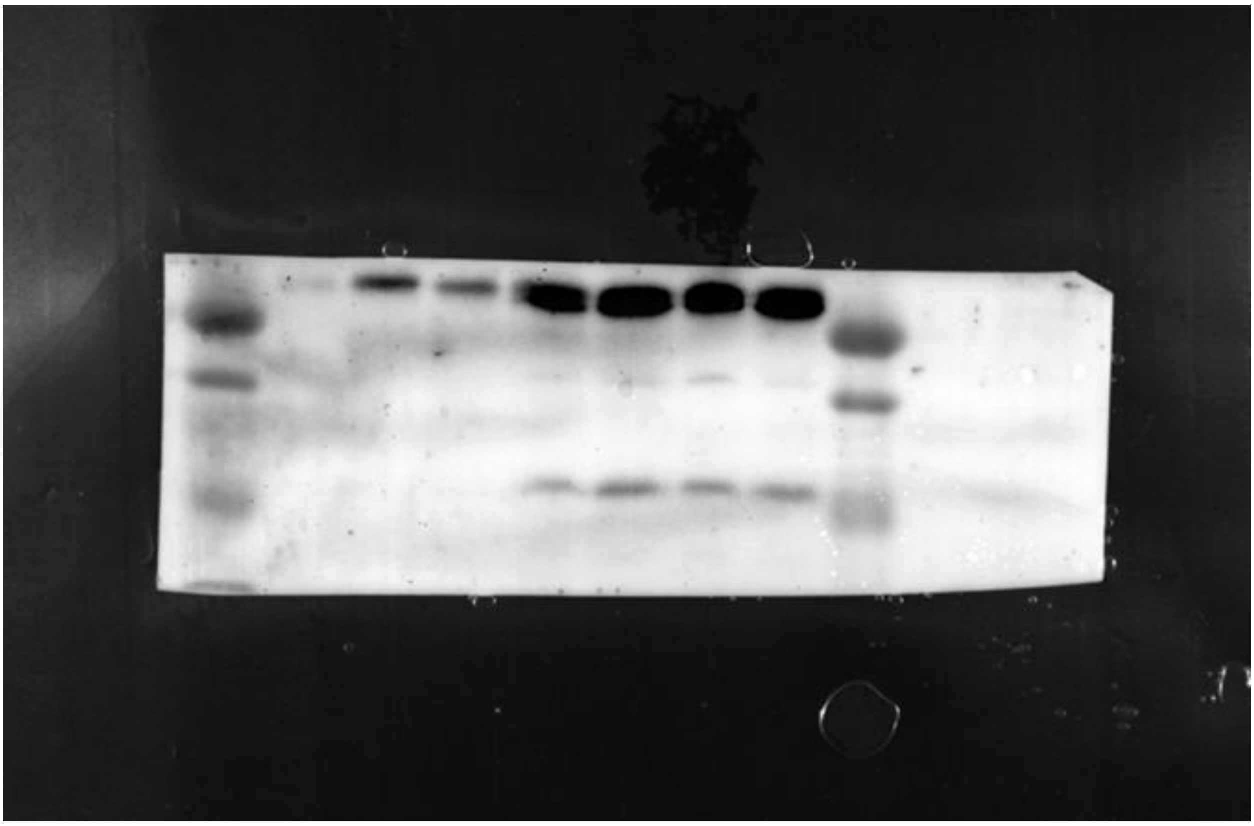

NOS2

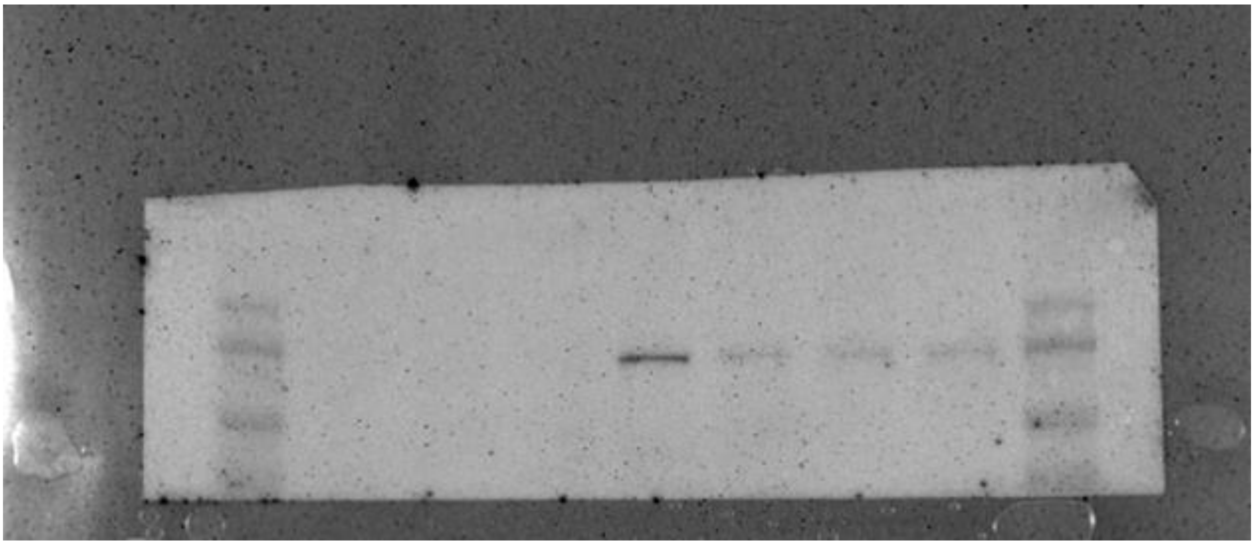

GAPDH

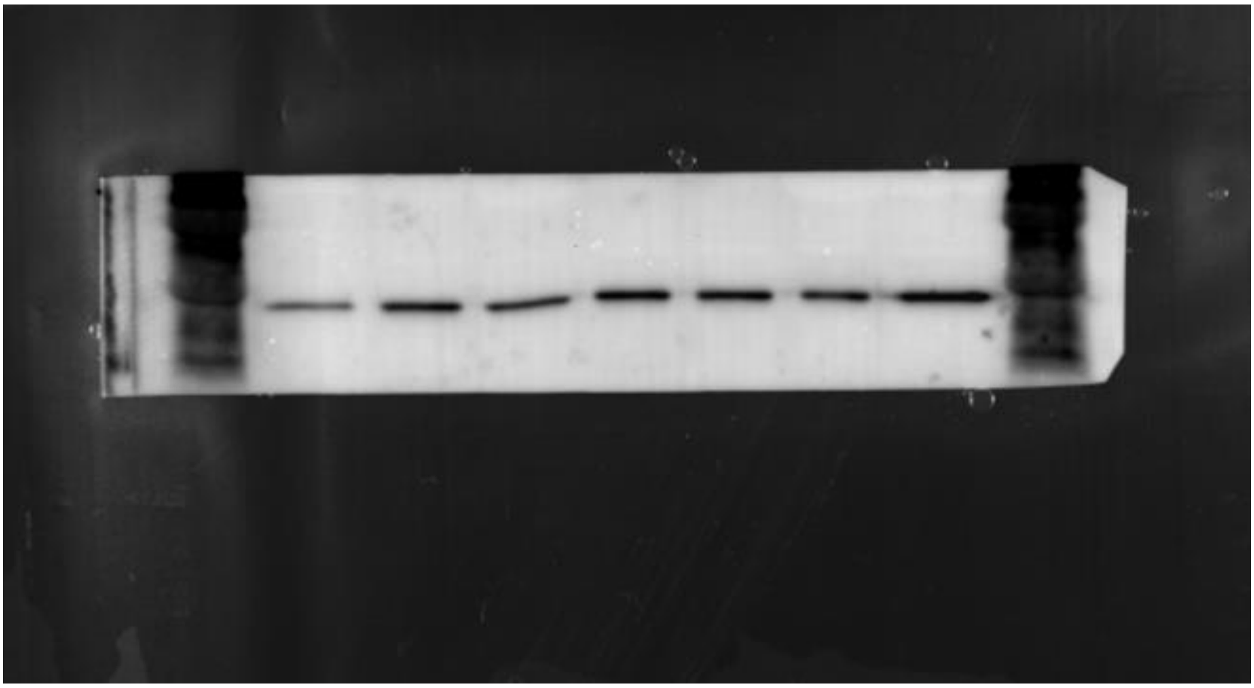

GAPDH

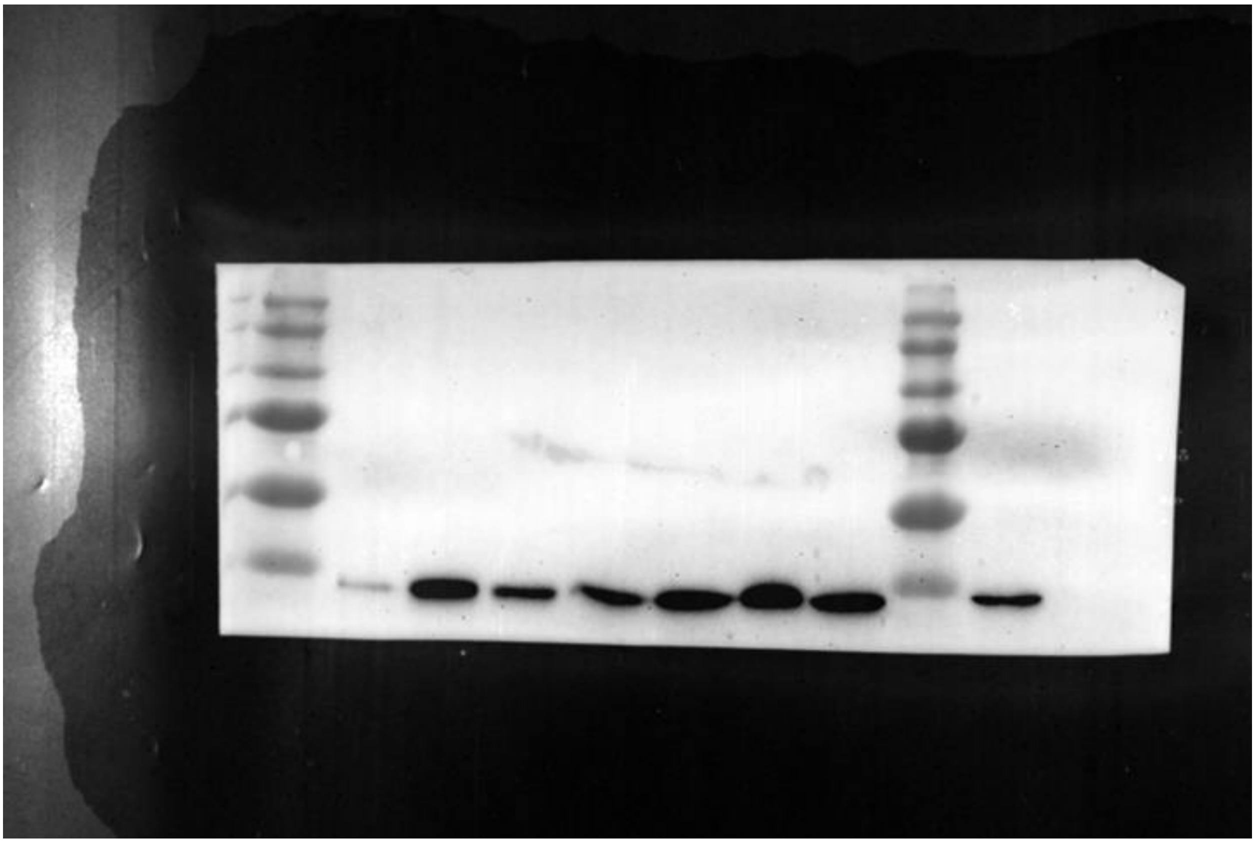

GAPDH

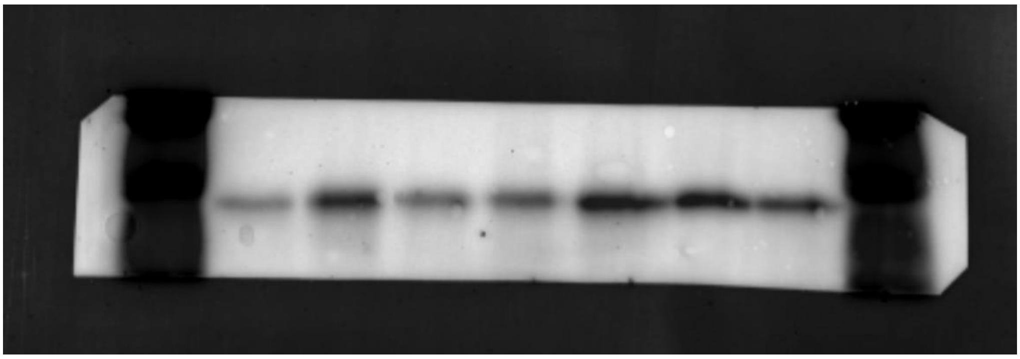

Figure S6. Uncropped membrane after blotting of polyacrylamide gel.

Table S1. List of proteins up-regulated in Kupffer cells vs monocytes and belonged to PC00262 group of proteins (metabolite interconversion enzyme) according to PANTHER (http://pantherdb.org/).

Gene ID	Mapped IDs	Gene Name, Gene symbol, ortholog	PANTHER Family/Subfamily	PANTHER Protein Class	Species
MOUSE|MGI=MGI=1929242|UniProtKB=Q9JHI5	IVD	Isovaleryl-CoA dehydrogenase, mitochondrial;Ivd;ortholog	ISOVALERYL-COA DEHYDROGENASE, MITOCHONDRIAL-RELATED (PTHR43884:SF12)	dehydrogenase(PC00092)	Mus musculus
MOUSE|MGI=MGI=1306775|UniProtKB=Q9Z2I9	SUCB1	Succinate--CoA ligase [ADP-forming] subunit beta, mitochondrial;Sucla2;ortholog	SUCCINATE--COA LIGASE [ADP-FORMING] SUBUNIT BETA, MITOCHONDRIAL (PTHR11815:SF10)	ligase(PC00142)	Mus musculus
MOUSE|MGI=MGI=1914195|UniProtKB=Q8K2B3	SDHA	Succinate dehydrogenase [ubiquinone] flavoprotein subunit, mitochondrial;Sdha;ortholog	SUCCINATE DEHYDROGENASE [UBIQUINONE] FLAVOPROTEIN SUBUNIT, MITOCHONDRIAL (PTHR11632:SF51)	dehydrogenase(PC00092)	Mus musculus
MOUSE|MGI=MGI=88035|UniProtKB=O54754	AOXA	Aldehyde oxidase 1;Aox1;ortholog	ALDEHYDE OXIDASE (PTHR11908:SF86)	oxidoreductase(PC00176)	Mus musculus
MOUSE|MGI=MGI=97394|UniProtKB=P29758	OAT	Ornithine aminotransferase, mitochondrial;Oat;ortholog	ORNITHINE AMINOTRANSFERASE, MITOCHONDRIAL (PTHR11986:SF18)	transaminase(PC00216)	Mus musculus
MOUSE|MGI=MGI=97770|UniProtKB=Q9WU79	PROD	Proline dehydrogenase 1, mitochondrial;Prodh;ortholog	PROLINE DEHYDROGENASE 1, MITOCHONDRIAL (PTHR13914:SF22)	oxidase(PC00175)	Mus musculus
MOUSE|MGI=MGI=2158650|UniProtKB=Q91VA7	Q91VA7	Isocitrate dehydrogenase [NAD] subunit, mitochondrial;Idh3b;ortholog	ISOCITRATE DEHYDROGENASE [NAD] SUBUNIT BETA, MITOCHONDRIAL (PTHR11835:SF42)	dehydrogenase(PC00092)	Mus musculus
MOUSE|MGI=MGI=96916|UniProtKB=Q8BW75	AOFB	Amine oxidase [flavin-containing] B;Maob;ortholog	AMINE OXIDASE [FLAVIN-CONTAINING] B (PTHR43563:SF5)	oxidase(PC00175)	Mus musculus
MOUSE|MGI=MGI=96078|UniProtKB=O09173	HGD	Homogentisate 1,2-dioxygenase;Hgd;ortholog	HOMOGENTISATE 1,2-DIOXYGENASE (PTHR11056:SF0)	oxygenase(PC00177)	Mus musculus
MOUSE|MGI=MGI=1917592|UniProtKB=Q8BWM0	PGES2	Prostaglandin E synthase 2;Ptges2;ortholog	PROSTAGLANDIN E SYNTHASE 2 (PTHR12782:SF5)	reductase(PC00198)	Mus musculus
MOUSE|MGI=MGI=97797|UniProtKB=P22437	COX1	Prostaglandin G/H synthase 1;Ptgs1;ortholog	PROSTAGLANDIN G/H SYNTHASE 1 (PTHR11903:SF6)	oxygenase(PC00177)	Mus musculus
MOUSE|MGI=MGI=2142985|UniProtKB=Q3TNA1	XYLB	Xylulose kinase;Xylb;ortholog	XYLULOSE KINASE (PTHR10196:SF57)	carbohydrate kinase(PC00065)	Mus musculus
MOUSE|MGI=MGI=2136976|UniProtKB=Q91WM2	HDHD5	Haloacid dehalogenase-like hydrolase domain-containing 5;Hdhd5;ortholog	HALOACID DEHALOGENASE-LIKE HYDROLASE DOMAIN-CONTAINING 5 (PTHR14269:SF17)	transferase(PC00220)	Mus musculus
MOUSE|MGI=MGI=1330818|UniProtKB=Q8BND5	SOX	Sulfhydryl oxidase 1;Qsox1;ortholog	SULFHYDRYL OXIDASE 1 (PTHR22897:SF6)	oxidase(PC00175)	Mus musculus
MOUSE|MGI=MGI=1926952|UniProtKB=P56395	CYB5	Cytochrome b5;Cyb5a;ortholog	CYTOCHROME B5 (PTHR19359:SF105)	oxidoreductase(PC00176)	Mus musculus
MOUSE|MGI=MGI=106362|UniProtKB=Q5SUC9	SCO1	Protein SCO1 homolog, mitochondrial;Sco1;ortholog	PROTEIN SCO1 HOMOLOG, MITOCHONDRIAL (PTHR12151:SF4)	oxidase(PC00175)	Mus musculus
MOUSE|MGI=MGI=108186|UniProtKB=Q9DBF1	AL7A1	Alpha-aminoadipic semialdehyde dehydrogenase;Aldh7a1;ortholog	ALPHA-AMINOADIPIC SEMIALDEHYDE DEHYDROGENASE (PTHR43521:SF1)	dehydrogenase(PC00092)	Mus musculus
MOUSE|MGI=MGI=95485|UniProtKB=P19096	FAS	Fatty acid synthase;Fasn;ortholog	FATTY ACID SYNTHASE (PTHR43775:SF7)	acyltransferase(PC00042);ligase(PC00142);hydrolase(PC00121);oxidoreductase(PC00176)	Mus musculus
MOUSE|MGI=MGI=1918951|UniProtKB=Q8K1R3	PNPT1	Polyribonucleotide nucleotidyltransferase 1, mitochondrial;Pnpt1;ortholog	POLYRIBONUCLEOTIDE NUCLEOTIDYLTRANSFERASE 1, MITOCHONDRIAL (PTHR11252:SF0)	nucleotidyltransferase(PC00174)	Mus musculus
MOUSE|MGI=MGI=2143535|UniProtKB=Q8VC97	BUP1	Beta-ureidopropionase;Upb1;ortholog	BETA-UREIDOPROPIONASE (PTHR43674:SF2)	hydrolase(PC00121)	Mus musculus
MOUSE|MGI=MGI=1913745|UniProtKB=Q9CQZ6	NDUB3	NADH dehydrogenase [ubiquinone] 1 beta subcomplex subunit 3;Ndufb3;ortholog	NADH DEHYDROGENASE [UBIQUINONE] 1 BETA SUBCOMPLEX SUBUNIT 3 (PTHR15082:SF2)	oxidoreductase(PC00176)	Mus musculus
MOUSE|MGI=MGI=1914198|UniProtKB=Q9D7B6	ACAD8	Isobutyryl-CoA dehydrogenase, mitochondrial;Acad8;ortholog	ISOBUTYRYL-COA DEHYDROGENASE, MITOCHONDRIAL (PTHR43831:SF1)	dehydrogenase(PC00092)	Mus musculus
MOUSE|MGI=MGI=99778|UniProtKB=Q64521	GPDM	Glycerol-3-phosphate dehydrogenase, mitochondrial;Gpd2;ortho	GLYCEROL-3-PHOSPHATE DEHYDROGENASE,	dehydrogenase(PC00092)	Mus musculus

		log	MITOCHONDRIAL (PTHR11985:SF15)		
MOUSE|MGI=MGI=1277964|UniProtKB=Q9DBM2	ECHP	Peroxisomal bifunctional enzyme;Ehhadh;ortholog	PEROXISOMAL BIFUNCTIONAL ENZYME (PTHR23309:SF9)	dehydrogenase(PC00092)	Mus musculus
MOUSE|MGI=MGI=2384567|UniProtKB=Q91VT4	CBR4	Carbonyl reductase family member 4;Cbr4;ortholog	CARBONYL REDUCTASE FAMILY MEMBER 4 (PTHR42760:SF81)	oxidoreductase(PC00176)	Mus musculus
MOUSE|MGI=MGI=1916167|UniProtKB=Q9D0S9	HINT2	Histidine triad nucleotide-binding protein 2, mitochondrial;Hint2;ortholog	HISTIDINE TRIAD NUCLEOTIDE-BINDING PROTEIN 2, MITOCHONDRIAL (PTHR23089:SF18)	nucleotide phosphatase(PC00173)	Mus musculus
MOUSE|MGI=MGI=87929|UniProtKB=P28474	ADHX	Alcohol dehydrogenase class-3;Adh5;ortholog	ALCOHOL DEHYDROGENASE CLASS-3 (PTHR43880:SF4)	dehydrogenase(PC00092)	Mus musculus
MOUSE|MGI=MGI=1929657|UniProtKB=Q9QXE0	HACL1	2-hydroxyacyl-CoA lyase 1;Hacl1;ortholog	2-HYDROXYACYL-COA LYASE 1 (PTHR43710:SF2)	lyase(PC00144)	Mus musculus
MOUSE|MGI=MGI=95852|UniProtKB=P51855	GSHB	Glutathione synthetase;Gss;ortholog	GLUTATHIONE SYNTHETASE (PTHR11130:SF0)	ligase(PC00142)	Mus musculus
MOUSE|MGI=MGI=1914930|UniProtKB=Q9CQA3	SDHB	Succinate dehydrogenase [ubiquinone] iron-sulfur subunit, mitochondrial;Sdhb;ortholog	SUCCINATE DEHYDROGENASE [UBIQUINONE] IRON-SULFUR SUBUNIT, MITOCHONDRIAL (PTHR11921:SF29)	dehydrogenase(PC00092)	Mus musculus
MOUSE|MGI=MGI=1890410|UniProtKB=Q9QXG4	ACSA	Acetyl-coenzyme A synthetase, cytoplasmic;Acss2;ortholog	ACETYL-COENZYME A SYNTHETASE, CYTOPLASMIC (PTHR24095:SF126)	ligase(PC00142)	Mus musculus
MOUSE|MGI=MGI=1351627|UniProtKB=Q8BKZ9	ODPX	Pyruvate dehydrogenase protein X component, mitochondrial;Pdhx;ortholog	PYRUVATE DEHYDROGENASE PROTEIN X COMPONENT, MITOCHONDRIAL (PTHR23151:SF57)	acetyltransferase(PC00038)	Mus musculus
MOUSE|MGI=MGI=1915430|UniProtKB=Q8R1F5	HYI	Putative hydroxypyruvate isomerase;Hyi;ortholog	HYDROXYPYRUVATE ISOMERASE-RELATED (PTHR43489:SF6)	isomerase(PC00135)	Mus musculus
MOUSE|MGI=MGI=2443582|UniProtKB=P61922	GABT	4-aminobutyrate aminotransferase, mitochondrial;Abat;ortholog	4-AMINOBUTYRATE AMINOTRANSFERASE, MITOCHONDRIAL (PTHR43206:SF1)	transaminase(PC00216)	Mus musculus
MOUSE|MGI=MGI=1914175|UniProtKB=Q9CXV1	DHSD	Succinate dehydrogenase [ubiquinone] cytochrome b small subunit, mitochondrial;Sdhd;ortholog	SUCCINATE DEHYDROGENASE [UBIQUINONE] CYTOCHROME B SMALL SUBUNIT, MITOCHONDRIAL (PTHR13337:SF2)	oxidoreductase(PC00176)	Mus musculus
MOUSE|MGI=MGI=95679|UniProtKB=P13707	GPDA	Glycerol-3-phosphate dehydrogenase [NAD(+)], cytoplasmic;Gpd1;ortholog	GLYCEROL-3-PHOSPHATE DEHYDROGENASE [NAD(+)], CYTOPLASMIC (PTHR11728:SF32)	dehydrogenase(PC00092)	Mus musculus
MOUSE|MGI=MGI=895149|UniProtKB=P50544	ACADV	Very long-chain specific acyl-CoA dehydrogenase, mitochondrial;Acadvl;ortholog	VERY LONG-CHAIN SPECIFIC ACYL-COA DEHYDROGENASE, MITOCHONDRIAL (PTHR43884:SF11)	dehydrogenase(PC00092)	Mus musculus
MOUSE|MGI=MGI=2677849|UniProtKB=Q71RI9	KAT3	Kynurenine--oxoglutarate transaminase 3;Kyat3;ortholog	KYNURENINE--OXOGLUTARATE TRANSAMINASE 3 (PTHR43807:SF6)	transaminase(PC00216)	Mus musculus
MOUSE|MGI=MGI=95753|UniProtKB=P26443	DHE3	Glutamate dehydrogenase 1, mitochondrial;Glud1;ortholog	GLUTAMATE DEHYDROGENASE 1, MITOCHONDRIAL (PTHR11606:SF13)	dehydrogenase(PC00092)	Mus musculus
MOUSE|MGI=MGI=1913666|UniProtKB=Q9Z1P6	NDUA7	NADH dehydrogenase [ubiquinone] 1 alpha subcomplex subunit 7;Ndufa7;ortholog	NADH DEHYDROGENASE [UBIQUINONE] 1 ALPHA SUBCOMPLEX SUBUNIT 7 (PTHR12485:SF1)	oxidoreductase(PC00176)	Mus musculus
MOUSE|MGI=MGI=2444085|UniProtKB=Q8QZY2	GLCTK	Glycerate kinase;Glyctk;ortholog	GLYCERATE KINASE (PTHR12227:SF0)	carbohydrate kinase(PC00065)	Mus musculus
MOUSE|MGI=MGI=1914380|UniProtKB=Q9CQZ5	NDUA6	NADH dehydrogenase [ubiquinone] 1 alpha subcomplex subunit 6;Ndufa6;ortholog	NADH DEHYDROGENASE [UBIQUINONE] 1 ALPHA SUBCOMPLEX SUBUNIT 6 (PTHR12964:SF0)	dehydrogenase(PC00092)	Mus musculus
MOUSE|MGI=MGI=87968|UniProtKB=P50247	SAHH	Adenosylhomocysteinase;Ahcy;ortholog	ADENOSYLHOMOCYSTEINASE (PTHR23420:SF0)	hydrolase(PC00121)	Mus musculus
MOUSE|MGI=MGI=1860517|UniProtKB=O88451	RDH7	Retinol dehydrogenase 7;Rdh7;ortholog	RETINOL DEHYDROGENASE 16 (PTHR43313:SF11)	dehydrogenase(PC00092)	Mus musculus
MOUSE|MGI=MGI=88017|UniProtKB=Q91X83	METK1	S-adenosylmethionine synthase isoform type-1;Mat1a;ortholog	S-ADENOSYLMETHIONINE SYNTHASE ISOFORM TYPE-1 (PTHR11964:SF11)	nucleotidyltransferase(PC00174)	Mus musculus
MOUSE|MGI=MGI=1919289|UniProtKB=Q99MR8	MCCA	Methylcrotonoyl-CoA carboxylase subunit alpha,	METHYLCROTONOYL-COA CARBOXYLASE SUBUNIT ALPHA,	ligase(PC00142)	Mus musculus

		mitochondrial;Mccc1;ortholog	MITOCHONDRIAL-RELATED (PTHR18866:SF33)		
MOUSE|MGI=MGI=1343103|UniProtKB=Q9CQ75	NDUA2	NADH dehydrogenase [ubiquinone] 1 alpha subcomplex subunit 2;Ndufa2;ortholog	NADH DEHYDROGENASE [UBIQUINONE] 1 ALPHA SUBCOMPLEX SUBUNIT 2 (PTHR12878:SF4)	oxidoreductase(PC00176)	Mus musculus
MOUSE|MGI=MGI=107686|UniProtKB=Q62425	NDUA4	Cytochrome c oxidase subunit NDUFA4;Ndufa4;ortholog	CYTOCHROME C OXIDASE SUBUNIT NDUFA4 (PTHR14256:SF4)	oxidoreductase(PC00176)	Mus musculus
MOUSE|MGI=MGI=1277169|UniProtKB=Q9D9V3	ECHD1	Ethylmalonyl-CoA decarboxylase;Echdc1;ortholog	ETHYLMALONYL-COA DECARBOXYLASE (PTHR11941:SF27)	lyase(PC00144)	Mus musculus
MOUSE|MGI=MGI=96158|UniProtKB=P38060	HMGCL	Hydroxymethylglutaryl-CoA lyase, mitochondrial;Hmgcl;ortholog	HYDROXYMETHYLGLUTARYL-COA LYASE, MITOCHONDRIAL (PTHR42738:SF1)	lyase(PC00144)	Mus musculus
MOUSE|MGI=MGI=1306824|UniProtKB=Q9Z2I8	SUCB2	Succinate--CoA ligase [GDP-forming] subunit beta, mitochondrial;Suclg2;ortholog	SUCCINATE--COA LIGASE [GDP-FORMING] SUBUNIT BETA, MITOCHONDRIAL (PTHR11815:SF17)	ligase(PC00142)	Mus musculus
MOUSE|MGI=MGI=1913826|UniProtKB=P99028	QCR6	Cytochrome b-c1 complex subunit 6, mitochondrial;Uqcrh;ortholog	CYTOCHROME B-C1 COMPLEX SUBUNIT 6, MITOCHONDRIAL (PTHR15336:SF0)	reductase(PC00198)	Mus musculus
MOUSE|MGI=MGI=88271|UniProtKB=P24270	CATA	Catalase;Cat;ortholog	CATALASE (PTHR11465:SF9)	peroxidase(PC00180)	Mus musculus
MOUSE|MGI=MGI=2442264|UniProtKB=P58044	IDI1	Isopentenyl-diphosphate Delta-isomerase 1;Idi1;ortholog	ISOPENTENYL-DIPHOSPHATE DELTA-ISOMERASE 1 (PTHR10885:SF5)	isomerase(PC00135)	Mus musculus
MOUSE|MGI=MGI=1096386|UniProtKB=P51658	DHB2	Estradiol 17-beta-dehydrogenase 2;Hsd17b2;ortholog	ESTRADIOL 17-BETA-DEHYDROGENASE 2 (PTHR43313:SF3)	dehydrogenase(PC00092)	Mus musculus
MOUSE|MGI=MGI=104990|UniProtKB=P97494	GSH1	Glutamate--cysteine ligase catalytic subunit;Gclc;ortholog	GLUTAMATE--CYSTEINE LIGASE CATALYTIC SUBUNIT (PTHR11164:SF0)	ligase(PC00142)	Mus musculus
MOUSE|MGI=MGI=2429497|UniProtKB=Q8VHG0	FMO4	Dimethylaniline monooxygenase [N-oxide-forming] 4;Fmo4;ortholog	DIMETHYLANILINE MONOOXYGENASE [N-OXIDE-FORMING] 4 (PTHR23023:SF74)	oxygenase(PC00177)	Mus musculus
MOUSE|MGI=MGI=99538|UniProtKB=Q3UNX5	ACSM3	Acyl-coenzyme A synthetase ACSM3, mitochondrial;Acsm3;ortholog	ACYL-COENZYME A SYNTHETASE ACSM3, MITOCHONDRIAL (PTHR43605:SF7)	ligase(PC00142)	Mus musculus
MOUSE|MGI=MGI=95420|UniProtKB=P23953	ES1,EST1C	Carboxylesterase 1C;Ces1c;ortholog	CARBOXYLESTERASE 1C-RELATED (PTHR11559:SF192)	esterase(PC00097)	Mus musculus
MOUSE|MGI=MGI=1860835|UniProtKB=Q9WTP7	KAD3	GTP:AMP phosphotransferase AK3, mitochondrial;Ak3;ortholog	GTP:AMP PHOSPHOTRANSFERASE AK3, MITOCHONDRIAL (PTHR23359:SF68)	nucleotide kinase(PC00172)	Mus musculus
MOUSE|MGI=MGI=1861622|UniProtKB=Q9JLJ2	AL9A1	4-trimethylaminobutyraldehyde dehydrogenase;Aldh9a1;ortholog	4-TRIMETHYLAMINOBUTYRALDEHYDE DEHYDROGENASE (PTHR11699:SF228)	dehydrogenase(PC00092)	Mus musculus
MOUSE|MGI=MGI=88285|UniProtKB=Q91WT9	CBS	Cystathionine beta-synthase;Cbs;ortholog	CYSTATHIONINE BETA-SYNTHASE-RELATED (PTHR10314:SF177)	lyase(PC00144)	Mus musculus
MOUSE|MGI=MGI=1333871|UniProtKB=O08756	HCD2	3-hydroxyacyl-CoA dehydrogenase type-2;Hsd17b10;ortholog	3-HYDROXYACYL-COA DEHYDROGENASE TYPE-2 (PTHR43658:SF12)	oxidoreductase(PC00176)	Mus musculus
MOUSE|MGI=MGI=1933427|UniProtKB=P70694	DHB5	Estradiol 17 beta-dehydrogenase 5;Akr1c6;ortholog	ALDO-KETO REDUCTASE FAMILY 1 MEMBER C3 (PTHR11732:SF165)	reductase(PC00198)	Mus musculus
MOUSE|MGI=MGI=1916142|UniProtKB=Q9CRB3	HIUH	5-hydroxyisourate hydrolase;Urah;ortholog	5-HYDROXYISOURATE HYDROLASE (PTHR10395:SF11)	hydrolase(PC00121)	Mus musculus
MOUSE|MGI=MGI=2145458|UniProtKB=Q91WT7	Q91WT7	3-alpha-hydroxysteroid dehydrogenase type 1;Akr1c14;ortholog	3-ALPHA-HYDROXYSTEROID DEHYDROGENASE TYPE 1 (PTHR11732:SF205)	reductase(PC00198)	Mus musculus
MOUSE|MGI=MGI=2385112|UniProtKB=Q91WD5	NDUS2	NADH dehydrogenase [ubiquinone] iron-sulfur protein 2, mitochondrial;Ndufs2;ortholog	NADH DEHYDROGENASE [UBIQUINONE] IRON-SULFUR PROTEIN 2, MITOCHONDRIAL (PTHR11993:SF10)	oxidoreductase(PC00176)	Mus musculus
MOUSE|MGI=MGI=3644960|UniProtKB=Q8VCU1	EST3B	Carboxylesterase 3B;Ces3b;ortholog	CARBOXYLESTERASE 3 (PTHR11559:SF259)	esterase(PC00097)	Mus musculus
MOUSE|MGI=MGI=87868|UniProtKB=Q07417	ACADS	Short-chain specific acyl-CoA dehydrogenase, mitochondrial;Acads;ortholog	ISOVALERYL-COA DEHYDROGENASE, MITOCHONDRIAL-RELATED (PTHR43884:SF12)	dehydrogenase(PC00092)	Mus musculus
MOUSE|MGI=MGI=97831|UniProtKB=Q9D819	IPYR	Inorganic pyrophosphatase;Ppa1;o	INORGANIC PYROPHOSPHATASE	pyrophosphatase(PC00196)	Mus musc

		rtholog	(PTHR10286:SF47)		ulus
MOUSE|MGI=MGI=1913468|UniProtKB=Q9CQJ8	NDUB9	NADH dehydrogenase [ubiquinone] 1 beta subcomplex subunit 9;Ndufb9;ortholog	NADH DEHYDROGENASE [UBIQUINONE] 1 BETA SUBCOMPLEX SUBUNIT 9 (PTHR12868:SF0)	oxidoreductase(PC00176)	Mus musculus
MOUSE|MGI=MGI=2136381|UniProtKB=Q99JY0	ECHB	Trifunctional enzyme subunit beta, mitochondrial;Hadhb;ortholog	TRIFUNCTIONAL ENZYME SUBUNIT BETA, MITOCHONDRIAL (PTHR18919:SF153)	acyltransferase(PC00042)	Mus musculus
MOUSE|MGI=MGI=1920974|UniProtKB=Q9D1I5	MCEE	Methylmalonyl-CoA epimerase, mitochondrial;Mcee;ortholog	METHYLMALONYL-COA EPIMERASE, MITOCHONDRIAL (PTHR43048:SF3)	epimerase/racemase(PC00096)	Mus musculus
MOUSE|MGI=MGI=107796|UniProtKB=Q8CG76	ARK72	Aflatoxin B1 aldehyde reductase member 2;Akr7a2;ortholog	AFLATOXIN B1 ALDEHYDE REDUCTASE MEMBER 2 (PTHR43625:SF12)	reductase(PC00198)	Mus musculus
MOUSE|MGI=MGI=2385079|UniProtKB=Q8K3J1	NDUS8	NADH dehydrogenase [ubiquinone] iron-sulfur protein 8, mitochondrial;Ndufs8;ortholog	NADH DEHYDROGENASE [UBIQUINONE] IRON-SULFUR PROTEIN 8, MITOCHONDRIAL (PTHR10849:SF20)	oxidoreductase(PC00176)	Mus musculus
MOUSE|MGI=MGI=1197006|UniProtKB=Q9D826	SOX	Peroxisomal sarcosine oxidase;Pipox;ortholog	PEROXISOMAL SARCOSINE OXIDASE (PTHR10961:SF7)	oxidase(PC00175)	Mus musculus
MOUSE|MGI=MGI=95492|UniProtKB=Q9QXD6	F16P1	Fructose-1,6-bisphosphatase 1;Fbp1;ortholog	FRUCTOSE-1,6-BISPHOSPHATASE 1 (PTHR11556:SF11)	carbohydrate phosphatase(PC00066)	Mus musculus
MOUSE|MGI=MGI=1915921|UniProtKB=Q922E4	PCY2	Ethanolamine-phosphate cytidylyltransferase;Pcyt2;ortholog	ETHANOLAMINE-PHOSPHATE CYTIDYLYLTRANSFERASE (PTHR45780:SF2)	nucleotidyltransferase(PC00174)	Mus musculus
MOUSE|MGI=MGI=2384968|UniProtKB=Q91YP0	L2HDH	L-2-hydroxyglutarate dehydrogenase, mitochondrial;L2hgdh;ortholog	L-2-HYDROXYGLUTARATE DEHYDROGENASE, MITOCHONDRIAL (PTHR43104:SF2)	dehydrogenase(PC00092)	Mus musculus
MOUSE|MGI=MGI=2182591|UniProtKB=Q3URE1	ACSF3	Acyl-CoA synthetase family member 3, mitochondrial;Acsf3;ortholog	ACYL-COA SYNTHETASE FAMILY MEMBER 3, MITOCHONDRIAL (PTHR24096:SF267)	ligase(PC00142)	Mus musculus
MOUSE|MGI=MGI=97043|UniProtKB=P06801	MAOX	NADP-dependent malic enzyme;Me1;ortholog	NADP-DEPENDENT MALIC ENZYME (PTHR23406:SF17)	oxidoreductase(PC00176)	Mus musculus
MOUSE|MGI=MGI=102504|UniProtKB=P00397	COX1	Cytochrome c oxidase subunit 1;Mtco1;ortholog	CYTOCHROME C OXIDASE SUBUNIT 1 (PTHR10422:SF18)	oxidase(PC00175)	Mus musculus
MOUSE|MGI=MGI=2444835|UniProtKB=Q8BGC4	PTGR3	Prostaglandin reductase-3;Zadh2;ortholog	PROSTAGLANDIN REDUCTASE 3 (PTHR43677:SF3)	oxidoreductase(PC00176)	Mus musculus
MOUSE|MGI=MGI=1916406|UniProtKB=Q8BIG7	CMTD1	Catechol O-methyltransferase domain-containing protein 1;Comtd1;ortholog	CATECHOL O-METHYLTRANSFERASE DOMAIN-CONTAINING PROTEIN 1 (PTHR10509:SF14)	methyltransferase(PC00155)	Mus musculus
MOUSE|MGI=MGI=97798|UniProtKB=Q05769	COX2	Prostaglandin G/H synthase 2;Ptgs2;ortholog	PROSTAGLANDIN G/H SYNTHASE 2 (PTHR11903:SF8)	oxygenase(PC00177)	Mus musculus
MOUSE|MGI=MGI=1914154|UniProtKB=Q99MN9	PCCB	Propionyl-CoA carboxylase beta chain, mitochondrial;Pccb;ortholog	PROPIONYL-COA CARBOXYLASE BETA CHAIN, MITOCHONDRIAL (PTHR43842:SF2)	ligase(PC00142)	Mus musculus
MOUSE|MGI=MGI=88589|UniProtKB=P00186	CP1A2	Cytochrome P450 1A2;Cyp1a2;ortholog	CYTOCHROME P450 1A2 (PTHR24299:SF12)	oxygenase(PC00177)	Mus musculus
MOUSE|MGI=MGI=104888|UniProtKB=Q920E5	FPPS	Farnesyl pyrophosphate synthase;Fdps;ortholog	FARNESYL PYROPHOSPHATE SYNTHASE (PTHR11525:SF0)	acyltransferase(PC00042)	Mus musculus
MOUSE|MGI=MGI=1914523|UniProtKB=Q99LC3	NDUAA	NADH dehydrogenase [ubiquinone] 1 alpha subcomplex subunit 10, mitochondrial;Ndufa10;ortholog	NADH DEHYDROGENASE [UBIQUINONE] 1 ALPHA SUBCOMPLEX SUBUNIT 10, MITOCHONDRIAL (PTHR10513:SF15)	nucleotide kinase(PC00172)	Mus musculus
MOUSE|MGI=MGI=98352|UniProtKB=P09671	SODM	Superoxide dismutase [Mn], mitochondrial;Sod2;ortholog	SUPEROXIDE DISMUTASE [MN], MITOCHONDRIAL (PTHR11404:SF6)	oxidoreductase(PC00176)	Mus musculus
MOUSE|MGI=MGI=2685720|UniProtKB=Q14DH7	ACSS3	Acyl-CoA synthetase short-chain family member 3, mitochondrial;Acss3;ortholog	ACYL-COA SYNTHETASE SHORT-CHAIN FAMILY MEMBER 3, MITOCHONDRIAL (PTHR43347:SF3)	ligase(PC00142)	Mus musculus
MOUSE|MGI=MGI=2442664|UniProtKB=Q8CC86	PNCB	Nicotinate phosphoribosyltransferase;Naprt;ortholog	NICOTINATE PHOSPHORIBOSYLTRANSFERASE (PTHR11098:SF1)	glycosyltransferase(PC00111)	Mus musculus
MOUSE|MGI=MGI=98852|UniProtKB=P52196	THTR	Thiosulfate sulfurtransferase;Tst;ortholog	THIOSULFATE SULFURTRANSFERASE (PTHR11364:SF6)	transferase(PC00220)	Mus musculus
MOUSE|MGI=MGI=99500|UniProtKB=P34914	HYES	Bifunctional epoxide hydrolase	BIFUNCTIONAL EPOXIDE HYDROLASE	hydrolase(PC00121)	Mus musc

		2;Ephx2;ortholog	2 (PTHR43329:SF4)		ulus
MOUSE|MGI=MGI=1349389|UniProtKB=O88986	KBL	2-amino-3-ketobutyrate coenzyme A ligase, mitochondrial;Gcat;ortholog	2-AMINO-3-KETOBUTYRATE COENZYME A LIGASE, MITOCHONDRIAL (PTHR13693:SF60)	transaminase(PC00216)	Mus musculus
MOUSE|MGI=MGI=1915452|UniProtKB=Q9CPP6	NDUA5	NADH dehydrogenase [ubiquinone] 1 alpha subcomplex subunit 5;Ndufa5;ortholog	NADH DEHYDROGENASE [UBIQUINONE] 1 ALPHA SUBCOMPLEX SUBUNIT 5 (PTHR12653:SF0)	oxidoreductase(PC00176)	Mus musculus
MOUSE|MGI=MGI=1915625|UniProtKB=Q9DCJ5	NDUA8	NADH dehydrogenase [ubiquinone] 1 alpha subcomplex subunit 8;Ndufa8;ortholog	NADH DEHYDROGENASE [UBIQUINONE] 1 ALPHA SUBCOMPLEX SUBUNIT 8 (PTHR13344:SF0)	dehydrogenase(PC00092)	Mus musculus
MOUSE|MGI=MGI=102581|UniProtKB=Q9QYF1	RDH11	Retinol dehydrogenase 11;Rdh11;ortholog	RETINOL DEHYDROGENASE 11 (PTHR24320:SF108)	dehydrogenase(PC00092)	Mus musculus
MOUSE|MGI=MGI=1340024|UniProtKB=Q8R0Y6	AL1L1	Cytosolic 10-formyltetrahydrofolate dehydrogenase;Aldh1l1;ortholog	CYTOSOLIC 10-FORMYLTETRAHYDROFOLATE DEHYDROGENASE (PTHR11699:SF120)	dehydrogenase(PC00092)	Mus musculus
MOUSE|MGI=MGI=1922656|UniProtKB=Q9DC70	NDUS7	NADH dehydrogenase [ubiquinone] iron-sulfur protein 7, mitochondrial;Ndufs7;ortholog	NADH DEHYDROGENASE [UBIQUINONE] IRON-SULFUR PROTEIN 7, MITOCHONDRIAL (PTHR11995:SF14)	dehydrogenase(PC00092)	Mus musculus
MOUSE|MGI=MGI=2443170|UniProtKB=Q8BK48	EST2E	Pyrethroid hydrolase Ces2e;Ces2e;ortholog	ACYLCARNITINE HYDROLASE-RELATED (PTHR11559:SF400)	esterase(PC00097)	Mus musculus
MOUSE|MGI=MGI=106100|UniProtKB=Q921G7	ETFD	Electron transfer flavoprotein-ubiquinone oxidoreductase, mitochondrial;Etfdh;ortholog	ELECTRON TRANSFER FLAVOPROTEIN-UBIQUINONE OXIDOREDUCTASE, MITOCHONDRIAL (PTHR10617:SF107)	oxidoreductase(PC00176)	Mus musculus
MOUSE|MGI=MGI=1350916|UniProtKB=Q8VDK1	NIT1	Deaminated glutathione amidase;Nit1;ortholog	DEAMINATED GLUTATHIONE AMIDASE (PTHR23088:SF27)	hydrolase(PC00121)	Mus musculus
MOUSE|MGI=MGI=2652816|UniProtKB=Q3U0B3	DHR11	Dehydrogenase/reductase SDR family member 11;Dhrs11;ortholog	DEHYDROGENASE/REDUCTASE SDR FAMILY MEMBER 11 (PTHR43115:SF4)	oxidoreductase(PC00176)	Mus musculus
MOUSE|MGI=MGI=97050|UniProtKB=P08249	MDHM	Malate dehydrogenase, mitochondrial;Mdh2;ortholog	MALATE DEHYDROGENASE, MITOCHONDRIAL (PTHR11540:SF16)	dehydrogenase(PC00092)	Mus musculus
MOUSE|MGI=MGI=1347050|UniProtKB=O35945	AL1A7	Aldehyde dehydrogenase, cytosolic 1;Aldh1a7;ortholog	ALDEHYDE DEHYDROGENASE, CYTOSOLIC 1 (PTHR11699:SF221)	dehydrogenase(PC00092)	Mus musculus
MOUSE|MGI=MGI=2152200|UniProtKB=Q91VA0	ACSM1	Acyl-coenzyme A synthetase ACSM1, mitochondrial;Acsm1;ortholog	ACYL-COENZYME A SYNTHETASE ACSM1, MITOCHONDRIAL (PTHR43605:SF5)	ligase(PC00142)	Mus musculus
MOUSE|MGI=MGI=1916876|UniProtKB=Q5M8N4	D39U1	Epimerase family protein SDR39U1;Sdr39u1;ortholog	EPIMERASE FAMILY PROTEIN SDR39U1 (PTHR11092:SF0)	epimerase/racemase(PC00096)	Mus musculus
MOUSE|MGI=MGI=1918914|UniProtKB=Q9DD20	MET7B	Methyltransferase-like protein 7B;Mettl7b;ortholog	METHYLTRANSFERASE-LIKE PROTEIN 7B (PTHR42912:SF14)	methyltransferase(PC00155)	Mus musculus
MOUSE|MGI=MGI=88070|UniProtKB=Q61176	ARGI1	Arginase-1;Arg1;ortholog	ARGINASE-1 (PTHR43782:SF2)	hydrolase(PC00121)	Mus musculus
MOUSE|MGI=MGI=1098643|UniProtKB=Q9CXI0	COQ5	2-methoxy-6-polyprenyl-1,4-benzoquinol methylase, mitochondrial;Coq5;ortholog	2-METHOXY-6-POLYPRENYL-1,4-BENZOQUINOL METHYLASE, MITOCHONDRIAL (PTHR43591:SF24)	methyltransferase(PC00155)	Mus musculus
MOUSE|MGI=MGI=102496|UniProtKB=P03921	NU5M	NADH-ubiquinone oxidoreductase chain 5;Mtnd5;ortholog	NADH-UBIQUINONE OXIDOREDUCTASE CHAIN 5 (PTHR42829:SF2)	oxidoreductase(PC00176)	Mus musculus
MOUSE|MGI=MGI=1915337|UniProtKB=Q8BHC4	DCAKD	Dephospho-CoA kinase domain-containing protein;Dcakd;ortholog	BIFUNCTIONAL COENZYME A SYNTHASE-RELATED (PTHR10695:SF46)	kinase(PC00137)	Mus musculus
MOUSE|MGI=MGI=88034|UniProtKB=P20108	PRDX3	Thioredoxin-dependent peroxide reductase, mitochondrial;Prdx3;ortholog	THIOREDOXIN-DEPENDENT PEROXIDE REDUCTASE, MITOCHONDRIAL (PTHR42801:SF4)	peroxidase(PC00180)	Mus musculus
MOUSE|MGI=MGI=95802|UniProtKB=Q8QZR5	ALAT1	Alanine aminotransferase 1;Gpt;ortholog	ALANINE AMINOTRANSFERASE 1 (PTHR11751:SF308)	transaminase(PC00216)	Mus musculus
MOUSE|MGI=MGI=99523|UniProtKB=P35700	PRDX1	Peroxiredoxin-1;Prdx1;ortholog	PEROXIREDOXIN-1 (PTHR10681:SF111)	peroxidase(PC00180)	Mus musculus
MOUSE|MGI=MGI=88609|UniProtKB=Q64459	CP3AB	Cytochrome P450 3A11;Cyp3a11;ortholog	CYTOCHROME P450 3A11-RELATED (PTHR24302:SF33)	oxygenase(PC00177)	Mus musculus

MOUSE|MGI=MGI=2653900|UniProtKB=Q8BH00	AL8A1	2-aminomuconic semialdehyde dehydrogenase;Aldh8a1;ortholog	2-AMINOMUCONIC SEMIALDEHYDE DEHYDROGENASE (PTHR43720:SF2)	dehydrogenase(PC00092)	Mus musculus
MOUSE|MGI=MGI=1914514|UniProtKB=Q9D6J5	NDUB8	NADH dehydrogenase [ubiquinone] 1 beta subcomplex subunit 8, mitochondrial;Ndufb8;ortholog	NADH DEHYDROGENASE [UBIQUINONE] 1 BETA SUBCOMPLEX SUBUNIT 8, MITOCHONDRIAL (PTHR12840:SF1)	oxidoreductase(PC00176)	Mus musculus
MOUSE|MGI=MGI=2142491|UniProtKB=Q8QZR3	EST2A	Pyrethroid hydrolase Ces2a;Ces2a;ortholog	PYRETHROID HYDROLASE CES2A (PTHR11559:SF174)	esterase(PC00097)	Mus musculus
MOUSE|MGI=MGI=1298381|UniProtKB=O35215	DOPD	D-dopachrome decarboxylase;Ddt;ortholog	D-DOPACHROME DECARBOXYLASE (PTHR11954:SF22)	decarboxylase(PC00089)	Mus musculus
MOUSE|MGI=MGI=1928679|UniProtKB=Q9EQF5	DPYS	Dihydropyrimidinase;Dpys;ortholog	DIHYDROPYRIMIDINASE (PTHR11647:SF50)	hydrolase(PC00121)	Mus musculus
MOUSE|MGI=MGI=88084|UniProtKB=Q91YI0	ARLY	Argininosuccinate lyase;Asl;ortholog	ARGININOSUCCINATE LYASE (PTHR43814:SF1)	lyase(PC00144)	Mus musculus
MOUSE|MGI=MGI=106098|UniProtKB=Q9DCW4	ETFB	Electron transfer flavoprotein subunit beta;Etfb;ortholog	ELECTRON TRANSFER FLAVOPROTEIN SUBUNIT BETA (PTHR21294:SF8)	hydroxylase(PC00122)	Mus musculus
MOUSE|MGI=MGI=102773|UniProtKB=Q63880	EST3A	Carboxylesterase 3A;Ces3a;ortholog	CARBOXYLESTERASE 3 (PTHR11559:SF259)	esterase(PC00097)	Mus musculus
MOUSE|MGI=MGI=1923488|UniProtKB=Q91Z53	GRHPR	Glyoxylate reductase/hydroxypyruvate reductase;Grhpr;ortholog	GLYOXYLATE REDUCTASE/HYDROXYPYRUVATE REDUCTASE (PTHR10996:SF137)	dehydrogenase(PC00092)	Mus musculus
MOUSE|MGI=MGI=1914084|UniProtKB=Q9CQR4	ACO13	Acyl-coenzyme A thioesterase 13;Acot13;ortholog	ACYL-COENZYME A THIOESTERASE 13 (PTHR21660:SF1)	esterase(PC00097)	Mus musculus
MOUSE|MGI=MGI=1913321|UniProtKB=Q9DCM0	ETHE1	Persulfide dioxygenase ETHE1, mitochondrial;Ethe1;ortholog	PERSULFIDE DIOXYGENASE ETHE1, MITOCHONDRIAL (PTHR43084:SF1)	oxygenase(PC00177)	Mus musculus
MOUSE|MGI=MGI=99600|UniProtKB=P47738	ALDH2	Aldehyde dehydrogenase, mitochondrial;Aldh2;ortholog	ALDEHYDE DEHYDROGENASE, MITOCHONDRIAL (PTHR11699:SF233)	dehydrogenase(PC00092)	Mus musculus
MOUSE|MGI=MGI=1913296|UniProtKB=Q9CQH3	NDUB5	NADH dehydrogenase [ubiquinone] 1 beta subcomplex subunit 5, mitochondrial;Ndufb5;ortholog	NADH DEHYDROGENASE [UBIQUINONE] 1 BETA SUBCOMPLEX SUBUNIT 5, MITOCHONDRIAL (PTHR13178:SF0)	oxidoreductase(PC00176)	Mus musculus
MOUSE|MGI=MGI=107807|UniProtKB=Q9CQ69	QCR8	Cytochrome b-c1 complex subunit 8;Uqcrq;ortholog	CYTOCHROME B-C1 COMPLEX SUBUNIT 8 (PTHR12119:SF2)	oxidoreductase(PC00176)	Mus musculus
MOUSE|MGI=MGI=88378|UniProtKB=Q8VCC2	EST1	Liver carboxylesterase 1;Ces1;ortholog	LIVER CARBOXYLESTERASE 1-RELATED (PTHR11559:SF179)	esterase(PC00097)	Mus musculus
MOUSE|MGI=MGI=1918993|UniProtKB=Q9DBL7	COASY	Bifunctional coenzyme A synthase;Coasy;ortholog	BIFUNCTIONAL COENZYME A SYNTHASE-RELATED (PTHR10695:SF46)	kinase(PC00137)	Mus musculus
MOUSE|MGI=MGI=2135593|UniProtKB=Q8BMS1	ECHA	Trifunctional enzyme subunit alpha, mitochondrial;Hadha;ortholog	TRIFUNCTIONAL ENZYME SUBUNIT ALPHA, MITOCHONDRIAL (PTHR43612:SF3)	dehydrogenase(PC00092);hydratase(PC00120)	Mus musculus
MOUSE|MGI=MGI=95739|UniProtKB=P15105	GLNA	Glutamine synthetase;Glul;ortholog	GLUTAMINE SYNTHETASE (PTHR20852:SF45)	ligase(PC00142)	Mus musculus
MOUSE|MGI=MGI=97499|UniProtKB=Q91ZA3	PCCA	Propionyl-CoA carboxylase alpha chain, mitochondrial;Pcca;ortholog	METHYLCROTONOYL-COA CARBOXYLASE SUBUNIT ALPHA, MITOCHONDRIAL-RELATED (PTHR18866:SF33)	ligase(PC00142)	Mus musculus
MOUSE|MGI=MGI=95513|UniProtKB=P22315	HEMH	Ferrochelatase, mitochondrial;Fech;ortholog	FERROCHELATASE, MITOCHONDRIAL (PTHR11108:SF1)	lyase(PC00144)	Mus musculus
MOUSE|MGI=MGI=101813|UniProtKB=Q8BMS4	COQ3	Ubiquinone biosynthesis O-methyltransferase, mitochondrial;Coq3;ortholog	UBIQUINONE BIOSYNTHESIS O-METHYLTRANSFERASE, MITOCHONDRIAL (PTHR43464:SF19)	methyltransferase(PC00155)	Mus musculus
MOUSE|MGI=MGI=2443241|UniProtKB=Q91VD9	NDUS1	NADH-ubiquinone oxidoreductase 75 kDa subunit, mitochondrial;Ndufs1;ortholog	NADH-UBIQUINONE OXIDOREDUCTASE 75 KDA SUBUNIT, MITOCHONDRIAL (PTHR11615:SF6)	dehydrogenase(PC00092)	Mus musculus
MOUSE|MGI=MGI=106916|UniProtKB=Q9D6Y7	MSRA	Mitochondrial peptide methionine sulfoxide reductase;Msra;ortholog	MITOCHONDRIAL PEPTIDE METHIONINE SULFOXIDE REDUCTASE (PTHR42799:SF2)	reductase(PC00198)	Mus musculus
MOUSE|MGI=MGI=2444086|UniProtKB=Q8BGA8	ACSM5	Acyl-coenzyme A synthetase ACSM5, mitochondrial;Acsm5;ortholog	ACYL-COENZYME A SYNTHETASE ACSM5, MITOCHONDRIAL (PTHR43605:SF6)	ligase(PC00142)	Mus musculus

MOUSE|MGI=MGI=894320|UniProtKB=O08709	PRDX6	Peroxiredoxin-6;Prdx6;ortholog	MCG48959-RELATED (PTHR43503:SF4)	peroxidase(PC00180)	Mus musculus
MOUSE|MGI=MGI=104724|UniProtKB=Q61578	ADRO	NADPH:adrenodoxin oxidoreductase, mitochondrial;Fdxr;ortholog	NADPH:ADRENODOXIN OXIDOREDUCTASE, MITOCHONDRIAL (PTHR11938:SF91)	oxidoreductase(PC00176)	Mus musculus
MOUSE|MGI=MGI=1261838|UniProtKB=Q9JHW2	NIT2	Omega-amidase NIT2;Nit2;ortholog	OMEGA-AMIDASE NIT2 (PTHR23088:SF30)	hydrolase(PC00121)	Mus musculus
MOUSE|MGI=MGI=106428|UniProtKB=Q7TNG8	LDHD	Probable D-lactate dehydrogenase, mitochondrial;Ldhd;ortholog	D-LACTATE DEHYDROGENASE, MITOCHONDRIAL-RELATED (PTHR11748:SF111)	dehydrogenase(PC00092)	Mus musculus
MOUSE|MGI=MGI=1329033|UniProtKB=O35423	SPYA	Serine--pyruvate aminotransferase, mitochondrial;Agxt;ortholog	SERINE--PYRUVATE AMINOTRANSFERASE (PTHR21152:SF24)	transaminase(PC00216)	Mus musculus
MOUSE|MGI=MGI=1100496|UniProtKB=P97501	FMO3	Dimethylaniline monooxygenase [N-oxide-forming] 3;Fmo3;ortholog	DIMETHYLANILINE MONOOXYGENASE [N-OXIDE-FORMING] 3 (PTHR23023:SF44)	oxygenase(PC00177)	Mus musculus
MOUSE|MGI=MGI=1915391|UniProtKB=Q8BGT5	ALAT2	Alanine aminotransferase 2;Gpt2;ortholog	ALANINE AMINOTRANSFERASE 2 (PTHR11751:SF311)	transaminase(PC00216)	Mus musculus
MOUSE|MGI=MGI=1921364|UniProtKB=Q9DC50	OCTC	Peroxisomal carnitine O-octanoyltransferase;Crot;ortholog	PEROXISOMAL CARNITINE O-OCTANOYLTRANSFERASE (PTHR22589:SF67)	acyltransferase(PC00042)	Mus musculus
MOUSE|MGI=MGI=1915077|UniProtKB=Q9EQ20	MMSA	Methylmalonate-semialdehyde dehydrogenase [acylating], mitochondrial;Aldh6a1;ortholog	METHYLMALONATE-SEMIALDEHYDE DEHYDROGENASE [ACYLATING], MITOCHONDRIAL (PTHR43866:SF3)	dehydrogenase(PC00092)	Mus musculus
MOUSE|MGI=MGI=1345167|UniProtKB=Q9WVM8	AADAT	Kynurenine/alpha-aminoadipate aminotransferase, mitochondrial;Aadat;ortholog	KYNURENINE/ALPHA-AMINOADIPATE AMINOTRANSFERASE, MITOCHONDRIAL (PTHR42790:SF3)	transaminase(PC00216)	Mus musculus
MOUSE|MGI=MGI=97402|UniProtKB=P00860	ODC	Ornithine decarboxylase;Odc1;ortholog	ORNITHINE DECARBOXYLASE (PTHR11482:SF42)	decarboxylase(PC00089)	Mus musculus
MOUSE|MGI=MGI=1346042|UniProtKB=O35678	MGLL	Monoglyceride lipase;Mgll;ortholog	MONOGLYCERIDE LIPASE (PTHR11614:SF87)	phospholipase(PC00186)	Mus musculus
MOUSE|MGI=MGI=103078|UniProtKB=Q64105	SPRE	Sepiapterin reductase;Spr;ortholog	SEPIAPTERIN REDUCTASE (PTHR44085:SF2)	reductase(PC00198)	Mus musculus
MOUSE|MGI=MGI=1098623|UniProtKB=Q8BWT1	THIM	3-ketoacyl-CoA thiolase, mitochondrial;Acaa2;ortholog	3-KETOACYL-COA THIOLASE, MITOCHONDRIAL (PTHR18919:SF107)	acyltransferase(PC00042)	Mus musculus
MOUSE|MGI=MGI=2136460|UniProtKB=Q8BH95	ECHM	Enoyl-CoA hydratase, mitochondrial;Echs1;ortholog	ENOYL-COA HYDRATASE, MITOCHONDRIAL (PTHR11941:SF54)	lyase(PC00144)	Mus musculus
MOUSE|MGI=MGI=1338800|UniProtKB=Q9Z0S1	BPNT1	3'(2'),5'-bisphosphate nucleotidase 1;Bpnt1;ortholog	3'(2'),5'-BISPHOSPHATE NUCLEOTIDASE 1 (PTHR43028:SF5)	phosphatase(PC00181)	Mus musculus
MOUSE|MGI=MGI=1922828|UniProtKB=A2AJL3	FGGY	FGGY carbohydrate kinase domain-containing protein;Fggy;ortholog	FGGY CARBOHYDRATE KINASE DOMAIN-CONTAINING PROTEIN (PTHR43435:SF4)	carbohydrate kinase(PC00065)	Mus musculus
MOUSE|MGI=MGI=1338011|UniProtKB=Q9JLZ3	AUHM	Methylglutaconyl-CoA hydratase, mitochondrial;Auh;ortholog	METHYLGLUTACONYL-COA HYDRATASE, MITOCHONDRIAL (PTHR11941:SF12)	lyase(PC00144)	Mus musculus
MOUSE|MGI=MGI=1330223|UniProtKB=O88428	PAPS2	Bifunctional 3'-phosphoadenosine 5'-phosphosulfate synthase 2;Papss2;ortholog	BIFUNCTIONAL 3'-PHOSPHOADENOSINE 5'-PHOSPHOSULFATE SYNTHASE 2 (PTHR11055:SF16)	kinase(PC00137);nucleotidyltransferase(PC00174)	Mus musculus
MOUSE|MGI=MGI=96112|UniProtKB=P22907	HEM3	Porphobilinogen deaminase;Hmbs;ortholog	PORPHOBILINOGEN DEAMINASE (PTHR11557:SF0)	deaminase(PC00088)	Mus musculus
MOUSE|MGI=MGI=88594|UniProtKB=Q9DBG1	CP27A	Sterol 26-hydroxylase, mitochondrial;Cyp27a1;ortholog	STEROL 26-HYDROXYLASE, MITOCHONDRIAL (PTHR24291:SF6)	oxygenase(PC00177)	Mus musculus
MOUSE|Gene=Ndufb1|UniProtKB=P0DN34	NDUB1	NADH dehydrogenase [ubiquinone] 1 beta subcomplex subunit 1;Ndufb1;ortholog	NADH DEHYDROGENASE [UBIQUINONE] 1 BETA SUBCOMPLEX SUBUNIT 1 (PTHR15222:SF2)	dehydrogenase(PC00092)	Mus musculus
MOUSE|MGI=MGI=102503|UniProtKB=P00405	COX2	Cytochrome c oxidase subunit 2;Mtco2;ortholog	CYTOCHROME C OXIDASE SUBUNIT 2 (PTHR22888:SF9)	oxidoreductase(PC00176)	Mus musculus
MOUSE|MGI=MGI=1914135|UniProtKB=Q9DBL1	ACDSB	Short/branched chain specific acyl-CoA dehydrogenase, mitochondrial;Acadsb;ortholog	SHORT/BRANCHED CHAIN SPECIFIC ACYL-COA DEHYDROGENASE, MITOCHONDRIAL (PTHR43884:SF1)	dehydrogenase(PC00092)	Mus musculus
MOUSE|MGI=MGI=1277989|UniProtKB=Q9CZN7	Q9CZN7	Serine hydroxymethyltransferase,	SERINE HYDROXYMETHYLTRANSFERASE,	methyltransferase(PC00155)	Mus musculus

		mitochondrial;Shmt2;ortholog	MITOCHONDRIAL (PTHR11680:SF28)		
MOUSE|MGI=MGI=88090|UniProtKB=P16460	ASSY	Argininosuccinate synthase;Ass1;ortholog	ARGININOSUCCINATE SYNTHASE (PTHR11587:SF2)	ligase(PC00142)	Mus musculus
MOUSE|MGI=MGI=97520|UniProtKB=Q05920	PYC	Pyruvate carboxylase, mitochondrial;Pc;ortholog	PYRUVATE CARBOXYLASE, MITOCHONDRIAL (PTHR43778:SF2)	ligase(PC00142)	Mus musculus
MOUSE|MGI=MGI=108388|UniProtKB=P97364	SPS2	Selenide, water dikinase 2;Sephs2;ortholog	SELENIDE, WATER DIKINASE 2 (PTHR10256:SF1)	transferase(PC00220)	Mus musculus
MOUSE|MGI=MGI=1921435|UniProtKB=Q9D6Y9	GLGB	1,4-alpha-glucan-branching enzyme;Gbe1;ortholog	1,4-ALPHA-GLUCAN-BRANCHING ENZYME (PTHR43651:SF3)	amylase(PC00048)	Mus musculus
MOUSE|MGI=MGI=88470|UniProtKB=O88587	COMT	Catechol O-methyltransferase;Comt;ortholog	CATECHOL O-METHYLTRANSFERASE (PTHR43836:SF3)	methyltransferase(PC00155)	Mus musculus
MOUSE|MGI=MGI=1929093|UniProtKB=Q8VCZ9	HYPDH	Hydroxyproline dehydrogenase;Prodh2;ortholog	HYDROXYPROLINE DEHYDROGENASE (PTHR13914:SF0)	oxidase(PC00175)	Mus musculus
MOUSE|MGI=MGI=1349919|UniProtKB=O09111	NDUBB	NADH dehydrogenase [ubiquinone] 1 beta subcomplex subunit 11, mitochondrial;Ndufb11;ortholog	NADH DEHYDROGENASE [UBIQUINONE] 1 BETA SUBCOMPLEX SUBUNIT 11, MITOCHONDRIAL (PTHR13327:SF1)	oxidoreductase(PC00176)	Mus musculus
MOUSE|MGI=MGI=2180167|UniProtKB=Q8K4Z3	NNRE	NAD(P)H-hydrate epimerase;Naxe;ortholog	NAD(P)H-HYDRATE EPIMERASE (PTHR13232:SF11)	epimerase/racemase(PC00096)	Mus musculus
MOUSE|MGI=MGI=1891372|UniProtKB=Q924Y0	BODG	Gamma-butyrobetaine dioxygenase;Bbox1;ortholog	GAMMA-BUTYROBETAINE DIOXYGENASE (PTHR10696:SF33)	hydroxylase(PC00122)	Mus musculus
MOUSE|MGI=MGI=88137|UniProtKB=Q6P3A8	ODBB	2-oxoisovalerate dehydrogenase subunit beta, mitochondrial;Bckdhb;ortholog	2-OXOISOVALERATE DEHYDROGENASE SUBUNIT BETA, MITOCHONDRIAL (PTHR42980:SF1)	dehydrogenase(PC00092)	Mus musculus
MOUSE|MGI=MGI=87921|UniProtKB=P00329	ADH1	Alcohol dehydrogenase 1;Adh1;ortholog	ALCOHOL DEHYDROGENASE 1C (PTHR43880:SF1)	dehydrogenase(PC00092)	Mus musculus
MOUSE|MGI=MGI=87870|UniProtKB=Q8QZT1	THIL	Acetyl-CoA acetyltransferase, mitochondrial;Acat1;ortholog	ACETYL-COA ACETYLTRANSFERASE, MITOCHONDRIAL (PTHR18919:SF156)	acyltransferase(PC00042)	Mus musculus
MOUSE|MGI=MGI=2183102|UniProtKB=Q99LB7	SARDH	Sarcosine dehydrogenase, mitochondrial;Sardh;ortholog	SARCOSINE DEHYDROGENASE, MITOCHONDRIAL (PTHR13847:SF200)	dehydrogenase(PC00092)	Mus musculus
MOUSE|MGI=MGI=2180098|UniProtKB=Q9DBE0	CSAD	Cysteine sulfinic acid decarboxylase;Csad;ortholog	CYSTEINE SULFINIC ACID DECARBOXYLASE (PTHR45677:SF7)	decarboxylase(PC00089)	Mus musculus
MOUSE|MGI=MGI=97501|UniProtKB=Q9Z2V4	PCKGC	Phosphoenolpyruvate carboxykinase, cytosolic [GTP];Pck1;ortholog	PHOSPHOENOLPYRUVATE CARBOXYKINASE, CYTOSOLIC [GTP] (PTHR11561:SF1)	kinase(PC00137)	Mus musculus
MOUSE|MGI=MGI=102797|UniProtKB=P41216	ACSL1	Long-chain-fatty-acid--CoA ligase 1;Acsl1;ortholog	LONG-CHAIN-FATTY-ACID--COA LIGASE 1 (PTHR43272:SF28)	ligase(PC00142)	Mus musculus
MOUSE|MGI=MGI=1915886|UniProtKB=Q8R0F8	FAHD1	Acylpyruvase FAHD1, mitochondrial;Fahd1;ortholog	ACYLPYRUVASE FAHD1, MITOCHONDRIAL (PTHR11820:SF7)	hydrolase(PC00121)	Mus musculus
MOUSE|MGI=MGI=1934852|UniProtKB=Q9QXD1	ACOX2	Peroxisomal acyl-coenzyme A oxidase 2;Acox2;ortholog	PEROXISOMAL ACYL-COENZYME A OXIDASE 2 (PTHR10909:SF344)	oxidoreductase(PC00176)	Mus musculus
MOUSE|MGI=MGI=1913402|UniProtKB=Q8R1I1	QCR9	Cytochrome b-c1 complex subunit 9;Uqcr10;ortholog	CYTOCHROME B-C1 COMPLEX SUBUNIT 9 (PTHR12980:SF0)	reductase(PC00198)	Mus musculus
MOUSE|MGI=MGI=87871|UniProtKB=Q8CAY6	THIC	Acetyl-CoA acetyltransferase, cytosolic;Acat2;ortholog	ACETYL-COA ACETYLTRANSFERASE, CYTOSOLIC (PTHR18919:SF81)	acyltransferase(PC00042)	Mus musculus
MOUSE|MGI=MGI=1923236|UniProtKB=A2AS89	SPEB	Agmatinase, mitochondrial;Agmat;ortholog	AGMATINASE, MITOCHONDRIAL (PTHR11358:SF26)	hydrolase(PC00121)	Mus musculus
MOUSE|MGI=MGI=2388287|UniProtKB=Q8VCW8	ACSF2	Acyl-CoA synthetase family member 2, mitochondrial;Acsf2;ortholog	ACYL-COA SYNTHETASE FAMILY MEMBER 2, MITOCHONDRIAL (PTHR43201:SF9)	ligase(PC00142)	Mus musculus
MOUSE|MGI=MGI=95530|UniProtKB=P97807	FUMH	Fumarate hydratase, mitochondrial;Fh;ortholog	FUMARATE HYDRATASE, MITOCHONDRIAL (PTHR11444:SF1)	lyase(PC00144)	Mus musculus
MOUSE|MGI=MGI=2385311|UniProtKB=Q8BMF4	ODP2	Dihydrolipoyllysine-residue acetyltransferase component of pyruvate dehydrogenase complex, mitochondrial;Dlat;ortholog	DIHYDROLIPOYLLYSINE-RESIDUE ACETYLTRANSFERASE COMPONENT OF PYRUVATE DEHYDROGENASE COMPLEX, MITOCHONDRIAL (PTHR23151:SF9)	acetyltransferase(PC00038)	Mus musculus
MOUSE|MGI=MGI=2442420|UniProtKB=Q8K157	GALM	Aldose 1-epimerase;Galm;ortholog	ALDOSE 1-EPIMERASE (PTHR10091:SF0)	epimerase/racemase(PC00096)	Mus musculus

MOUSE|MGI=MGI=1929955|UniProtKB=Q9JII6	AK1A1	Aldo-keto reductase family 1 member A1;Akr1a1;ortholog	ALDO-KETO REDUCTASE FAMILY 1 MEMBER A1 (PTHR11732:SF462)	reductase(PC00198)	Mus musculus
MOUSE|MGI=MGI=96213|UniProtKB=P49429	HPPD	4-hydroxyphenylpyruvate dioxygenase;Hpd;ortholog	4-HYDROXYPHENYLPYRUVATE DIOXYGENASE (PTHR11959:SF12)	oxygenase(PC00177)	Mus musculus
MOUSE|MGI=MGI=1096353|UniProtKB=P97328	KHK	Ketohexokinase;Khk;ortholog	KETOHEXOKINASE (PTHR43085:SF1)	carbohydrate kinase(PC00065)	Mus musculus
MOUSE|MGI=MGI=1919161|UniProtKB=Q80XN0	BDH	D-beta-hydroxybutyrate dehydrogenase, mitochondrial;Bdh1;ortholog	D-BETA-HYDROXYBUTYRATE DEHYDROGENASE, MITOCHONDRIAL (PTHR43313:SF25)	dehydrogenase(PC00092)	Mus musculus
MOUSE|MGI=MGI=1921379|UniProtKB=Q9DBT9	M2GD	Dimethylglycine dehydrogenase, mitochondrial;Dmgdh;ortholog	DIMETHYLGLYCINE DEHYDROGENASE, MITOCHONDRIAL (PTHR13847:SF187)	dehydrogenase(PC00092)	Mus musculus
MOUSE|MGI=MGI=1339962|UniProtKB=Q91XD4	FTCD	Formimidoyltransferase-cyclodeaminase;Ftcd;ortholog	FORMIMIDOYLTRANSFERASE-CYCLODEAMINASE (PTHR12234:SF0)	deaminase(PC00088);transferase(PC00220)	Mus musculus
MOUSE|MGI=MGI=97836|UniProtKB=Q8BVI4	DHPR	Dihydropteridine reductase;Qdpr;ortholog	DIHYDROPTERIDINE REDUCTASE (PTHR15104:SF0)	reductase(PC00198)	Mus musculus
MOUSE|MGI=MGI=1914166|UniProtKB=Q9CR61	NDUB7	NADH dehydrogenase [ubiquinone] 1 beta subcomplex subunit 7;Ndufb7;ortholog	NADH DEHYDROGENASE [UBIQUINONE] 1 BETA SUBCOMPLEX SUBUNIT 7 (PTHR20900:SF0)	dehydrogenase(PC00092)	Mus musculus
MOUSE|MGI=MGI=1922026|UniProtKB=Q91VM9	IPYR2	Inorganic pyrophosphatase 2, mitochondrial;Ppa2;ortholog	INORGANIC PYROPHOSPHATASE 2, MITOCHONDRIAL (PTHR10286:SF49)	pyrophosphatase(PC00196)	Mus musculus
MOUSE|MGI=MGI=95911|UniProtKB=P50171	DHB8	Estradiol 17-beta-dehydrogenase 8;Hsd17b8;ortholog	ESTRADIOL 17-BETA-DEHYDROGENASE 8 (PTHR42760:SF83)	oxidoreductase(PC00176)	Mus musculus
MOUSE|MGI=MGI=98266|UniProtKB=Q64442	DHSO	Sorbitol dehydrogenase;Sord;ortholog	SORBITOL DEHYDROGENASE (PTHR43161:SF9)	dehydrogenase(PC00092)	Mus musculus
MOUSE|MGI=MGI=109176|UniProtKB=P52825	CPT2	Carnitine O-palmitoyltransferase 2, mitochondrial;Cpt2;ortholog	CARNITINE O-PALMITOYLTRANSFERASE 2, MITOCHONDRIAL (PTHR22589:SF51)	acyltransferase(PC00042)	Mus musculus
MOUSE|MGI=MGI=107450|UniProtKB=O08749	DLDH	Dihydrolipoyl dehydrogenase, mitochondrial;Dld;ortholog	DIHYDROLIPOYL DEHYDROGENASE, MITOCHONDRIAL (PTHR22912:SF151)	oxidoreductase(PC00176)	Mus musculus
MOUSE|MGI=MGI=2148491|UniProtKB=Q921H8	THIKA	3-ketoacyl-CoA thiolase A, peroxisomal;Acaa1a;ortholog	3-KETOACYL-COA THIOLASE A, PEROXISOMAL (PTHR43853:SF19)	acetyltransferase(PC00038)	Mus musculus
MOUSE|MGI=MGI=107372|UniProtKB=Q60866	PTER	Phosphotriesterase-related protein;Pter;ortholog	PHOSPHOTRIESTERASE-RELATED PROTEIN (PTHR10819:SF3)	hydrolase(PC00121)	Mus musculus
MOUSE|MGI=MGI=109279|UniProtKB=Q61941	NNTM	NAD(P) transhydrogenase, mitochondrial;Nnt;ortholog	NAD(P) TRANSHYDROGENASE, MITOCHONDRIAL (PTHR10160:SF22)	dehydrogenase(PC00092)	Mus musculus
MOUSE|MGI=MGI=1920296|UniProtKB=Q80Y14	GLRX5	Glutaredoxin-related protein 5, mitochondrial;Glrx5;ortholog	GLUTAREDOXIN-RELATED PROTEIN 5, MITOCHONDRIAL (PTHR10293:SF16)	reductase(PC00198)	Mus musculus
MOUSE|MGI=MGI=94873|UniProtKB=P61458	PHS	Pterin-4-alpha-carbinolamine dehydratase;Pcbd1;ortholog	PTERIN-4-ALPHA-CARBINOLAMINE DEHYDRATASE (PTHR12599:SF13)	dehydratase(PC00091)	Mus musculus
MOUSE|MGI=MGI=98731|UniProtKB=P21981	TGM2	Protein-glutamine gamma-glutamyltransferase 2;Tgm2;ortholog	PROTEIN-GLUTAMINE GAMMA-GLUTAMYLTRANSFERASE 2 (PTHR11590:SF6)	transferase(PC00220)	Mus musculus
MOUSE|MGI=MGI=2178759|UniProtKB=Q924D0	RT4I1	Reticulon-4-interacting protein 1, mitochondrial;Rtn4ip1;ortholog	RETICULON-4-INTERACTING PROTEIN 1, MITOCHONDRIAL (PTHR11695:SF294)	dehydrogenase(PC00092)	Mus musculus
MOUSE|MGI=MGI=1913358|UniProtKB=Q9DC69	NDUA9	NADH dehydrogenase [ubiquinone] 1 alpha subcomplex subunit 9, mitochondrial;Ndufa9;ortholog	NADH DEHYDROGENASE [UBIQUINONE] 1 ALPHA SUBCOMPLEX SUBUNIT 9, MITOCHONDRIAL (PTHR12126:SF11)	dehydrogenase(PC00092);reductase(PC00198)	Mus musculus
MOUSE|MGI=MGI=2441982|UniProtKB=Q8BWF0	SSDH	Succinate-semialdehyde dehydrogenase, mitochondrial;Aldh5a1;ortholog	SUCCINATE-SEMIALDEHYDE DEHYDROGENASE, MITOCHONDRIAL (PTHR43353:SF5)	dehydrogenase(PC00092)	Mus musculus
MOUSE|MGI=MGI=1349419|UniProtKB=Q9Z0X1	AIFM1	Apoptosis-inducing factor 1, mitochondrial;Aifm1;ortholog	APOPTOSIS-INDUCING FACTOR 1, MITOCHONDRIAL (PTHR43557:SF4)	oxidoreductase(PC00176)	Mus musculus
MOUSE|MGI=MGI=103099|UniProtKB=P43024	CX6A1	Cytochrome c oxidase subunit 6A1, mitochondrial;Cox6a1;ortholog	CYTOCHROME C OXIDASE SUBUNIT 6A1, MITOCHONDRIAL (PTHR11504:SF4)	oxidase(PC00175)	Mus musculus

MOUSE|MGI=MGI=105386|UniProtKB=P53395	ODB2	Lipoamide acyltransferase component of branched-chain alpha-keto acid dehydrogenase complex, mitochondrial;Dbt;ortholog	LIPOAMIDE ACYLTRANSFERASE COMPONENT OF BRANCHED-CHAIN ALPHA-KETO ACID DEHYDROGENASE COMPLEX, MITOCHONDRIAL (PTHR43178:SF5)	acetyltransferase(PC00038)	Mus musculus
MOUSE|MGI=MGI=1918974|UniProtKB=G3X982	AOXC	Aldehyde oxidase 3;Aox3;ortholog	ALDEHYDE OXIDASE 3 (PTHR11908:SF99)	oxidoreductase(PC00176)	Mus musculus
MOUSE|MGI=MGI=87880|UniProtKB=Q99KI0	ACON	Aconitate hydratase, mitochondrial;Aco2;ortholog	ACONITATE HYDRATASE, MITOCHONDRIAL (PTHR43160:SF3)	hydratase(PC00120)	Mus musculus
MOUSE|MGI=MGI=2140356|UniProtKB=Q8CFX1	G6PE	GDH/6PGL endoplasmic bifunctional protein;H6pd;ortholog	GDH/6PGL ENDOPLASMIC BIFUNCTIONAL PROTEIN (PTHR23429:SF7)	dehydrogenase(PC00092)	Mus musculus
MOUSE|MGI=MGI=1339968|UniProtKB=Q8VCN5	CGL	Cystathionine gamma-lyase;Cth;ortholog	CYSTATHIONINE GAMMA-LYASE (PTHR11808:SF15)	lyase(PC00144)	Mus musculus
MOUSE|MGI=MGI=1922725|UniProtKB=Q8K010	OPLA	5-oxoprolinase;Oplah;ortholog	5-OXOPROLINASE (PTHR11365:SF2)	hydrolase(PC00121)	Mus musculus
MOUSE|MGI=MGI=1915592|UniProtKB=Q9DCS9	NDUBA	NADH dehydrogenase [ubiquinone] 1 beta subcomplex subunit 10;Ndufb10;ortholog	NADH DEHYDROGENASE [UBIQUINONE] 1 BETA SUBCOMPLEX SUBUNIT 10 (PTHR13094:SF1)	oxidoreductase(PC00176)	Mus musculus
MOUSE|MGI=MGI=87979|UniProtKB=Q9WUR9	KAD4	Adenylate kinase 4, mitochondrial;Ak4;ortholog	ADENYLATE KINASE 4, MITOCHONDRIAL (PTHR23359:SF58)	nucleotide kinase(PC00172)	Mus musculus
MOUSE|MGI=MGI=1351661|UniProtKB=Q9JLI0	Q9JLI0	Aldo-keto reductase a;Akr1c12;ortholog	ALDO-KETO REDUCTASE A-RELATED (PTHR11732:SF286)	reductase(PC00198)	Mus musculus
MOUSE|MGI=MGI=2148202|UniProtKB=Q8VCT4	CES1D	Carboxylesterase 1D;Ces1d;ortholog	LIVER CARBOXYLESTERASE 1-RELATED (PTHR11559:SF179)	esterase(PC00097)	Mus musculus
MOUSE|MGI=MGI=107932|UniProtKB=P52503	NDUS6	NADH dehydrogenase [ubiquinone] iron-sulfur protein 6, mitochondrial;Ndufs6;ortholog	NADH DEHYDROGENASE [UBIQUINONE] IRON-SULFUR PROTEIN 6, MITOCHONDRIAL (PTHR13156:SF0)	oxidoreductase(PC00176)	Mus musculus
MOUSE|MGI=MGI=1914710|UniProtKB=Q9CQ62	DECR	2,4-dienoyl-CoA reductase, mitochondrial;Decr1;ortholog	17-BETA-HYDROXYSTEROID DEHYDROGENASE 14-RELATED (PTHR43658:SF8)	oxidoreductase(PC00176)	Mus musculus
MOUSE|MGI=MGI=1098296|UniProtKB=P97742	CPT1A	Carnitine O-palmitoyltransferase 1, liver isoform;Cpt1a;ortholog	CARNITINE O-PALMITOYLTRANSFERASE 1, LIVER ISOFORM (PTHR22589:SF74)	acyltransferase(PC00042)	Mus musculus
MOUSE|MGI=MGI=891996|UniProtKB=Q8C196	CPSM	Carbamoyl-phosphate synthase [ammonia], mitochondrial;Cps1;ortholog	CARBAMOYL-PHOSPHATE SYNTHASE [AMMONIA], MITOCHONDRIAL (PTHR11405:SF38)	ligase(PC00142)	Mus musculus
MOUSE|MGI=MGI=98351|UniProtKB=P08228	SODC	Superoxide dismutase [Cu-Zn];Sod1;ortholog	SUPEROXIDE DISMUTASE [CU-ZN] (PTHR10003:SF66)	oxidoreductase(PC00176)	Mus musculus
MOUSE|MGI=MGI=2384785|UniProtKB=Q8VCX1	AK1D1	3-oxo-5-beta-steroid 4-dehydrogenase;Akr1d1;ortholog	3-OXO-5-BETA-STEROID 4-DEHYDROGENASE (PTHR11732:SF211)	reductase(PC00198)	Mus musculus
MOUSE|MGI=MGI=1914272|UniProtKB=Q8JZN5	ACAD9	Acyl-CoA dehydrogenase family member 9, mitochondrial;Acad9;ortholog	ACYL-COA DEHYDROGENASE FAMILY MEMBER 9, MITOCHONDRIAL (PTHR43884:SF9)	dehydrogenase(PC00092)	Mus musculus
MOUSE|MGI=MGI=1915008|UniProtKB=Q99PG0	AAAD	Arylacetamide deacetylase;Aadac;ortholog	ARYLACETAMIDE DEACETYLASE (PTHR23024:SF222)	deacetylase(PC00087)	Mus musculus
MOUSE|MGI=MGI=1926144|UniProtKB=Q9D2R0	AACS	Acetoacetyl-CoA synthetase;Aacs;ortholog	ACETOACETYL-COA SYNTHETASE (PTHR42921:SF1)	ligase(PC00142)	Mus musculus
MOUSE|MGI=MGI=98299|UniProtKB=P50431	GLYC	Serine hydroxymethyltransferase, cytosolic;Shmt1;ortholog	SERINE HYDROXYMETHYLTRANSFERASE, CYTOSOLIC (PTHR11680:SF21)	methyltransferase(PC00155)	Mus musculus
MOUSE|MGI=MGI=3646700|UniProtKB=Q8CFA2	GCST	Aminomethyltransferase, mitochondrial;Amt;ortholog	AMINOMETHYLTRANSFERASE, MITOCHONDRIAL (PTHR43757:SF2)	methyltransferase(PC00155)	Mus musculus
MOUSE|MGI=MGI=88474|UniProtKB=P12787	COX5A	Cytochrome c oxidase subunit 5A, mitochondrial;Cox5a;ortholog	CYTOCHROME C OXIDASE SUBUNIT 5A, MITOCHONDRIAL (PTHR14200:SF11)	oxidase(PC00175)	Mus musculus
MOUSE|MGI=MGI=1915444|UniProtKB=Q9CQC7	NDUB4	NADH dehydrogenase [ubiquinone] 1 beta subcomplex subunit 4;Ndufb4;ortholog	NADH DEHYDROGENASE [UBIQUINONE] 1 BETA SUBCOMPLEX SUBUNIT 4 (PTHR15469:SF0)	reductase(PC00198)	Mus musculus

MOUSE|MGI=MGI=1330812|UniProtKB=Q9R0H0	ACOX1	Peroxisomal acyl-coenzyme A oxidase 1;Acox1;ortholog	PEROXISOMAL ACYL-COENZYME A OXIDASE 1 (PTHR10909:SF383)	oxidoreductase(PC00176)	Mus musculus
MOUSE|MGI=MGI=88529|UniProtKB=Q9CZU6	CISY	Citrate synthase, mitochondrial;Cs;ortholog	CITRATE SYNTHASE, MITOCHONDRIAL (PTHR11739:SF8)	transferase(PC00220)	Mus musculus
MOUSE|MGI=MGI=1923513|UniProtKB=Q9DCM2	GSTK1	Glutathione S-transferase kappa 1;Gstk1;ortholog	GLUTATHIONE S-TRANSFERASE KAPPA 1 (PTHR42943:SF2)	transferase(PC00220)	Mus musculus
MOUSE|MGI=MGI=104645|UniProtKB=Q61694	3BHS5	NADPH-dependent 3-keto-steroid reductase Hsd3b5;Hsd3b5;ortholog	NADPH-DEPENDENT 3-KETO-STEROID REDUCTASE HSD3B4-RELATED (PTHR10366:SF297)	dehydratase(PC00091)	Mus musculus
MOUSE|MGI=MGI=1858208|UniProtKB=O35459	ECH1	Delta(3,5)-Delta(2,4)-dienoyl-CoA isomerase, mitochondrial;Ech1;ortholog	DELTA(3,5)-DELTA(2,4)-DIENOYL-COA ISOMERASE, MITOCHONDRIAL (PTHR43149:SF1)	hydratase(PC00120)	Mus musculus
MOUSE|MGI=MGI=1889802|UniProtKB=Q99L13	3HIDH	3-hydroxyisobutyrate dehydrogenase, mitochondrial;Hibadh;ortholog	3-HYDROXYISOBUTYRATE DEHYDROGENASE, MITOCHONDRIAL (PTHR22981:SF7)	dehydrogenase(PC00092)	Mus musculus
MOUSE|MGI=MGI=96853|UniProtKB=P10518	HEM2	Delta-aminolevulinic acid dehydratase;Alad;ortholog	DELTA-AMINOLEVULINIC ACID DEHYDRATASE (PTHR11458:SF0)	dehydratase(PC00091)	Mus musculus
MOUSE|MGI=MGI=1289238|UniProtKB=Q3TLP5	ECHD2	Enoyl-CoA hydratase domain-containing protein 2, mitochondrial;Echdc2;ortholog	ENOYL-COA HYDRATASE DOMAIN-CONTAINING PROTEIN 2, MITOCHONDRIAL (PTHR11941:SF44)	lyase(PC00144)	Mus musculus
MOUSE|MGI=MGI=5804923|UniProtKB=A0A1B0GSH8	A0A1B0GSH8	Predicted gene 45808 (Fragment);Gm45808;ortholog	TRANS-1,2-DIHYDROBENZENE-1,2-DIOL DEHYDROGENASE (PTHR22604:SF105)	dehydrogenase(PC00092)	Mus musculus
MOUSE|MGI=MGI=1860776|UniProtKB=Q8BJ64	CHDH	Choline dehydrogenase, mitochondrial;Chdh;ortholog	CHOLINE DEHYDROGENASE, MITOCHONDRIAL (PTHR11552:SF147)	dehydrogenase(PC00092)	Mus musculus
MOUSE|MGI=MGI=108024|UniProtKB=Q64374	RGN	Regucalcin;Rgn;ortholog	REGUCALCIN (PTHR10907:SF54)	esterase(PC00097)	Mus musculus
MOUSE|MGI=MGI=1929468|UniProtKB=P97493	THIOM	Thioredoxin, mitochondrial;Txn2;ortholog	THIOREDOXIN, MITOCHONDRIAL (PTHR43601:SF3)	oxidoreductase(PC00176)	Mus musculus
MOUSE|MGI=MGI=2139667|UniProtKB=Q8CHR6	DPYD	Dihydropyrimidine dehydrogenase [NADP(+)];Dpyd;ortholog	DIHYDROPYRIMIDINE DEHYDROGENASE [NADP(+)] (PTHR43073:SF2)	dehydrogenase(PC00092)	Mus musculus
MOUSE|MGI=MGI=1918039|UniProtKB=Q9CXF0	KYNU	Kynureninase;Kynu;ortholog	KYNURENINASE (PTHR14084:SF0)	hydrolase(PC00121)	Mus musculus
MOUSE|MGI=MGI=1343135|UniProtKB=Q9CXZ1	NDUS4	NADH dehydrogenase [ubiquinone] iron-sulfur protein 4, mitochondrial;Ndufs4;ortholog	NADH DEHYDROGENASE [UBIQUINONE] IRON-SULFUR PROTEIN 4, MITOCHONDRIAL (PTHR12219:SF8)	oxidoreductase(PC00176)	Mus musculus
MOUSE|MGI=MGI=3845761|UniProtKB=Q76I26	Q76I26	Methyltransferase hypoxia-inducible domain-containing 1;Methig1;ortholog	METHYLTRANSFERASE-LIKE PROTEIN 7A (PTHR42912:SF21)	methyltransferase(PC00155)	Mus musculus
MOUSE|MGI=MGI=88475|UniProtKB=P19536	COX5B	Cytochrome c oxidase subunit 5B, mitochondrial;Cox5b;ortholog	CYTOCHROME C OXIDASE SUBUNIT 5B, MITOCHONDRIAL (PTHR10122:SF0)	oxidase(PC00175)	Mus musculus
MOUSE|MGI=MGI=2385271|UniProtKB=Q923D2	BLVRB	Flavin reductase (NADPH);Blvrb;ortholog	FLAVIN REDUCTASE (NADPH) (PTHR43355:SF2)	reductase(PC00198)	Mus musculus
MOUSE|MGI=MGI=106092|UniProtKB=Q99LC5	ETFA	Electron transfer flavoprotein subunit alpha, mitochondrial;Etfa;ortholog	ELECTRON TRANSFER FLAVOPROTEIN SUBUNIT ALPHA, MITOCHONDRIAL (PTHR43153:SF1)	oxidoreductase(PC00176)	Mus musculus
MOUSE|MGI=MGI=2179733|UniProtKB=Q99J99	THTM	3-mercaptopyruvate sulfurtransferase;Mpst;ortholog	3-MERCAPTOPYRUVATE SULFURTRANSFERASE (PTHR11364:SF25)	transferase(PC00220)	Mus musculus
MOUSE|MGI=MGI=1341155|UniProtKB=Q91W43	GCSP	Glycine dehydrogenase (decarboxylating), mitochondrial;Gldc;ortholog	GLYCINE DEHYDROGENASE (DECARBOXYLATING), MITOCHONDRIAL (PTHR11773:SF1)	dehydrogenase(PC00092)	Mus musculus
MOUSE|MGI=MGI=1915408|UniProtKB=Q9CY27	TECR	Very-long-chain enoyl-CoA reductase;Tecr;ortholog	VERY-LONG-CHAIN ENOYL-COA REDUCTASE (PTHR10556:SF31)	dehydrogenase(PC00092)	Mus musculus
MOUSE|MGI=MGI=1913677|UniProtKB=Q9CQX2	CYB5B	Cytochrome b5 type B;Cyb5b;ortholog	CYTOCHROME B5 TYPE B (PTHR19359:SF14)	oxidoreductase(PC00176)	Mus musculus
MOUSE|MGI=MGI=101787|UniProtKB=P03888	NU1M	NADH-ubiquinone oxidoreductase chain 1;Mtnd1;ortholog	NADH-UBIQUINONE OXIDOREDUCTASE CHAIN 1 (PTHR11432:SF3)	dehydrogenase(PC00092)	Mus musculus

MOUSE|MGI=MGI=94871|UniProtKB=P42125	ECI1	Enoyl-CoA delta isomerase 1, mitochondrial;Eci1;ortholog	ENOYL-COA DELTA ISOMERASE 1, MITOCHONDRIAL (PTHR11941:SF45)	lyase(PC00144)	Mus musculus
MOUSE|MGI=MGI=1919785|UniProtKB=Q9CZS1	AL1B1	Aldehyde dehydrogenase X, mitochondrial;Aldh1b1;ortholog	ALDEHYDE DEHYDROGENASE X, MITOCHONDRIAL (PTHR11699:SF207)	dehydrogenase(PC00092)	Mus musculus
MOUSE|MGI=MGI=2143539|UniProtKB=Q571F8	GLSL	Glutaminase liver isoform, mitochondrial;Gls2;ortholog	GLUTAMINASE LIVER ISOFORM, MITOCHONDRIAL (PTHR12544:SF33)	hydrolase(PC00121)	Mus musculus
MOUSE|MGI=MGI=1926170|UniProtKB=Q9D2G2	ODO2	Dihydrolipoyllysine-residue succinyltransferase component of 2-oxoglutarate dehydrogenase complex, mitochondrial;Dlst;ortholog	DIHYDROLIPOYLLYSINE-RESIDUE SUCCINYLTRANSFERASE COMPONENT OF 2-OXOGLUTARATE DEHYDROGENASE COMPLEX, MITOCHONDRIAL (PTHR43416:SF5)	transferase(PC00220)	Mus musculus
MOUSE|MGI=MGI=2148199|UniProtKB=Q99MZ7	PECR	Peroxisomal trans-2-enoyl-CoA reductase;Pecr;ortholog	PEROXISOMAL TRANS-2-ENOYL-COA REDUCTASE (PTHR24317:SF7)	reductase(PC00198)	Mus musculus
MOUSE|MGI=MGI=2142687|UniProtKB=Q91WU0	CES1F	Carboxylesterase 1F;Ces1f;ortholog	CARBOXYLESTERASE 1F-RELATED (PTHR11559:SF181)	esterase(PC00097)	Mus musculus
MOUSE|MGI=MGI=2144151|UniProtKB=Q91XF0	PNPO	Pyridoxine-5'-phosphate oxidase;Pnpo;ortholog	PYRIDOXINE-5'-PHOSPHATE OXIDASE (PTHR10851:SF0)	oxidase(PC00175)	Mus musculus
MOUSE|MGI=MGI=1925288|UniProtKB=Q3ULD5	MCCB	Methylcrotonoyl-CoA carboxylase beta chain, mitochondrial;Mccc2;ortholog	METHYLCROTONOYL-COA CARBOXYLASE BETA CHAIN, MITOCHONDRIAL (PTHR22855:SF13)	ligase(PC00142)	Mus musculus
MOUSE|MGI=MGI=102963|UniProtKB=P40936	INMT	Indolethylamine N-methyltransferase;Inmt;ortholog	INDOLETHYLAMINE N-METHYLTRANSFERASE (PTHR10867:SF33)	methyltransferase(PC00155)	Mus musculus
MOUSE|MGI=MGI=1344370|UniProtKB=Q9CQ54	NDUC2	NADH dehydrogenase [ubiquinone] 1 subunit C2;Ndufc2;ortholog	NADH DEHYDROGENASE [UBIQUINONE] 1 SUBUNIT C2-RELATED (PTHR13099:SF0)	oxidoreductase(PC00176)	Mus musculus
MOUSE|MGI=MGI=1914434|UniProtKB=Q9ERS2	NDUAD	NADH dehydrogenase [ubiquinone] 1 alpha subcomplex subunit 13;Ndufa13;ortholog	NADH DEHYDROGENASE [UBIQUINONE] 1 ALPHA SUBCOMPLEX SUBUNIT 13 (PTHR12966:SF0)	dehydrogenase(PC00092)	Mus musculus
MOUSE|MGI=MGI=1919129|UniProtKB=Q8JZR0	ACSL5	Long-chain-fatty-acid--CoA ligase 5;Acsl5;ortholog	LONG-CHAIN-FATTY-ACID--COA LIGASE 5 (PTHR43272:SF33)	ligase(PC00142)	Mus musculus
MOUSE|MGI=MGI=1923792|UniProtKB=Q8QZS1	HIBCH	3-hydroxyisobutyryl-CoA hydrolase, mitochondrial;Hibch;ortholog	3-HYDROXYISOBUTYRYL-COA HYDROLASE, MITOCHONDRIAL (PTHR43176:SF3)	hydrolase(PC00121)	Mus musculus
MOUSE|MGI=MGI=1919812|UniProtKB=Q9CZL5	PHS2	Pterin-4-alpha-carbinolamine dehydratase 2;Pcbd2;ortholog	PTERIN-4-ALPHA-CARBINOLAMINE DEHYDRATASE 2 (PTHR12599:SF15)	dehydratase(PC00091)	Mus musculus
MOUSE|MGI=MGI=1349472|UniProtKB=Q9QYY9	ADH4	Alcohol dehydrogenase 4;Adh4;ortholog	ALCOHOL DEHYDROGENASE 4 (PTHR43880:SF14)	dehydrogenase(PC00092)	Mus musculus
MOUSE|MGI=MGI=106594|UniProtKB=Q64516	GLPK	Glycerol kinase;Gk;ortholog	GLYCEROL KINASE (PTHR10196:SF56)	carbohydrate kinase(PC00065)	Mus musculus
MOUSE|MGI=MGI=1914682|UniProtKB=Q9DCU9	HOGA1	4-hydroxy-2-oxoglutarate aldolase, mitochondrial;Hoga1;ortholog	4-HYDROXY-2-OXOGLUTARATE ALDOLASE, MITOCHONDRIAL (PTHR12128:SF15)	lyase(PC00144)	Mus musculus
MOUSE|MGI=MGI=1316706|UniProtKB=Q9Z1J3	NFS1	Cysteine desulfurase, mitochondrial;Nfs1;ortholog	CYSTEINE DESULFURASE, MITOCHONDRIAL (PTHR11601:SF34)	lyase(PC00144)	Mus musculus
MOUSE|MGI=MGI=1914780|UniProtKB=Q9D855	QCR7	Cytochrome b-c1 complex subunit 7;Uqcrb;ortholog	CYTOCHROME B-C1 COMPLEX SUBUNIT 7 (PTHR12022:SF0)	reductase(PC00198)	Mus musculus
MOUSE|MGI=MGI=1353450|UniProtKB=P24549	AL1A1	Retinal dehydrogenase 1;Aldh1a1;ortholog	RETINAL DEHYDROGENASE 1 (PTHR11699:SF140)	dehydrogenase(PC00092)	Mus musculus

Table S2. List of proteins down-regulated in Kupffer cells vs monocytes and belonged to PC00171 group of proteins (nucleic acid binding protein) according to PANTHER (http://pantherdb.org/).

Gene ID	Mapped IDs	Gene Name, Gene symbol, ortholog	PANTHER Family/Subfamily	PANTHER Protein Class	Species
MOUSE|MGI=MGI=1916231|UniProtKB=P57784	RU2A	U2 small nuclear ribonucleoprotein	U2 SMALL NUCLEAR RIBONUCLEOPROT	RNA splicing factor(PC00148)	Mus musculu

		A';Snrpa1;ortholog	EIN A' (PTHR10552:SF6)		s
MOUSE|MGI=MGI=1923772|UniProtKB=Q6ZWM4	LSM8	U6 snRNA-associated Sm-like protein LSm8;Lsm8;ortholog	U6 SNRNA-ASSOCIATED SM-LIKE PROTEIN LSM8 (PTHR15588:SF9)	RNA splicing factor(PC00148)	Mus musculus
MOUSE|MGI=MGI=2679722|UniProtKB=Q9EPU4	CPSF1	Cleavage and polyadenylation specificity factor subunit 1;Cpsf1;ortholog	CLEAVAGE AND POLYADENYLATION SPECIFICITY FACTOR SUBUNIT 1 (PTHR10644:SF2)	RNA processing factor(PC00147)	Mus musculus
MOUSE|MGI=MGI=1926421|UniProtKB=Q8CGF7	TCRG1	Transcription elongation regulator 1;Tcerg1;ortholog	TRANSCRIPTION ELONGATION REGULATOR 1 (PTHR15377:SF7)	general transcription factor(PC00259)	Mus musculus
MOUSE|MGI=MGI=99894|UniProtKB=P61979	HNRPK	Heterogeneous nuclear ribonucleoprotein K;Hnrnpk;ortholog	HETEROGENEOUS NUCLEAR RIBONUCLEOPROTEIN K (PTHR10288:SF179)	RNA binding protein(PC00031)	Mus musculus
MOUSE|MGI=MGI=1095403|UniProtKB=Q64213	SF01	Splicing factor 1;Sf1;ortholog	SPLICING FACTOR 1 (PTHR11208:SF45)	RNA splicing factor(PC00148)	Mus musculus
MOUSE|MGI=MGI=1913895|UniProtKB=Q8R326	PSPC1	Paraspeckle component 1;Pspc1;ortholog	PARASPECKLE COMPONENT 1 (PTHR23189:SF14)	RNA binding protein(PC00031)	Mus musculus
MOUSE|MGI=MGI=892003|UniProtKB=O08784	TCOF	Treacle protein;Tcof1;ortholog	TREACLE PROTEIN (PTHR20787:SF10)	RNA binding protein(PC00031)	Mus musculus
MOUSE|MGI=MGI=1914120|UniProtKB=Q9CY58	PAIRB	Plasminogen activator inhibitor 1 RNA-binding protein;Serbp1;ortholog	PLASMINOGEN ACTIVATOR INHIBITOR 1 RNA-BINDING PROTEIN (PTHR12299:SF29)	RNA binding protein(PC00031)	Mus musculus
MOUSE|MGI=MGI=1913670|UniProtKB=Q80UW8	RPAB1	DNA-directed RNA polymerases I, II, and III subunit RPABC1;Polr2e;ortholog	DNA-DIRECTED RNA POLYMERASES I, II, AND III SUBUNIT RPABC1 (PTHR10535:SF0)	DNA-directed RNA polymerase(PC00019)	Mus musculus
MOUSE|MGI=MGI=1932339|UniProtKB=Q99NB9	SF3B1	Splicing factor 3B subunit 1;Sf3b1;ortholog	SPLICING FACTOR 3B SUBUNIT 1 (PTHR12097:SF0)	RNA splicing factor(PC00148)	Mus musculus
MOUSE|MGI=MGI=98345|UniProtKB=P62317	SMD2	Small nuclear ribonucleoprotein Sm D2;Snrpd2;ortholog	SMALL NUCLEAR RIBONUCLEOPROTEIN SM D2 (PTHR12777:SF0)	RNA processing factor(PC00147)	Mus musculus
MOUSE|MGI=MGI=1346087|UniProtKB=P14576	SRP54	Signal recognition particle 54 kDa protein;Srp54;ortholog	SIGNAL RECOGNITION PARTICLE 54 KDA PROTEIN (PTHR11564:SF5)	RNA binding protein(PC00031)	Mus musculus
MOUSE|MGI=MGI=96160|UniProtKB=P17095	HMGA1	High mobility group protein HMG-I/HMG-Y;Hmga1;ortholog	HIGH MOBILITY GROUP PROTEIN HMG-I/HMG-Y (PTHR23341:SF1)	endodeoxyribonuclease(PC00093)	Mus musculus
MOUSE|MGI=MGI=2446249|UniProtKB=Q3UJB9	EDC4	Enhancer of mRNA-decapping protein 4;Edc4;ortholog	ENHANCER OF MRNA-DECAPPING PROTEIN 4 (PTHR15598:SF5)	mRNA capping factor(PC00145)	Mus musculus
MOUSE|MGI=MGI=104912|UniProtKB=Q62203	SF3A2	Splicing factor 3A subunit 2;Sf3a2;ortholog	SPLICING FACTOR 3A SUBUNIT 2 (PTHR23205:SF0)	RNA splicing factor(PC00148)	Mus musculus
MOUSE|MGI=MGI=1926232|UniProtKB=Q8BL97	SRSF7	Serine/arginine-rich splicing factor 7;Srsf7;ortholog	SERINE/ARGININE-RICH SPLICING FACTOR 7 (PTHR23147:SF18)	RNA splicing factor(PC00148)	Mus musculus
MOUSE|MGI=MGI=1931527|UniProtKB=P43274	H14	Histone H1.4;Hist1h1e;ortholog	HISTONE H1.4 (PTHR11467:SF57)	histone(PC00118)	Mus musculus
MOUSE|MGI=MGI=94912|UniProtKB=P13864	DNMT1	DNA (cytosine-5)-methyltransferase 1;Dnmt1;ortholog	DNA (CYTOSINE-5)-METHYLTRANSFERASE 1 (PTHR10629:SF52)	DNA methyltransferase(PC00013)	Mus musculus
MOUSE|MGI=MGI=99918|UniProtKB=Q9Z2D6	MECP2	Methyl-CpG-binding protein 2;Mecp2;ortholog	METHYL-CPG-BINDING DOMAIN PROTEIN 4-RELATED (PTHR15074:SF0)	DNA binding protein(PC00009)	Mus musculus
MOUSE|MGI=MGI=1919912|UniProtKB=Q9CSH3	RRP44	Exosome complex exonuclease RRP44;Dis3;ortholog	EXOSOME COMPLEX EXONUCLEASE RRP44 (PTHR23355:SF35)	exoribonuclease(PC00099)	Mus musculus
MOUSE|MGI=MGI=1923576|UniProtKB=Q921I9	EXOS4	Exosome complex component RRP41;Exosc4;ortholog	EXOSOME COMPLEX COMPONENT RRP41 (PTHR11953:SF0)	exoribonuclease(PC00099)	Mus musculus
MOUSE|MGI=MGI=2442402|UniProtKB=Q6ZQ08	CNOT1	CCR4-NOT transcription complex subunit 1;Cnot1;ortholog	CCR4-NOT TRANSCRIPTION COMPLEX SUBUNIT 1 (PTHR13162:SF8)	mRNA polyadenylation factor(PC00146)	Mus musculus
MOUSE|MGI=MGI=1858234|UniProtKB=Q60865	CAPR1	Caprin-1;Caprin1;ortholog	CAPRIN-1 (PTHR22922:SF3)	RNA binding protein(PC00031)	Mus musculus
MOUSE|MGI=MGI=893579|UniProtKB=Q60749	KHDR1	KH domain-containing, RNA-binding, signal transduction-associated protein 1;Khdrbs1;ortholog	KH DOMAIN-CONTAINING, RNA-BINDING, SIGNAL TRANSDUCTION-ASSOCIATED PROTEIN 1 (PTHR11208:SF30)	RNA splicing factor(PC00148)	Mus musculus
MOUSE|MGI=MGI=98283|UniProtKB=Q6PDM2	SRSF1	Serine/arginine-rich splicing factor	SERINE/ARGININE-RICH SPLICING	RNA splicing factor(PC00148)	Mus musculu

		1;Srsf1;ortholog	FACTOR 1 (PTHR23147:SF44)		s
MOUSE|MGI=MGI=894687|UniProtKB=Q9DBR1	XRN2	5'-3' exoribonuclease 2;Xrn2;ortholog	5'-3' EXORIBONUCLEASE 2 (PTHR12341:SF41)	exoribonuclease(PC00099)	Mus musculus
MOUSE|MGI=MGI=103199|UniProtKB=P49717	MCM4	DNA replication licensing factor MCM4;Mcm4;ortholog	DNA REPLICATION LICENSING FACTOR MCM4 (PTHR11630:SF66)	DNA binding protein(PC00009)	Mus musculus
MOUSE|MGI=MGI=1196624|UniProtKB=P10711	TCEA1	Transcription elongation factor A protein 1;Tcea1;ortholog	TRANSCRIPTION ELONGATION FACTOR A PROTEIN 1 (PTHR11477:SF1)	general transcription factor(PC00259)	Mus musculus
MOUSE|MGI=MGI=107252|UniProtKB=Q1HFZ0	NSUN2	tRNA (cytosine(34)-C(5))-methyltransferase;Nsun2;ortholog	TRNA (CYTOSINE(34)-C(5))-METHYLTRANSFERASE (PTHR22808:SF20)	RNA methyltransferase(PC00033)	Mus musculus
MOUSE|MGI=MGI=90676|UniProtKB=O35900	LSM2	U6 snRNA-associated Sm-like protein LSm2;Lsm2;ortholog	U6 SNRNA-ASSOCIATED SM-LIKE PROTEIN LSM2 (PTHR13829:SF2)	RNA splicing factor(PC00148)	Mus musculus
MOUSE|MGI=MGI=97960|UniProtKB=Q99M28	RNPS1	RNA-binding protein with serine-rich domain 1;Rnps1;ortholog	RNA-BINDING PROTEIN WITH SERINE-RICH DOMAIN 1 (PTHR15481:SF2)	RNA splicing factor(PC00148)	Mus musculus
MOUSE|MGI=MGI=1860086|UniProtKB=Q9Z2L7	CRLF3	Cytokine receptor-like factor 3;Crlf3;ortholog	CYTOKINE RECEPTOR-LIKE FACTOR 3 (PTHR23389:SF12)	DNA binding protein(PC00009)	Mus musculus
MOUSE|MGI=MGI=1917128|UniProtKB=P62307	RUXF	Small nuclear ribonucleoprotein F;Snrpf;ortholog	SMALL NUCLEAR RIBONUCLEOPROTEIN F (PTHR11021:SF0)	RNA splicing factor(PC00148)	Mus musculus
MOUSE|MGI=MGI=1925901|UniProtKB=P62313	LSM6	U6 snRNA-associated Sm-like protein LSm6;Lsm6;ortholog	U6 SNRNA-ASSOCIATED SM-LIKE PROTEIN LSM6 (PTHR11021:SF1)	RNA splicing factor(PC00148)	Mus musculus
MOUSE|MGI=MGI=1915261|UniProtKB=P62309	RUXG	Small nuclear ribonucleoprotein G;Snrpg;ortholog	SMALL NUCLEAR RIBONUCLEOPROTEIN G-RELATED (PTHR10553:SF26)	RNA splicing factor(PC00148)	Mus musculus
MOUSE|MGI=MGI=1919016|UniProtKB=Q9CW46	RAVR1	Ribonucleoprotein PTB-binding 1;Raver1;ortholog	RIBONUCLEOPROTEIN PTB-BINDING 1 (PTHR23189:SF46)	RNA binding protein(PC00031)	Mus musculus
MOUSE|MGI=MGI=105380|UniProtKB=P97310	MCM2	DNA replication licensing factor MCM2;Mcm2;ortholog	DNA REPLICATION LICENSING FACTOR MCM2 (PTHR11630:SF44)	DNA binding protein(PC00009)	Mus musculus
MOUSE|MGI=MGI=102779|UniProtKB=P39749	FEN1	Flap endonuclease 1;Fen1;ortholog	FLAP ENDONUCLEASE 1 (PTHR11081:SF9)	exodeoxyribonuclease(PC00098)	Mus musculus
MOUSE|MGI=MGI=1930948|UniProtKB=Q9CY66	GAR1	H/ACA ribonucleoprotein complex subunit 1;Gar1;ortholog	H/ACA RIBONUCLEOPROTEIN COMPLEX SUBUNIT 1 (PTHR23237:SF6)	RNA binding protein(PC00031)	Mus musculus
MOUSE|MGI=MGI=1922946|UniProtKB=Q91YR7	PRP6	Pre-mRNA-processing factor 6;Prpf6;ortholog	PRE-MRNA-PROCESSING FACTOR 6 (PTHR11246:SF1)	RNA splicing factor(PC00148)	Mus musculus
MOUSE|MGI=MGI=893597|UniProtKB=P97376	FRG1	Protein FRG1;Frg1;ortholog	PROTEIN FRG1 (PTHR12928:SF3)	RNA splicing factor(PC00148)	Mus musculus
MOUSE|MGI=MGI=1298398|UniProtKB=Q61881	MCM7	DNA replication licensing factor MCM7;Mcm7;ortholog	DNA REPLICATION LICENSING FACTOR MCM7 (PTHR11630:SF26)	DNA binding protein(PC00009)	Mus musculus
MOUSE|MGI=MGI=1890165|UniProtKB=Q6ZQ58	LARP1	La-related protein 1;Larp1;ortholog	LA-RELATED PROTEIN 1 (PTHR22792:SF51)	RNA binding protein(PC00031)	Mus musculus
MOUSE|MGI=MGI=1921076|UniProtKB=Q8BG81	PDIP3	Polymerase delta-interacting protein 3;Poldip3;ortholog	POLYMERASE DELTA-INTERACTING PROTEIN 3 (PTHR19965:SF71)	RNA binding protein(PC00031)	Mus musculus
MOUSE|MGI=MGI=107169|UniProtKB=P16254	SRP14	Signal recognition particle 14 kDa protein;Srp14;ortholog	SIGNAL RECOGNITION PARTICLE 14 KDA PROTEIN (PTHR12013:SF0)	RNA binding protein(PC00031)	Mus musculus
MOUSE|MGI=MGI=1891690|UniProtKB=Q7TMK9	HNRPQ	Heterogeneous nuclear ribonucleoprotein Q;Syncrip;ortholog	HETEROGENEOUS NUCLEAR RIBONUCLEOPROTEIN Q (PTHR21245:SF11)	RNA binding protein(PC00031)	Mus musculus
MOUSE|MGI=MGI=2136773|UniProtKB=Q8C5N3	CWC22	Pre-mRNA-splicing factor CWC22 homolog;Cwc22;ortholog	PRE-MRNA-SPLICING FACTOR CWC22 HOMOLOG (PTHR18034:SF3)	RNA processing factor(PC00147)	Mus musculus
MOUSE|MGI=MGI=1913961|UniProtKB=P70122	SBDS	Ribosome maturation protein SBDS;Sbds;ortholog	RIBOSOME MATURATION PROTEIN SBDS (PTHR10927:SF1)	RNA binding protein(PC00031)	Mus musculus
MOUSE|MGI=MGI=2388280|UniProtKB=Q8CFI7	RPB2	DNA-directed RNA polymerase II subunit RPB2;Polr2b;ortholog	DNA-DIRECTED RNA POLYMERASE II SUBUNIT RPB2 (PTHR20856:SF7)	DNA-directed RNA polymerase(PC00019)	Mus musculus
MOUSE|MGI=MGI=1915208|UniProtKB=Q6NV83	SR140	U2 snRNP-associated SURP motif-containing	U2 SNRNP-ASSOCIATED SURP MOTIF-CONTAINING	RNA processing factor(PC00147)	Mus musculus

		protein;U2surp;ortholog	PROTEIN (PTHR23140:SF0)		
MOUSE|MGI=MGI=1196294|UniProtKB=Q91WJ8	FUBP1	Far upstream element-binding protein 1;Fubp1;ortholog	FAR UPSTREAM ELEMENT-BINDING PROTEIN 1 (PTHR10288:SF99)	RNA binding protein(PC00031)	Mus musculus
MOUSE|MGI=MGI=1914384|UniProtKB=Q9D6Z1	NOP56	Nucleolar protein 56;Nop56;ortholog	NUCLEOLAR PROTEIN 56 (PTHR10894:SF0)	RNA binding protein(PC00031)	Mus musculus
MOUSE|MGI=MGI=1915469|UniProtKB=Q9CQF3	CPSF5	Cleavage and polyadenylation specificity factor subunit 5;Nudt21;ortholog	CLEAVAGE AND POLYADENYLATION SPECIFICITY FACTOR SUBUNIT 5 (PTHR13047:SF0)	RNA splicing factor(PC00148)	Mus musculus
MOUSE|MGI=MGI=1339973|UniProtKB=Q9Z1X4	ILF3	Interleukin enhancer-binding factor 3;Ilf3;ortholog	INTERLEUKIN ENHANCER-BINDING FACTOR 3 (PTHR45762:SF4)	RNA binding protein(PC00031)	Mus musculus
MOUSE|MGI=MGI=1201779|UniProtKB=P70333	HNRH2	Heterogeneous nuclear ribonucleoprotein H2;Hnrnph2;ortholog	HETEROGENEOUS NUCLEAR RIBONUCLEOPROTEIN H2 (PTHR13976:SF33)	RNA splicing factor(PC00148)	Mus musculus
MOUSE|MGI=MGI=1928482|UniProtKB=Q9JKP5	MBNL1	Muscleblind-like protein 1;Mbnl1;ortholog	MUSCLEBLIND-LIKE PROTEIN 1 (PTHR12675:SF7)	RNA splicing factor(PC00148)	Mus musculus
MOUSE|MGI=MGI=88431|UniProtKB=P53996	CNBP	Cellular nucleic acid-binding protein;Cnbp;ortholog	CELLULAR NUCLEIC ACID-BINDING PROTEIN (PTHR23002:SF67)	nucleic acid binding protein(PC00171)	Mus musculus
MOUSE|MGI=MGI=1341044|UniProtKB=O08583	THOC4	THO complex subunit 4;Alyref;ortholog	THO COMPLEX SUBUNIT 4 (PTHR19965:SF35)	RNA binding protein(PC00031)	Mus musculus
MOUSE|MGI=MGI=1913806|UniProtKB=Q9D6N5	NC2A	Dr1-associated corepressor;Drap1;ortholog	DR1-ASSOCIATED COREPRESSOR (PTHR10252:SF5)	DNA binding protein(PC00009)	Mus musculus
MOUSE|MGI=MGI=1929092|UniProtKB=Q8C2Q3	RBM14	RNA-binding protein 14;Rbm14;ortholog	RNA-BINDING PROTEIN 14 (PTHR23147:SF53)	RNA splicing factor(PC00148)	Mus musculus
MOUSE|MGI=MGI=1935129|UniProtKB=Q91ZW3	SMCA5	SWI/SNF-related matrix-associated actin-dependent regulator of chromatin subfamily A member 5;Smarca5;ortholog	SWI/SNF-RELATED MATRIX-ASSOCIATED ACTIN-DEPENDENT REGULATOR OF CHROMATIN SUBFAMILY A MEMBER 5 (PTHR10799:SF879)	DNA helicase(PC00011)	Mus musculus
MOUSE|MGI=MGI=1289341|UniProtKB=Q921M3	SF3B3	Splicing factor 3B subunit 3;Sf3b3;ortholog	SPLICING FACTOR 3B SUBUNIT 3 (PTHR10644:SF1)	RNA processing factor(PC00147)	Mus musculus
MOUSE|MGI=MGI=1919794|UniProtKB=Q8BTW3	EXOS6	Exosome complex component MTR3;Exosc6;ortholog	EXOSOME COMPLEX COMPONENT MTR3 (PTHR11953:SF2)	exoribonuclease(PC00099)	Mus musculus
MOUSE|MGI=MGI=1917829|UniProtKB=Q6NZF1	ZC11A	Zinc finger CCCH domain-containing protein 11A;Zc3h11a;ortholog	ZINC FINGER CCCH DOMAIN-CONTAINING PROTEIN 11A (PTHR15725:SF2)	RNA processing factor(PC00147)	Mus musculus
MOUSE|MGI=MGI=98284|UniProtKB=Q62093	SRSF2	Serine/arginine-rich splicing factor 2;Srsf2;ortholog	SERINE/ARGININE-RICH SPLICING FACTOR 2 (PTHR23147:SF119)	RNA splicing factor(PC00148)	Mus musculus
MOUSE|MGI=MGI=1891840|UniProtKB=Q3UYV9	NCBP1	Nuclear cap-binding protein subunit 1;Ncbp1;ortholog	NUCLEAR CAP-BINDING PROTEIN SUBUNIT 1 (PTHR12412:SF2)	RNA splicing factor(PC00148)	Mus musculus
MOUSE|MGI=MGI=1340045|UniProtKB=Q9JMD0	ZN207	BUB3-interacting and GLEBS motif-containing protein ZNF207;Znf207;ortholog	BUB3-INTERACTING AND GLEBS MOTIF-CONTAINING PROTEIN ZNF207 (PTHR23215:SF0)	DNA binding protein(PC00009)	Mus musculus
MOUSE|MGI=MGI=1099786|UniProtKB=O35286	DHX15	Pre-mRNA-splicing factor ATP-dependent RNA helicase DHX15;Dhx15;ortholog	PRE-MRNA-SPLICING FACTOR ATP-DEPENDENT RNA HELICASE DHX15 (PTHR18934:SF95)	RNA helicase(PC00032)	Mus musculus
MOUSE|MGI=MGI=98341|UniProtKB=Q62376	RU17	U1 small nuclear ribonucleoprotein 70 kDa;Snrnp70;ortholog	U1 SMALL NUCLEAR RIBONUCLEOPROTEIN 70 KDA (PTHR13952:SF5)	RNA splicing factor(PC00148)	Mus musculus
MOUSE|MGI=MGI=88192|UniProtKB=Q3TKT4	SMCA4	Transcription activator BRG1;Smarca4;ortholog	TRANSCRIPTION ACTIVATOR BRG1 (PTHR10799:SF76)	DNA helicase(PC00011)	Mus musculus
MOUSE|MGI=MGI=1343463|UniProtKB=Q9WVA3	BUB3	Mitotic checkpoint protein BUB3;Bub3;ortholog	MITOTIC CHECKPOINT PROTEIN BUB3 (PTHR10971:SF5)	RNA binding protein(PC00031)	Mus musculus
MOUSE|MGI=MGI=1858230|UniProtKB=Q9JLI8	SART3	Squamous cell carcinoma antigen recognized by T-cells 3;Sart3;ortholog	SQUAMOUS CELL CARCINOMA ANTIGEN RECOGNIZED BY T-CELLS 3 (PTHR15481:SF5)	RNA splicing factor(PC00148)	Mus musculus
MOUSE|MGI=MGI=98344|UniProtKB=P62315	SMD1	Small nuclear ribonucleoprotein Sm D1;Snrpd1;ortholog	SMALL NUCLEAR RIBONUCLEOPROTEIN SM D1 (PTHR23338:SF18)	RNA splicing factor(PC00148)	Mus musculus
MOUSE|MGI=MGI=1298227|UniProtKB=P97311	MCM6	DNA replication licensing factor MCM6;Mcm6;ortholog	DNA REPLICATION LICENSING FACTOR MCM6	DNA binding protein(PC00009)	Mus musculus

			(PTHR11630:SF73)		
MOUSE|MGI=MGI=1913604|UniProtKB=Q9CSN1	SNW1	SNW domain-containing protein 1;Snw1;ortholog	SNW DOMAIN-CONTAINING PROTEIN 1 (PTHR12096:SF0)	RNA splicing factor(PC00148)	Mus musculus
MOUSE|MGI=MGI=98353|UniProtKB=Q9QX47	SON	Protein SON;Son;ortholog	PROTEIN SON (PTHR46528:SF1)	RNA splicing factor(PC00148)	Mus musculus
MOUSE|MGI=MGI=103197|UniProtKB=P49718	MCM5	DNA replication licensing factor MCM5;Mcm5;ortholog	DNA REPLICATION LICENSING FACTOR MCM5 (PTHR11630:SF42)	DNA binding protein(PC00009)	Mus musculus
MOUSE|MGI=MGI=98884|UniProtKB=Q9D883	U2AF1	Splicing factor U2AF 35 kDa subunit;U2af1;ortholog	SPLICING FACTOR U2AF 35 KDA SUBUNIT-RELATED (PTHR12620:SF11)	RNA splicing factor(PC00148)	Mus musculus
MOUSE|MGI=MGI=101845|UniProtKB=P25206	MCM3	DNA replication licensing factor MCM3;Mcm3;ortholog	DNA REPLICATION LICENSING FACTOR MCM3 (PTHR11630:SF72)	DNA binding protein(PC00009)	Mus musculus
MOUSE|MGI=MGI=98287|UniProtKB=O35326	SRSF5	Serine/arginine-rich splicing factor 5;Srsf5;ortholog	SERINE/ARGININE-RICH SPLICING FACTOR 5 (PTHR23147:SF68)	RNA splicing factor(PC00148)	Mus musculus
MOUSE|MGI=MGI=98788|UniProtKB=Q04750	TOP1	DNA topoisomerase 1;Top1;ortholog	DNA TOPOISOMERASE 1 (PTHR10290:SF5)	DNA topoisomerase(PC00017)	Mus musculus
MOUSE|MGI=MGI=1923848|UniProtKB=Q3THK3	T2FA	General transcription factor IIF subunit 1;Gtf2f1;ortholog	GENERAL TRANSCRIPTION FACTOR IIF SUBUNIT 1 (PTHR13011:SF0)	general transcription factor(PC00259)	Mus musculus
MOUSE|MGI=MGI=1858303|UniProtKB=Q52KI8	SRRM1	Serine/arginine repetitive matrix protein 1;Srrm1;ortholog	SERINE/ARGININE REPETITIVE MATRIX PROTEIN 1 (PTHR23148:SF0)	RNA processing factor(PC00147)	Mus musculus
MOUSE|MGI=MGI=1913754|UniProtKB=Q3TLH4	PRC2C	Protein PRRC2C;Prrc2c;ortholog	PROTEIN PRRC2C (PTHR14038:SF6)	RNA binding protein(PC00031)	Mus musculus
MOUSE|MGI=MGI=1336214|UniProtKB=Q3U0V1	FUBP2	Far upstream element-binding protein 2;Khsrp;ortholog	FAR UPSTREAM ELEMENT-BINDING PROTEIN 2 (PTHR10288:SF101)	RNA binding protein(PC00031)	Mus musculus
MOUSE|MGI=MGI=108177|UniProtKB=O70133	DHX9	ATP-dependent RNA helicase A;Dhx9;ortholog	ATP-DEPENDENT RNA HELICASE A (PTHR18934:SF119)	RNA helicase(PC00032)	Mus musculus
MOUSE|MGI=MGI=2442040|UniProtKB=P97379	G3BP2	Ras GTPase-activating protein-binding protein 2;G3bp2;ortholog	RAS GTPASE-ACTIVATING PROTEIN-BINDING PROTEIN 2 (PTHR10693:SF10)	RNA binding protein(PC00031)	Mus musculus
MOUSE|MGI=MGI=2138741|UniProtKB=Q9Z2X1	HNRPF	Heterogeneous nuclear ribonucleoprotein F;Hnrnpf;ortholog	HETEROGENEOUS NUCLEAR RIBONUCLEOPROTEIN F (PTHR13976:SF32)	RNA splicing factor(PC00148)	Mus musculus
MOUSE|MGI=MGI=99256|UniProtKB=Q8VDJ3	VIGLN	Vigilin;Hdlbp;ortholog	VIGILIN (PTHR10627:SF34)	RNA binding protein(PC00031)	Mus musculus
MOUSE|MGI=MGI=1913305|UniProtKB=P59708	SF3B6	Splicing factor 3B subunit 6;Sf3b6;ortholog	SPLICING FACTOR 3B SUBUNIT 6 (PTHR12785:SF7)	RNA splicing factor(PC00148)	Mus musculus
MOUSE|MGI=MGI=1861461|UniProtKB=P43276	H15	Histone H1.5;Hist1h1b;ortholog	HISTONE H1.5 (PTHR11467:SF24)	histone(PC00118)	Mus musculus
MOUSE|MGI=MGI=2442637|UniProtKB=Q569Z6	TR150	Thyroid hormone receptor-associated protein 3;Thrap3;ortholog	THYROID HORMONE RECEPTOR-ASSOCIATED PROTEIN 3 (PTHR15268:SF16)	nucleic acid binding protein(PC00171)	Mus musculus
MOUSE|MGI=MGI=1917580|UniProtKB=Q8K019	BCLF1	Bcl-2-associated transcription factor 1;Bclaf1;ortholog	BCL-2-ASSOCIATED TRANSCRIPTION FACTOR 1 (PTHR15268:SF4)	nucleic acid binding protein(PC00171)	Mus musculus
MOUSE|MGI=MGI=1914715|UniProtKB=Q8K4Z5	SF3A1	Splicing factor 3A subunit 1;Sf3a1;ortholog	SPLICING FACTOR 3A SUBUNIT 1 (PTHR15316:SF1)	RNA splicing factor(PC00148)	Mus musculus
MOUSE|MGI=MGI=98342|UniProtKB=P27048	RSMB	Small nuclear ribonucleoprotein-associated protein B;Snrpb;ortholog	SMALL NUCLEAR RIBONUCLEOPROTEIN-ASSOCIATED PROTEINS B AND B' (PTHR10701:SF15)	RNA splicing factor(PC00148)	Mus musculus
MOUSE|MGI=MGI=1928895|UniProtKB=Q9JL16	ISG20	Interferon-stimulated gene 20 kDa protein;Isg20;ortholog	INTERFERON-STIMULATED GENE 20 KDA PROTEIN (PTHR12801:SF59)	exoribonuclease(PC00099)	Mus musculus
MOUSE|MGI=MGI=98423|UniProtKB=P32067	LA	Lupus La protein homolog;Ssb;ortholog	LUPUS LA PROTEIN HOMOLOG (PTHR22792:SF140)	RNA binding protein(PC00031)	Mus musculus
MOUSE|MGI=MGI=2179381|UniProtKB=Q99PV0	PRP8	Pre-mRNA-processing-splicing factor 8;Prpf8;ortholog	PRE-MRNA-PROCESSING-SPLICING FACTOR 8 (PTHR11140:SF0)	RNA splicing factor(PC00148)	Mus musculus
MOUSE|MGI=MGI=107995|UniProtKB=Q9EPU0	RENT1	Regulator of nonsense transcripts 1;Upf1;ortholog	REGULATOR OF NONSENSE TRANSCRIPTS 1 (PTHR10887:SF388)	RNA helicase(PC00032);DNA helicase(PC00011)	Mus musculus
MOUSE|MGI=MGI=98285|UniProtKB=P84104	SRSF3	Serine/arginine-rich splicing factor 3;Srsf3;ortholog	SERINE/ARGININE-RICH SPLICING FACTOR 3 (PTHR23147:SF121)	RNA splicing factor(PC00148)	Mus musculus

MOUSE|MGI=MGI=98086|UniProtKB=P08775	RPB1	DNA-directed RNA polymerase II subunit RPB1;Polr2a;ortholog	DNA-DIRECTED RNA POLYMERASE II SUBUNIT RPB1 (PTHR19376:SF37)	DNA-directed RNA polymerase(PC00019)	Mus musculus
MOUSE|MGI=MGI=1913618|UniProtKB=Q9D7H3	RTCA	RNA 3'-terminal phosphate cyclase;RtcA;ortholog	RNA 3'-TERMINAL PHOSPHATE CYCLASE (PTHR11096:SF0)	RNA binding protein(PC00031)	Mus musculus
MOUSE|MGI=MGI=1859328|UniProtKB=Q9QXK7	CPSF3	Cleavage and polyadenylation specificity factor subunit 3;Cpsf3;ortholog	CLEAVAGE AND POLYADENYLATION SPECIFICITY FACTOR SUBUNIT 3 (PTHR11203:SF11)	RNA processing factor(PC00147)	Mus musculus
MOUSE|MGI=MGI=1933184|UniProtKB=Q6DFW4	NOP58	Nucleolar protein 58;Nop58;ortholog	NUCLEOLAR PROTEIN 58 (PTHR10894:SF1)	RNA binding protein(PC00031)	Mus musculus
MOUSE|MGI=MGI=1915433|UniProtKB=Q9D287	SPF27	Pre-mRNA-splicing factor SPF27;Bcas2;ortholog	PRE-MRNA-SPLICING FACTOR SPF27 (PTHR13296:SF0)	RNA splicing factor(PC00148)	Mus musculus
MOUSE|MGI=MGI=1855690|UniProtKB=Q62189	SNRPA	U1 small nuclear ribonucleoprotein A;Snrpa;ortholog	U1 SMALL NUCLEAR RIBONUCLEOPROTEIN A (PTHR10501:SF63)	RNA splicing factor(PC00148)	Mus musculus
MOUSE|MGI=MGI=1861727|UniProtKB=Q9ESX5	DKC1	H/ACA ribonucleoprotein complex subunit DKC1;Dkc1;ortholog	H/ACA RIBONUCLEOPROTEIN COMPLEX SUBUNIT DKC1 (PTHR23127:SF0)	centromere DNA-binding protein(PC00071)	Mus musculus
MOUSE|MGI=MGI=1916238|UniProtKB=Q8CCF0	PRP31	U4/U6 small nuclear ribonucleoprotein Prp31;Prpf31;ortholog	U4/U6 SMALL NUCLEAR RIBONUCLEOPROTEIN PRP31 (PTHR13904:SF0)	RNA splicing factor(PC00148)	Mus musculus
MOUSE|MGI=MGI=104805|UniProtKB=Q9CQI7	RU2B	U2 small nuclear ribonucleoprotein B'';Snrpb2;ortholog	U2 SMALL NUCLEAR RIBONUCLEOPROTEIN B'' (PTHR10501:SF61)	RNA splicing factor(PC00148)	Mus musculus
MOUSE|MGI=MGI=2140494|UniProtKB=Q8R3G1	PP1R8	Nuclear inhibitor of protein phosphatase 1;Ppp1r8;ortholog	NUCLEAR INHIBITOR OF PROTEIN PHOSPHATASE 1 (PTHR23308:SF28)	RNA splicing factor(PC00148)	Mus musculus
MOUSE|MGI=MGI=1915525|UniProtKB=Q8VEE4	RFA1	Replication protein A 70 kDa DNA-binding subunit;Rpa1;ortholog	REPLICATION PROTEIN A 70 KDA DNA-BINDING SUBUNIT (PTHR23273:SF4)	DNA binding protein(PC00009)	Mus musculus
MOUSE|MGI=MGI=1922312|UniProtKB=Q9D554	SF3A3	Splicing factor 3A subunit 3;Sf3a3;ortholog	SPLICING FACTOR 3A SUBUNIT 3 (PTHR12786:SF2)	RNA splicing factor(PC00148)	Mus musculus

Table S3. Highly correlated targets of the miRNAs with higher expression levels in Kupffer cells that are predicted by miRNet.

Name	Hits	Pval	adj.Pval
Metabolic pathways	785	6.87e-20	6.87e-18
Pathways in cancer	276	9.17e-17	4.585e-15
Proteoglycans in cancer	155	8.67e-15	2.89e-13
Focal adhesion	154	2.3e-13	5.75e-12
Axon guidance	103	7.52e-13	1.504e-11
Endocytosis	192	1.42e-11	2.366667e-10
PI3K-Akt signaling pathway	234	2.35e-11	3.357143e-10
Thyroid hormone signaling pathway	91	2.37e-10	2.9625e-9
Insulin resistance	87	2.88e-10	3.2e-9
T cell receptor signaling pathway	83	3.41e-10	3.41e-9
Rap1 signaling pathway	150	4.83e-10	4.390909e-9
FoxO signaling pathway	101	6.02e-10	4.7e-9
Protein processing in endoplasmic reticulum	122	6.11e-10	4.7e-9
Chagas disease (American trypanosomiasis)	81	8.88e-10	6.342857e-9
Neurotrophin signaling pathway	93	1.03e-9	6.866667e-9
ErbB signaling pathway	70	2.03e-9	1.26875e-8
Prostate cancer	71	3.15e-9	1.852941e-8

Osteoclast differentiation	94	5.98e-9	3.322222e-8
MAPK signaling pathway	169	1.23e-8	6.473684e-8
Sphingolipid signaling pathway	92	1.39e-8	6.95e-8
Phosphatidylinositol signaling system	75	1.47e-8	7,00E-08
Toxoplasmosis	85	1.65e-8	7.391304e-8
Adherens junction	60	1.7e-8	7.391304e-8
TNF signaling pathway	83	1.9e-8	7.916667e-8
Cell cycle	92	2.72e-8	1.088e-7
Insulin signaling pathway	101	5.98e-8	2.3e-7
Renal cell carcinoma	55	7.99e-8	2.910714e-7
TGF-beta signaling pathway	66	8.15e-8	2.910714e-7
HIF-1 signaling pathway	81	9.11e-8	3.141379e-7
Lysosome	90	1.16e-7	3.866667e-7
Glucagon signaling pathway	76	1.89e-7	6.096774e-7
Pancreatic cancer	53	2.17e-7	6.78125e-7
Fc gamma R-mediated phagocytosis	67	2.5e-7	7.575758e-7
Inositol phosphate metabolism	55	4.95e-7	0.000001455882
Colorectal cancer	51	5.82e-7	0.000001662857
mTOR signaling pathway	49	6.19e-7	0.000001719444
Hippo signaling pathway	106	7.39e-7	0.000001960526
HTLV-I infection	179	7.45e-7	0.000001960526
AMPK signaling pathway	91	8.29e-7	0.000002125641
Long-term potentiation	52	8.72e-7	0.00000218
Small cell lung cancer	64	9.33e-7	0.00000227561
Platelet activation	92	9.94e-7	0.000002366667
Ubiquitin mediated proteolysis	99	0.00000113	0.000002627907
Tuberculosis	118	0.0000015	0.0000034
Ras signaling pathway	149	0.00000153	0.0000034
Chronic myeloid leukemia	56	0.0000016	0.000003478261
Prion diseases	30	0.00000227	0.000004829787
Endometrial cancer	42	0.00000306	0.000006375
N-Glycan biosynthesis	40	0.00000318	0.000006489796
Pertussis	56	0.00000342	0.00000684
Leishmaniasis	50	0.00000507	0.00000975
Glioma	50	0.00000507	0.00000975
Oocyte meiosis	80	0.00000528	0.000009796296
B cell receptor signaling pathway	55	0.00000529	0.000009796296
Regulation of actin cytoskeleton	139	0.00000843	0.00001532727
Hepatitis B	98	0.0000107	0.00001910714
Notch signaling pathway	39	0.0000135	0.00002368421
Apoptosis	59	0.0000149	0.00002568966
Acute myeloid leukemia	44	0.0000161	0.00002728814
Dopaminergic synapse	91	0.0000169	0.00002816667
Salmonella infection	57	0.0000176	0.00002885246
GnRH signaling pathway	63	0.0000192	0.00003096774
Non-small cell lung cancer	43	0.0000254	0.00004031746
Epstein-Barr virus infection	136	0.0000267	0.00004171875

Biosynthesis of amino acids	57	0.0000329	0.00005061538
Gap junction	61	0.0000411	0.00006227273
Wnt signaling pathway	95	0.0000419	0.00006253731
ECM-receptor interaction	62	0.0000495	0.00007279412
Non-alcoholic fatty liver disease (NAFLD)	102	0.0000571	0.00008275362
Circadian rhythm	26	0.0000755	0.0001078571
Choline metabolism in cancer	69	0.0000915	0.0001288732
cAMP signaling pathway	124	0.000115	0.0001597222
Carbon metabolism	78	0.000118	0.0001616438
Amphetamine addiction	49	0.000123	0.0001662162
Signaling pathways regulating pluripotency of stem cells	91	0.000138	0.000184
Leukocyte transendothelial migration	80	0.000149	0.0001960526
Bladder cancer	32	0.000164	0.000212987
Pyrimidine metabolism	70	0.000167	0.0002141026
p53 signaling pathway	48	0.000182	0.0002275
Fc epsilon RI signaling pathway	48	0.000182	0.0002275
Primary immunodeficiency	28	0.000199	0.000245679
Central carbon metabolism in cancer	47	0.000268	0.0003228916
cGMP-PKG signaling pathway	108	0.000268	0.0003228916
Progesterone-mediated oocyte maturation	60	0.000282	0.0003357143
Steroid biosynthesis	17	0.000309	0.0003635294
Dorso-ventral axis formation	21	0.000376	0.0004372093
PPAR signaling pathway	56	0.000418	0.0004804598
Estrogen signaling pathway	65	0.000531	0.0006011236
Prolactin signaling pathway	51	0.000535	0.0006011236
Measles	87	0.000607	0.0006744444
Renin secretion	49	0.000652	0.0007086957
Bile secretion	49	0.000652	0.0007086957
VEGF signaling pathway	42	0.000997	0.001062766
Inflammatory mediator regulation of TRP channels	80	0.000999	0.001062766
Influenza A	105	0.00104	0.00109375
Phospholipase D signaling pathway	90	0.00105	0.00109375
Alzheimer's disease	108	0.00122	0.001257732
Huntington's disease	120	0.00126	0.001285714
Basal transcription factors	32	0.00144	0.001454545
Cocaine addiction	35	0.00148	0.00148

Table S4. The list of mRNAs differ more than 2 times in Kupffer cells

Probe Name	FC KCs vs. MNCs	P value of: KCs vs. MNCs
C8a	726,3	1E-08
Hc	576,2	1E-08
C9	341,6	1E-08
Mbl2	174,4	1E-08
Masp1	167,1	1E-08
Crp	128,8	1E-08
C8b	127,8	2,01E-06
Kng1	116,0	3E-08
C1s	105,3	1E-08
C3	46,7	1E-08
C6	34,9	3,1E-07
Masp2	29,3	1,91E-06
Il1r1	24,5	1,67E-05
C2	21,3	1E-08
Ifng	13,4	4,5E-07
Cxcl9	11,6	0,000602
Iigp1	8,8	0,000105
Il17a	8,2	4,09E-05
Arg1	7,9	1,32E-05
Flt1	7,6	2,24E-05
Ccr3	7,3	4,15E-06
Csf2	7,1	6,3E-07
Ccl4	6,1	1,64E-06
Mafg	5,7	0,000355
Jun	5,0	1,48E-06
Cxcl10	4,9	0,000226
Nos2	4,5	0,000106
Ccl3	4,1	3,3E-07
Ifit3	4,0	4,36E-06
Fos	3,9	1,33E-06
Tgfb3	3,7	9,54E-06
Tnfsf14	3,4	1,44E-06
Ccl5	3,2	0,002432
Ifit1	2,9	0,009291
Cfb	2,9	5,15E-05
Stat2	2,8	9,72E-06
Mx2	2,7	0,000416
Nfe2l2	2,7	0,001077
Oasl1	2,6	0,027382
Ccr4	2,5	0,001074
Cysltr2	2,4	0,001512
Ifi44	2,4	7,61E-05

Tnf	2,4	0,000141
Csf1	2,3	0,000601
Ifit2	2,3	0,000109
Il18rap	2,3	0,000671
Cd163	2,2	0,011733
Map2k6	2,1	0,000327
Keap1	2,0	2E-07
Cd40lg	2,0	0,00086
C1ra	2,0	0,004424
Tlr4	-2,0	0,000299
Il1a	-2,0	0,096796
Cd86	-2,2	0,001751
Hmgb2	-2,2	0,000777
Tyrobp	-2,0	2,96E-05
Il12a	-2,4	0,004729
Tlr7	-2,5	0,001239
Ccl2	-2,6	0,109131
Ly96	-2,7	0,008737
Cebpb	-2,8	3,46E-05
C4a	-3,1	0,00048
Itgb2	-3,3	2,5E-07
C1qb	-3,5	0,00037
Ptgs1	-3,6	6E-05
Tlr2	-3,6	1,85E-05
Nlrp3	-3,7	7,46E-05
Tlr8	-3,8	4,84E-05
Cysltr1	-3,9	2,12E-05
Ptgir	-3,9	0,025186
Il6	-4,0	0,000177
C1qa	-4,5	0,000114
Chi3l3	-4,7	0,001325
C3ar1	-5,3	1,27E-06
Ccr1	-5,8	3,06E-05
Alox5	-5,8	1,9E-07
Ccl24	-6,7	6,34E-06
Ccl17	-7,1	0,000958
Alox12	-9,2	5,34E-06
Cxcl1	-26,4	1,01E-05
Cxcl2	-28,3	1,6E-07
Alox15	-32,2	1,1E-07
Tgfb2	-36,3	1E-08
Retnla	-999,7	1E-08

Table S5. The list of miRNAs upregulated in Kupffer cells

	Probe Name	FC of : KCs vs MNCs	P value of: KCs vs. MNCs
1	mmu-miR-122	211,2	0.00005
2	mmu-let-7d	14,65	0.04042
3	mmu-let-7b	13,54	0.03417
4	mmu-miR-1944	8,27	0.00439
5	mmu-let-7c	7,31	0.00699
6	mmu-miR-2141	5,62	0.00774
7	mmu-miR-466g	5,27	0.00039
8	mmu-miR-1224	4,72	0.03371
9	mmu-miR-714	3,92	0.01611
10	mmu-miR-376a	3,74	0.02910
11	mmu-let-7e	3,73	0.00316
12	mmu-miR-539	3,25	0.01387
13	mmu-miR-130b	3,08	0.02484
14	mmu-miR-16	3,06	0.03919
15	mmu-miR-101b	3,06	0.01488
16	mmu-miR-30b	2,79	0.03154
17	mmu-miR-2146	2,78	0.01376
18	mmu-miR-1929	2,78	0,02651
19	mmu-miR-200b	2,74	0.00212
20	mmu-let-7g	2,74	0.01035
21	mmu-miR-1187	2,72	0.02944
22	mmu-miR-190	2,67	0.01563
23	mmu-miR-202-5p	2,38	0.01630
24	mmu-miR-720	2,31	0.03067
